# CpCo–Benzoquinone Complexes: Convenient CpCo(I) Precatalysts—Synthesis and Reactivity

**DOI:** 10.1021/acs.organomet.6c00194

**Published:** 2026-08-29

**Authors:** Rahamdil Usman, Uwe Monkowius, Marko Hapke

**Affiliations:** † Institute for Catalysis (INCA), Johannes Kepler University Linz (JKU), Altenberger Strasse 69, A-4040 Linz, Austria; ‡ Institute of Inorganic Chemistry (IAC), JKU Linz, Altenberger Strasse 69, A-4040 Linz, Austria; § Leibniz Institute for Catalysis e.V. (LIKAT), Albert-Einstein-Strasse 29a, D-18059 Rostock, Germany

## Abstract

We report an improved, convenient, and general synthesis of the air-stable complex CpCo­(DQ) (DQ = duroquinone), originally described by Schrauzer and Thyret in 1963, and applied this general protocol to the preparation of a series of related CpCo­(*p*-benzoquinone) complexes with yields ranging from 35 to 96%. The complexes were characterized by single-crystal X-ray diffraction, and the bonding characteristics were compared to those of other CpCo–olefin complexes. Their catalytic performance was evaluated in cyclotrimerization reactions under both thermal and photochemical conditions for the synthesis of pyridine and benzene derivatives.

## Graphical abstract

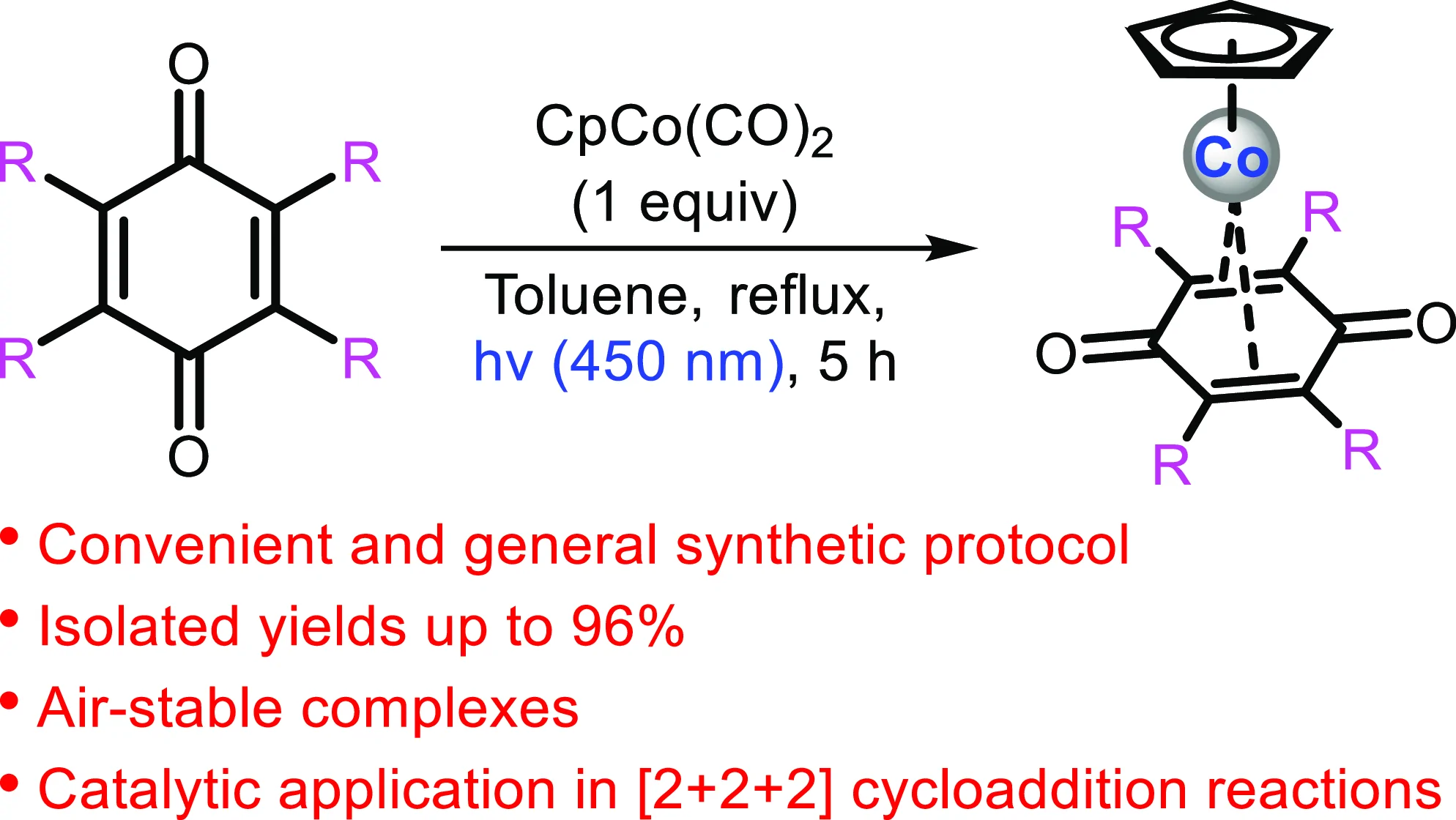

## Introduction

Precatalysts serve as key ingredients in reaction systems designed for catalytic transformations.^1^ In organometallic homogeneous catalysis, the generation of catalysts often relies on the *in situ* generation approach by mixing all components and activating the forming (pre)­catalyst, if required, e.g. by addition of reducing agents or an external stimulus like temperature or irradiation. Although *in situ* generation profits largely from its inherent flexibility in ligand/metal stoichiometry and component availability, well-defined, (air-)­stable, isolable precatalysts are application-friendly, and e.g., enable detailed mechanistic studies significantly more easily. Particularly given the importance of cross-coupling in organic, pharmaceutical and materials synthesis, considerable effort has focused on developing well-defined Pd and Ni precatalysts that balance stability with facile activation.^2^ For [2+2+2] cycloaddition reactions of alkynes and heterocumulenes,^3^ cobalt is one of the primary catalyst metals, exhibiting a rich structural chemistry of low-valent metal complexes, which are used as precatalysts for a large variety of transformations.^4^ The first generation of catalysts consists of the cyclopentadienyl (Cp)-Co fragment, stabilized by a variety of neutral donor ligands. Starting out from CpCo­(CO)_2_ as still one of the best known precatalysts introduced by Vollhardt et al.,^5^ other groups including Jonas,^6^ Aubert and Gandon^7^ and us^8^ have contributed novel precatalysts over the years and studied their reactivities and the required reaction conditions.

During studies on CpCo­(I)-complexes, we came across a communication published in 1963 by Schrauzer and Thyret, who described the facile synthesis of a rather stable **CpCo­(DQ)** (DQ = duroquinone) complex by treating CpCo­(CO)_2_ with DQ at elevated temperature in high-boiling solvent for several hours (Scheme 1).^9^

**Scheme 1:**
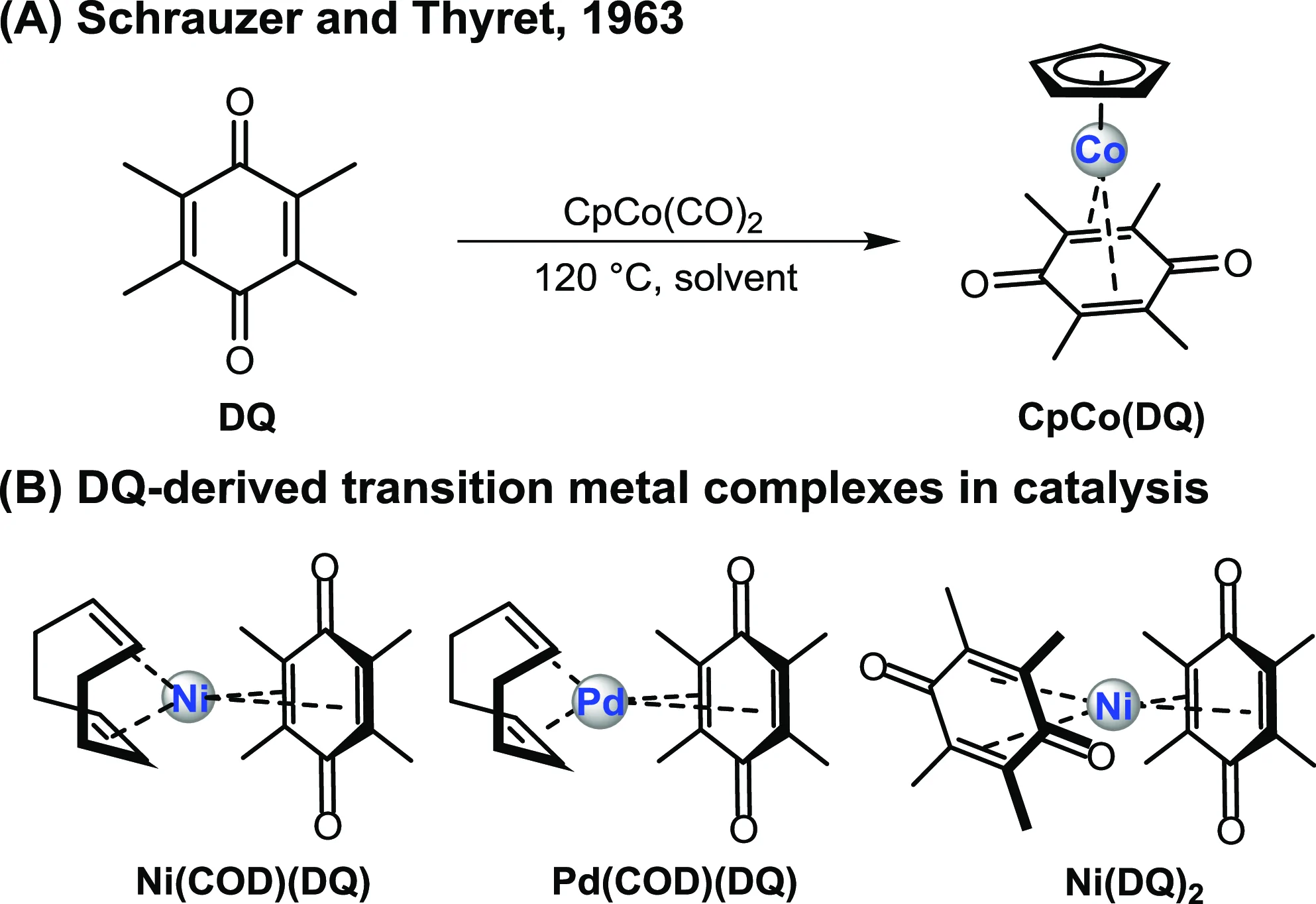
(A) Reported Synthesis of CpCo**(DQ)** by Schrauzer and Thyret, and (B) Reported DQ Complexes of Nickel and Palladium

The complex was described as highly polar and soluble in water, though no isolated yield was reported. The high stability of duroquinone complexes is particularly noteworthy, as this ligand system has recently been employed to stabilize low-valent Ni(0)- and Pd(0)-species,^10^ thereby expanding their application in various catalytic transformations (Scheme 1).^11^ The use of alkenes containing electron-withdrawing substituents allows excellent stabilization of electron-rich metal centers in low-oxidation states, enabling the insertion of the catalytically species into the catalytic cycle without requiring reduction. However, the tradeoff between precatalyst stabilization and enabling facile activation (i.e., ligand dissociation) is delicate. The strength of ligand coordination increases within a group, exemplified for the group 9 metals (Co, Rh, Ir) for ethylene as ligand,^12^ displaying the same trend as found for duroquinones earlier.^13^

Several years after the seminal work by Schrauzer and Thyret, a modified synthesis using UV irradiation in refluxing ethylcyclohexane was reported in 1972, affording only 16% crude product before purification.^14^ A decade later yet another approach toward **CpCo­(DQ)** was reported using cobaltocene as starting material, however, a yield of 12% and a reaction time of 34 h were reported.^15^ In 1985, a series of CpCo­(*p*-benzoquinone) complexes were synthesized via a three-step procedure starting from CpCo­(CO)_2_.^16^ Further alternative syntheses of CpCo­(*p*-benzoquinone) have been reported from the transformation of CpCo­(CO)_2_ with but-2-yne^17^ or substituted cyclopropenone,^18^ but these attempts suffer from low selectivity and afford multiple products. Therefore, so far, no practical and high-yielding synthesis with generality for substituted duroquinones has been documented. To overcome these limitations, we aimed to develop a practical one-step synthetic route, ideally providing high yields in desirably short reaction times and evaluate structural features of such complexes. In addition, the usefulness of such precatalysts should be exemplarily investigated in [2+2+2] cycloaddition reactions under thermal and photochemical conditions.

## Results and Discussion

Since the original synthesis of **CpCo­(DQ)** by Schrauzer and Thyret lacked detailed procedures, our initial attempt was the reproduction of the reported reaction conditions. However, even on careful experimentation under inert conditions, the refluxing of the solution of reactants in toluene for 24 h afforded only 30% yield of the complex (Scheme 2). Drawing from our prior experience with photochemical ligand exchange to prepare CpCo­(I)-precatalysts bearing electron-deficient olefins, we investigated the substitution of the CO ligands of CpCo­(CO)_2_ with DQ using blue LED irradiation under high temperatures. Gratifyingly, refluxing of the reaction solution under blue LED irradiation in a 1:1 ratio of DQ/CpCo­(CO)_2_ furnished **CpCo­(DQ)** in 92% yield within 5 h (Scheme 2). However, an attempt to lower the reaction temperature to 80 °C afforded only 15% yield of the product. Interestingly, switching from an open reflux setup to a closed vessel significantly lowered the yield to 33%. Details for the synthetic procedure are illustrated in the Supporting Information.

**Scheme 2:**
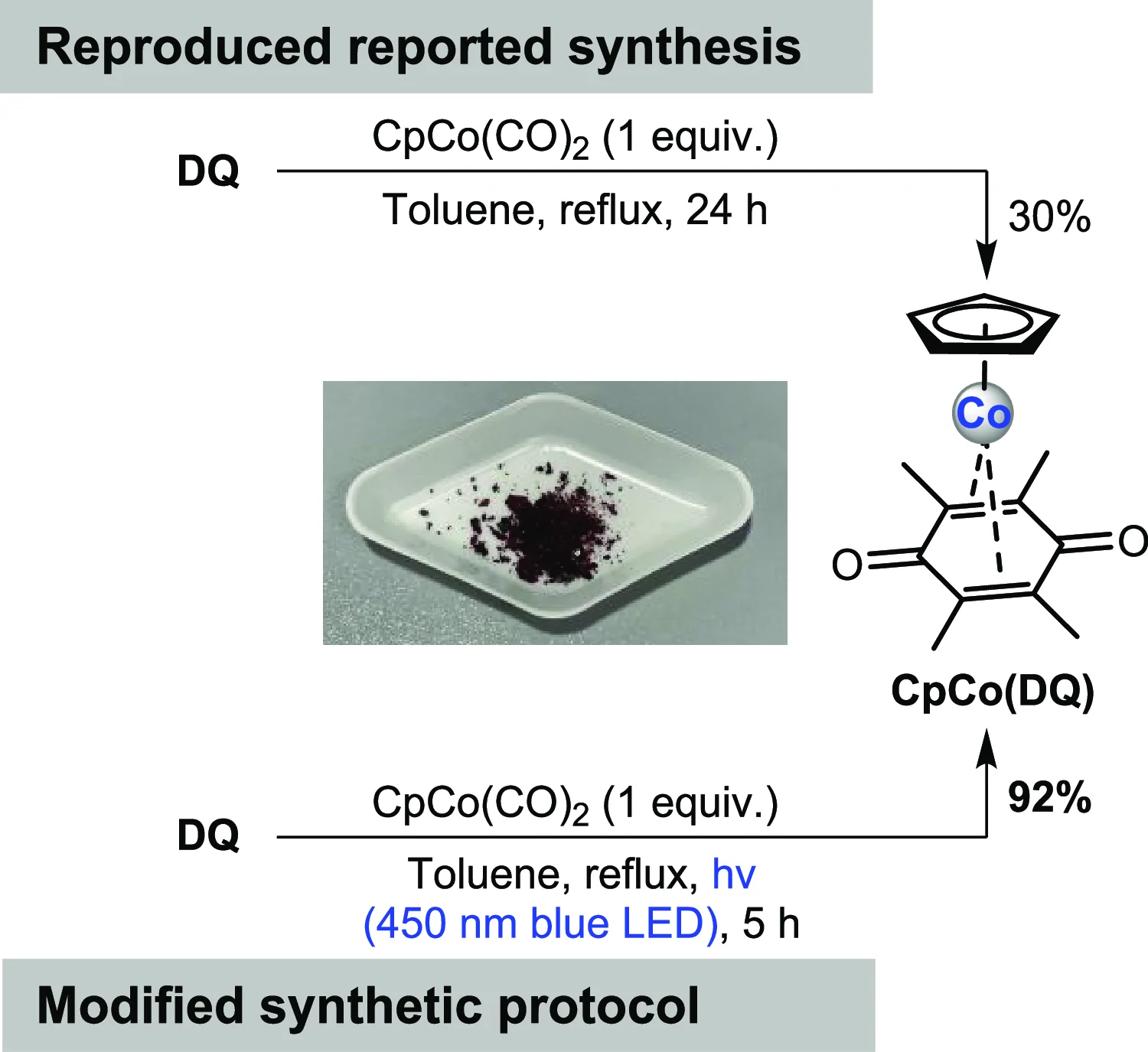
Reported Synthesis of CpCo**(DQ)** by Schrauzer and Thyret and the New Improved Synthetic Protocol

**CpCo­(DQ)** was obtained as a highly polar, hygroscopic, air-stable deep-red solid and it was possible to simply purify the complex by column chromatography. It shows poor solubility in common organic solvents but was found to be readily soluble in water (see the Supporting Information, Table S-1). The molecular structure was unambiguously confirmed by X-ray diffraction studies (vide infra). The observed stability of **CpCo­(DQ)** intrigued us and we set out to synthesize possibly better soluble derivatives with differently substituted *p*-benzoquinones via this improved protocol, taking advantage of commercial availability of substituted *para*-benzoquinones or available synthetic protocols.^19^

Therefore, we subjected a selection of substituted benzoquinones to the synthesis of CpCo­(*p*-benzoquinone) complexes by employing our improved synthetic protocol (Scheme 3). 2,3,5,6-Tetraethyl-1,4-benzoquinone (Et_4_Q) and 2,5-bis­(*tert*-butyl)-1,4-benzoquinone (*t*-Bu_2_Q) reacted cleanly to give **CpCo­(Et**_**4**_ **Q)** and **CpCo­(** ***t*** **-Bu**_**2**_ **Q)** complexes in 96% and 85% yields, respectively. In contrast, 2,5-diphenyl-1,4-benzoquinone (Ph_2_Q) afforded **CpCo­(Ph**_**2**_ **Q)**, albeit in 35% yield along with unidentified side products. All of the synthesized complexes are air-stable for months in the solid state (see Figures S2–S18, Supporting Information) and were purified by column chromatography. They are also stable in CDCl_3_ solution for over a week, except for **CpCo­(Et**_**4**_ **Q)**, showing beginning of decomposition after 5 days in solution (see Figures S19–S22, Supporting Information). Notably, the **CpCo­(** ***t*** **-Bu**_**2**_ **Q)** complex exhibits better solubility in common organic solvents compared to the other CpCo­(*p*-benzoquinone) complexes prepared (see the Supporting Information, Table S-1). An attempt to synthesize the complex by using microwave heating without irradiation resulted in low yielding formation of **CpCo­(DQ)**. In addition, we tried the complexation at lower reaction temperatures (55 °C) and irradiation while bubbling argon through the reaction mixture to purge out resulting CO, thus facilitating benzoquinone coordination under less thermal harsh conditions. However, this approach also only delivered 12% yield of the product **CpCo­(Ph**_**2**_ **Q)**.

**Scheme 3:**
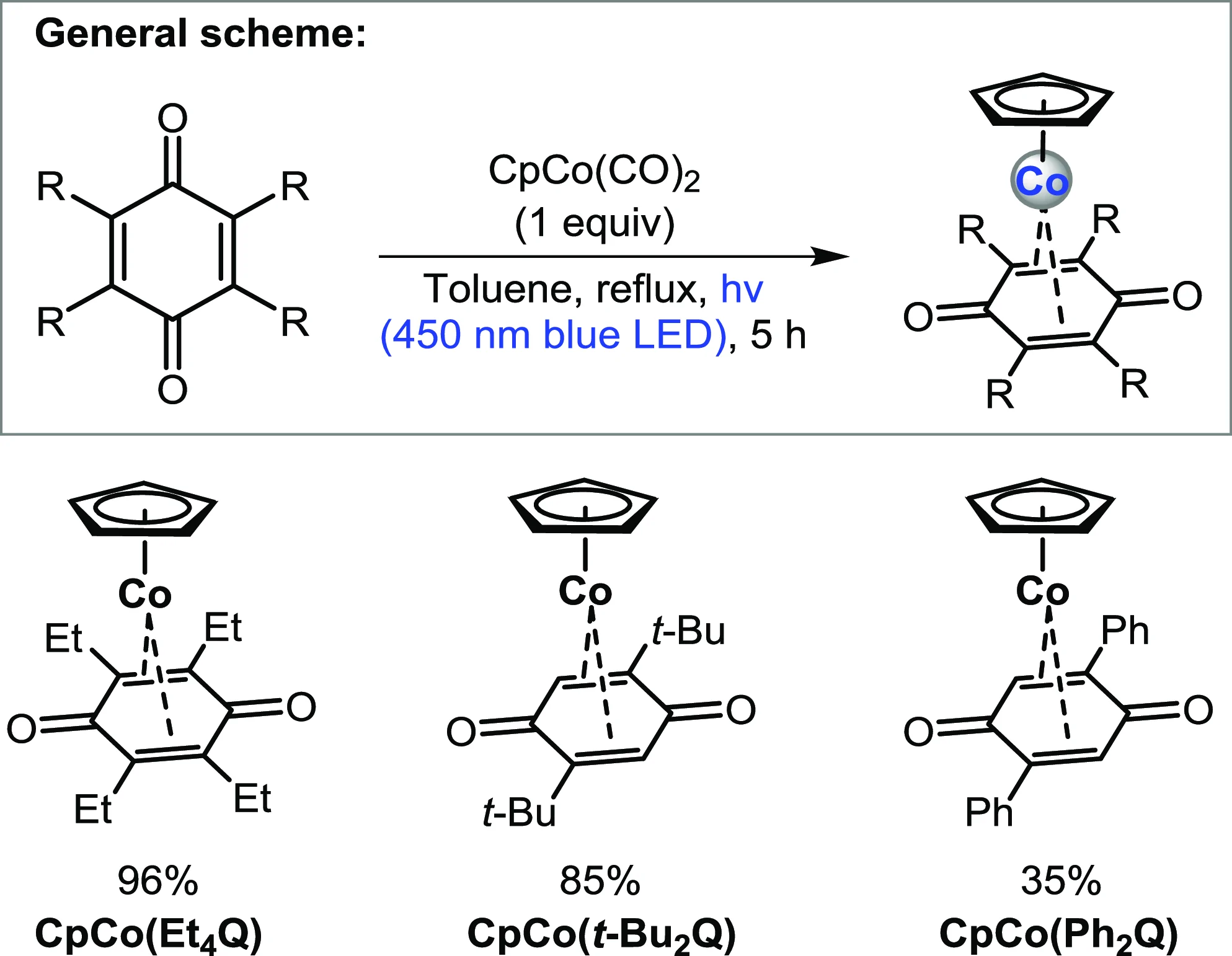
General Synthetic Procedure for CpCo­(*p*-benzoquinone) Complexes

The first structural characterization of **CpCo­(DQ)** by X-ray diffraction was reported in 1972.^14^ Over the course of this work, single crystals of all synthesized CpCo­(*p*-benzoquinone) complexes were grown and subjected to single crystal X-ray diffraction analysis (Figure 1). A notable structural feature common to all synthesized complexes is the distortion of the *p*-benzoquinone ring upon coordination to cobalt. This distortion is observed as an out-of-plane displacement of the carbonyl groups away from the metal center, while the substituents adjacent to the olefinic carbons pucker toward the metal center. Upon complexation, the single carbonyl absorption band observed in the infrared spectrum of the free *p*-benzoquinone ligand splits into two distinct carbonyl bands, as evidenced by the IR data of the complexes (see the experimental section and see Figures S26–S30, Supporting Information). This band splitting is a direct consequence of the structural distortion of the *p*-benzoquinone ring upon coordination to the metal center. These observations are in good agreement with the structural parameters reported for **CpCo­(DQ)** in the 1972 X-ray diffraction study.^14^

**Figure 1:**
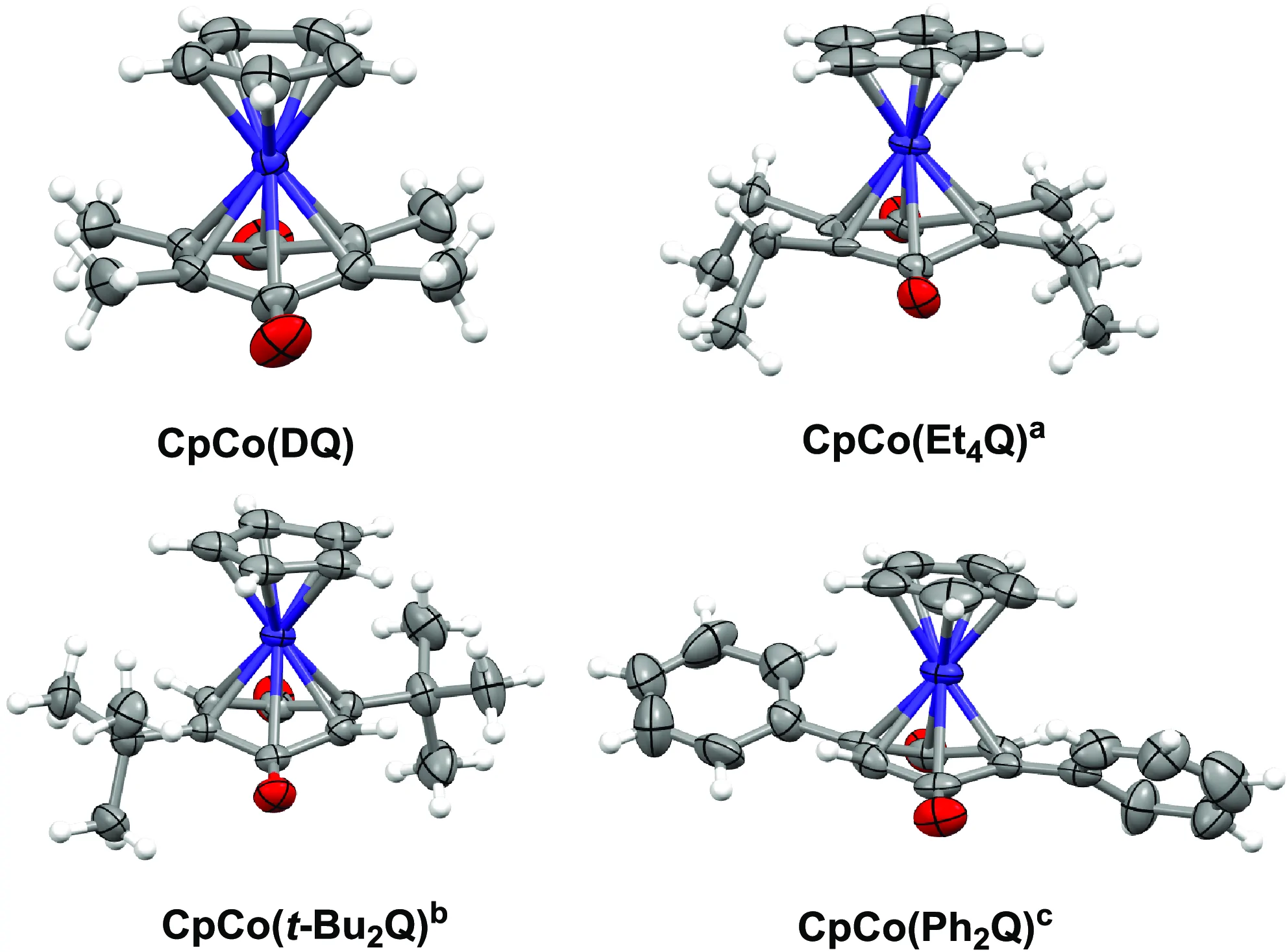
Molecular structures of benzoquinone complexes (ellipsoids drawn at the 50% probability level). ^a^Only one of the two molecules present in the asymmetric unit is shown. ^b^Only one orientation of the disordered Cp ring is shown. ^*c*^Co-crystallized ethanol omitted for clarity.

The extent of distortion in the *p*-benzoquinone ring upon coordination can be assessed by the dihedral angle between the olefinic carbon plane and the plane defined by the carbonyl carbon and its two attached carbon atoms (Table 1). Based on this dihedral angle, **CpCo­(** ***t*** **-Bu**_**2**_ **Q)** exhibits relatively more distortion than other complexes. The average C=O and C=C bond lengths of *p*-benzoquinone ligands do not differ significantly and are comparable with those reported in the literature.^18^

**Table 1:** Selected Bond Lengths and Angles

complex	C=O (Å)^a^	C=C (Å)^b^	dihedral angle (deg)
CpCo(DQ)	1.246(3)	1.409(3)	20.5(2)
CpCo(*t*-Bu_2_Q)	1.244(2)	1.418(3)	21.3(3)
CpCo(Ph_2_Q)	1.255(2)	1.418(2)	19(1)
CpCo(Et_4_Q)	1.244(9)^c^	1.421(2)	20(1)

a Average of two different C=O bonds.

b Average of two different C=C bonds.

c Average of three C=O bonds of two half molecules in the unit cell.

We next explored the catalytic activity of these complexes in cyclotrimerization reactions.^20^ As an initial benchmark study, the complexes were tested in the intermolecular reaction of diynes with nitriles toward the synthesis of pyridine derivatives (Table 2). Thermal as well as photochemical reaction conditions were screened using 1,7-octadiyne (**1a**) and benzonitrile (**2a**) as the model substrate (Table 2). When **CpCo­(DQ)** was employed as the catalyst using microwave (MW) heating at 120 °C in toluene for 1 h, only traces amount of product **3aa** were identified (Table 2, Entry 1). Notably, elevating the reaction temperature to 150 °C under the same microwave reaction conditions led to a significant improvement, affording the product **3aa** in 62% yield (Entry 2). The reaction was subsequently investigated under photochemical conditions, using 450 nm blue LED irradiation in THF at room temperature for 24 h, however, this afforded a reduced yield of 38% (Entry 3). In the next series of experiments, we applied the better soluble complex **CpCo­(** ***t*** **-Bu**_**2**_ **Q)**, which afforded product yields of 55% and 72% under microwave heating at 120 and 150 °C, respectively, after one hour reaction time in toluene (Entries 4 and 5). Notably, **CpCo­(** ***t*** **-Bu**_**2**_ **Q)** exhibited a considerably higher yield compared to **CpCo­(DQ)** at 120 °C, which could indeed be attributed to the better solubility of **CpCo­(** ***t*** **-Bu**_**2**_ **Q)** in toluene. Conventional heating using an oil bath in refluxing *o*-xylene for 3 h did not improve the yield over the microwave conditions for **CpCo­(*t*-Bu**_**2**_ **Q)** (Entry 6). The reaction of **CpCo­(** ***t*** **-Bu**_**2**_ **Q)** under irradiation at room temperature gave a lower yield of **3aa** (Entry 7). The precatalyst **CpCo­(Ph**_**2**_ **Q)** afforded diminished yields of product under both thermal and photochemical conditions (Entries 8 and 9). Remarkably, while **CpCo­(Et**_**4**_ **Q)** was ineffective under thermal conditions, it afforded a 78% yield of product upon photochemical irradiation (Entries 10 and 11). At this stage, it is not possible to definitively explain the higher yield for **CpCo­(Et**_**4**_ **Q)** under photochemical conditions. Absorption alone, at least, cannot be the reason for the higher yield, since the four compounds exhibit comparable absorption at 450 nm (see Figure S23, Supporting Information). We also performed a light-on/light-off experiment, demonstrating the necessity of continuous irradiation of the reaction mixture (see Supporting Information, Figure S-24). We further compared our CpCo­(*p*-benzoquinone) catalysts with established cobalt systems, CpCo­(CO)_2_ and CpCo­(CO)­(dmfu), under the same reaction conditions (Entries 12–15). Under thermal conditions, CpCo­(CO)_2_ was less efficient, whereas CpCo­(CO)­(dmfu) gave a comparable yield to that of **CpCo­(*t*-Bu_2_Q)** (Entries 12, 14, and 5). Under photochemical conditions, both CpCo­(CO)_2_ and CpCo­(CO)­(dmfu) were less effective compared to **CpCo­(Et_4_Q)** (Entries 13, 15, and 11).

**Table 2:**
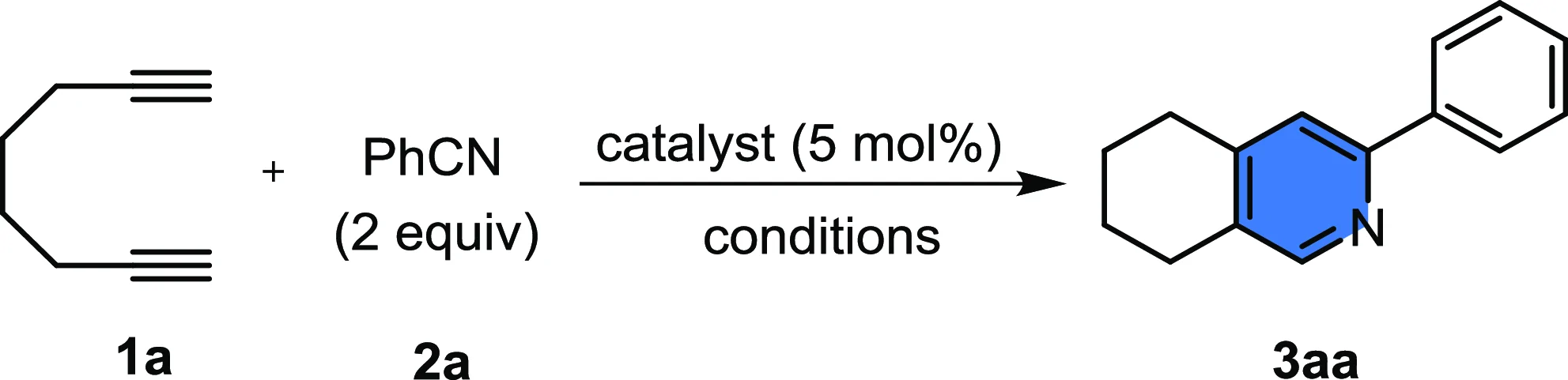
Catalysts Screening for the [2+2+2] Cycloaddition of 1,7-Octadiyne (1a) and Benzonitrile (2a)

#	catalyst	conditions	yields^a^
1	CpCo(DQ)	MW 120 °C, toluene, 1 h	traces
2	CpCo(DQ)	MW 150 °C, toluene, 1 h	62%
3	CpCo(DQ)	hν (450 nm blue LED), THF, 24 h, 26 °C	38%
4	CpCo(*t*-Bu_2_Q)	MW 120 °C, toluene, 1 h	55%
5	CpCo(*t*-Bu_2_Q)	MW 150 °C, toluene, 1 h	72%
6	CpCo(*t*-Bu_2_Q)	*o*-xylene, reflux, oil-bath, 3 h	43%
7	CpCo(*t*-Bu_2_Q)	hν (450 nm blue LED), THF, 24 h, 26 °C	26%
8	CpCo(Ph_2_Q)	MW 150 °C, toluene, 1 h	22%
9	CpCo(Ph_2_Q)	hν (450 nm blue LED), THF, 24 h, 26 °C	20%
10	CpCo(Et_4_Q)	MW 150 °C, toluene, 1 h	traces
11	CpCo(Et_4_Q)	hν (450 nm blue LED), THF, 24 h, 26 °C	78%
12	CpCo(CO)_2_	MW 150 °C, toluene, 1 h	39%
13	CpCo(CO)_2_	hν (450 nm blue LED), THF, 24 h, 26 °C	47%
14	CpCo(CO)(dmfu)	MW 150 °C, toluene, 1 h	68%
15	CpCo(CO)(dmfu)	hν (450 nm blue LED), THF, 24 h, 26 °C	52%

a Isolated yields.

The substrate scope of the pyridine formation reaction was then briefly explored using different diynes and nitriles (Scheme 4). Reaction of 1,7-octadiyne (**1a**) with cyanamides **2b** and **2c** furnished 2-aminopyridine products **3ab** and **3ac** in 70% and 58% yields, respectively. To further explore the substrate scope with different diynes, dipropargyl ether (**1b**) and 1,6-heptadiyne (**1c**) were examined in the cyclization reaction with benzonitrile (**2a**), affording the corresponding pyridine products **3ba** and **3ca** in 65% and 67% yields, respectively. Notably, tosylamine-tethered 1,6-diyne (**1d**) proved to be an excellent substrate, furnishing pyridine product **3da** in 88% yield.

**Scheme 4:**
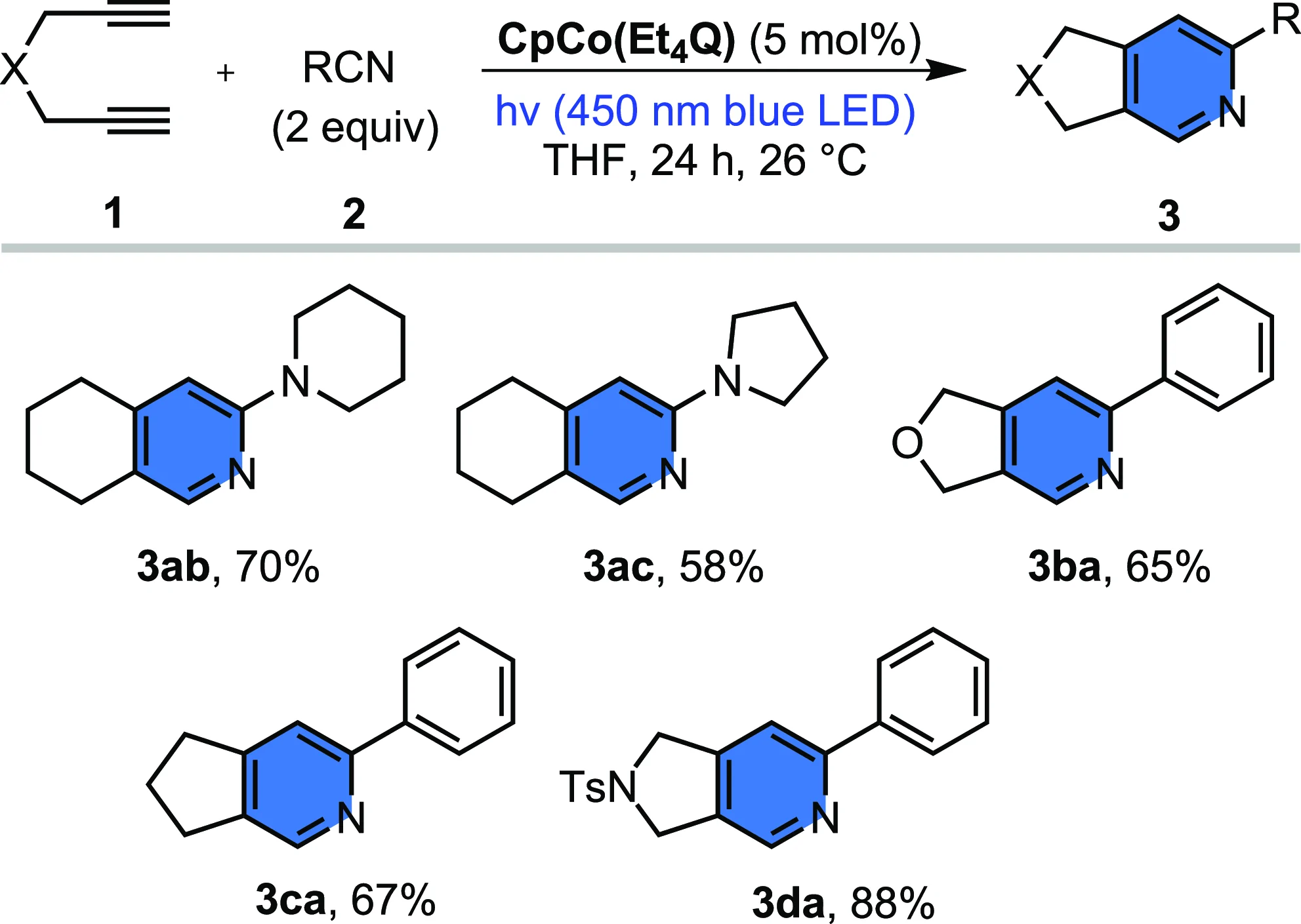
Scope of the Intermolecular [2+2+2] Cycloaddition Reaction of Different Diynes and Nitriles

We next explored the late-stage functionalization of ethisterone, a commercially available steroid (Scheme 5). Diyne **3e** was prepared from ethisterone, and its reaction with benzonitrile (**2a**) under our optimized conditions furnished the separable spirocyclic regioisomers **3ea** and **3ea'** in a combined yield of 56%, with a regioisomeric ratio of 54:46.

**Scheme 5:**
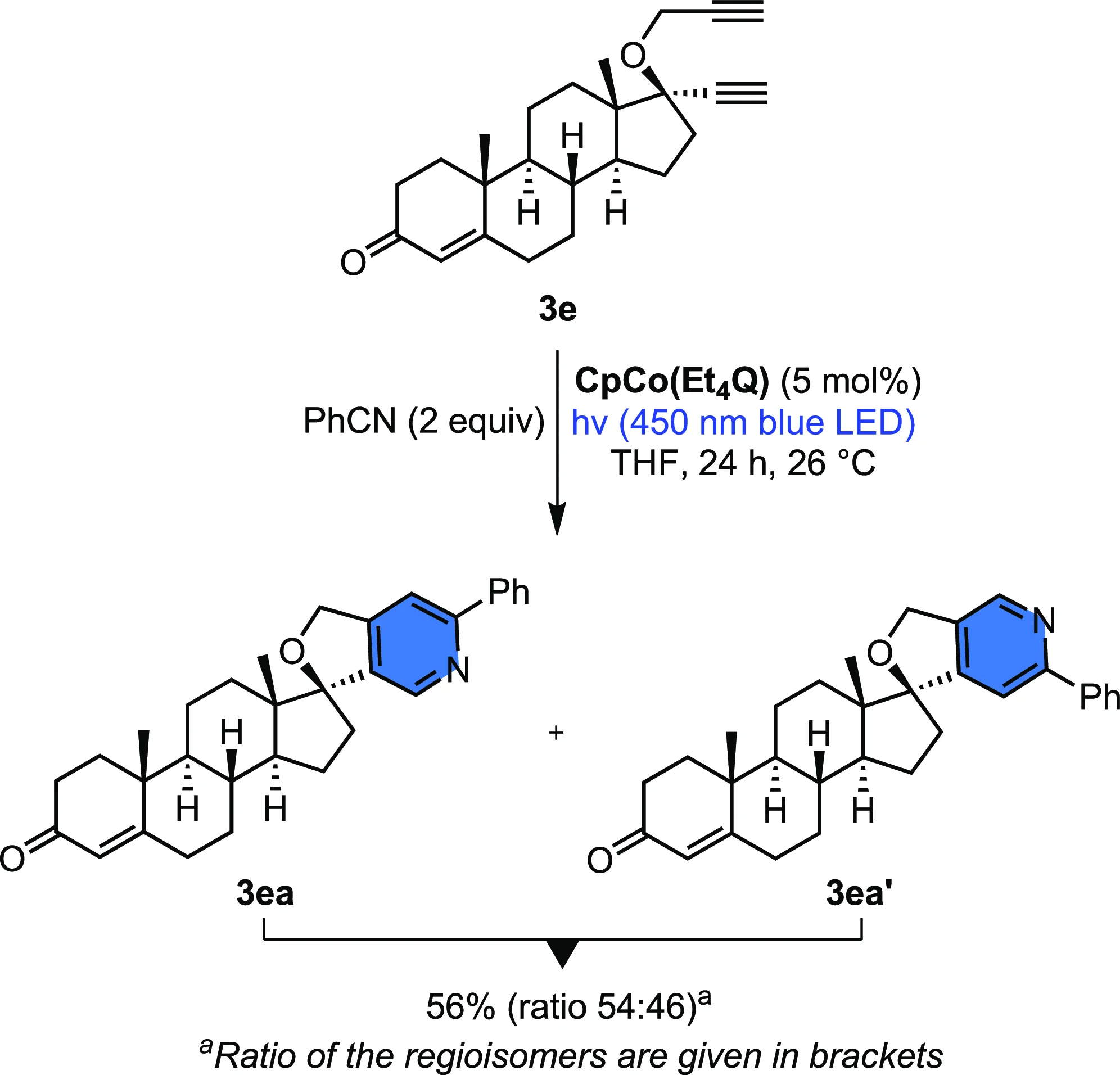
Late-Stage Functionalization of Ethisterone Derivative 3e

a Ratio of the regioisomers are given in bracket.

Finally, we examined the new precatalysts in [2+2+2] cycloaddition reactions of alkynes to form benzene derivatives. Intramolecular [2+2+2] cycloaddition of triynes **4a–c** using **CpCo­(Et**_**4**_ **Q)** under photochemical conditions furnished the corresponding cyclized products **5a–c** in moderate to good yields (61–76%) (Scheme 6 **A**). We further evaluated triynes **4a–c** under the literature-reported thermal conditions with **CpCo­(** ***t*** **-Bu**_**2**_ **Q)** (DMF, microwave heating, 200 °C, 10 min),^7^ affording cyclized products **5a–c** in moderate to good yields (52–82%). Interestingly, triyne **4d** reacted poorly under photochemical conditions to afford only 24% of cyclized product **5d**, whereas under thermal condition a full decomposition of starting material triyne **4d** was observed. Furthermore, the intermolecular [2+2+2] cycloaddition of phenylacetylene **6** under similar reaction conditions afforded the expected regioisomeric mixture of 1,3,5-triphenylbenzene (**7**) and 1,2,4-triphenylbenzene (**8**) in moderate combined yields of 55% (photochemical conditions) and 50% (thermal conditions), with an isomer ratio of 34:66 in both cases (Scheme 6 **B**).

**Scheme 6:**
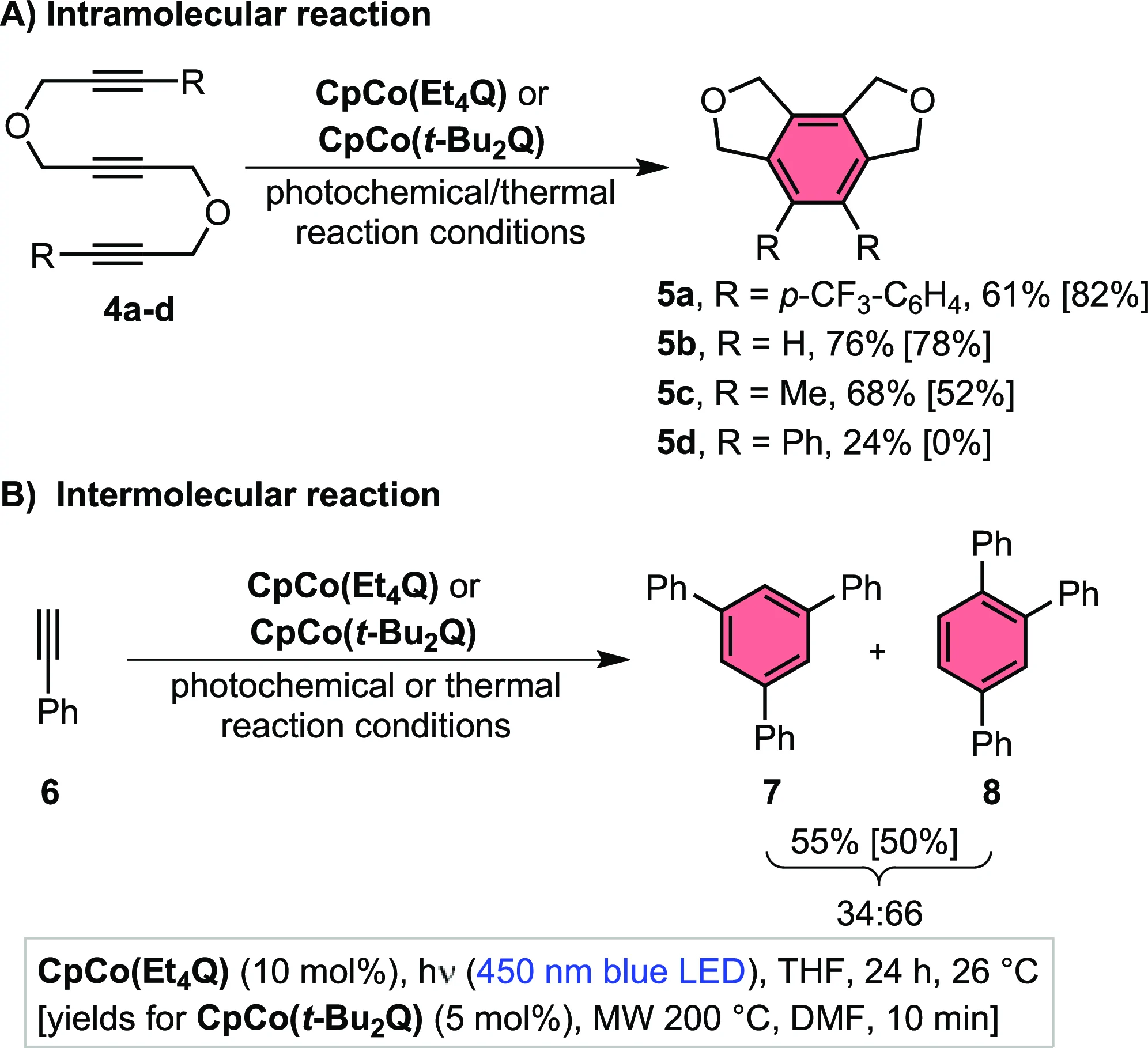
(A) Intramolecular [2+2+2] Cycloaddition Reaction of Triynes; (B) Intermolecular [2+2+2] Cycloaddition Reaction of Phenylacetylene (6)

## Conclusion

In summary, we have synthesized a series of CpCo­(*p*-benzoquinone) complexes via a convenient one-step protocol from commercially available CpCo­(CO)_2_ and quinone derivatives under photochemical conditions in generally good yields. The complexes have been structurally characterized by single crystal X-rax structure analysis. These complexes, which had not previously been explored in cyclotrimerization reactions, were found to catalyze [2+2+2] cycloadditions under thermal and photochemical conditions, enabling the formation of pyridine and benzene derivatives. Their performance as precatalyst appeared to be influenced by the nature of the quinone derivative under both thermal and photochemical conditions.

## Experimental Section

### General Information

All experiments were carried out under an inert gas atmosphere in flame-dried Schlenk flask or microwave vial using standard Schlenk-line techniques or in an argon-filled glovebox, unless otherwise stated. All microwave experiments were carried out in original Biotage 2–5 mL microwave vials with a Biotage Initator+ Microwave System with Robot Eight. All photochemical cyclization reactions were conducted in an air-cooled EvoluChem PhotoRedOx Box equipped with a 30 W LED emitting at 450 nm. Solvents (tetrahydrofuran and toluene) were dried using an MB SPS7 solvent purification system from MBraun and degassed by three freeze–pump–thaw cycles. DMF was used directly from the bottle without any further distillation or degassing. All other chemicals (Aldrich, BLDPharm, Fluorochem, Merck and TCI) were purchased and used as received.

Thin layer chromatography was performed on Merck silica gel 60 F_254_ precoated aluminum sheets. Flash column chromatography was performed on silica gel obtained from Macherey-Nagel (particle size: 0.04–0.063 mm) with an Interchim puriFlash XS420 chromatograph equipped with an UV-detector.

NMR spectroscopy was performed on a Bruker Avance 300 MHz spectrometer (^1^H: 300 MHz and ^13^C: 75 MHz) or a Bruker Avance III 500 MHz spectrometer (^1^H: 500 MHz and ^13^C: 126 MHz) at 298 K, Chemical shifts are given in parts per million (ppm) on a delta scale (δ). Axis calibration was performed using the residual proton signals of deuterated solvent (^1^H NMR: CDCl_3_: 7.26 ppm, ^13^C NMR: CDCl_3_: 77.16 ppm). The following abbreviations were used to define the signal multiplicity: s (singlet), brs (broad singlet), d (doublet), t (triplet), q (quartet), quint (quintet) and m (multiplet). High-resolution mass spectrometry was carried out on an Agilent QTOF 6520 with electrospray ionization (ESI) (mass error: ± 5 ppm) and Thermo Fisher Orbitrap Exploris GC-MS in EI ionization mode at 70 eV. The IR samples were measured neat on Bruker α II platinum-ATR instrument and are reported in wave numbers (cm^–1^). UV–vis absorption spectra were obtained from Shimadzu UV-1800 instrument. Melting points were determined with the Büchi Melting Point M-560.

Diyne **3d** and triynes **4a-d** was prepared according to the reported literature procedure.^21^ Diyne **3e** was also prepared according to the reported literature procedure.^22^ **Et**_**4**_ **Q** was prepared using a modified literature procedure (see Supporting Information).^19^

**CAUTION:** The complexation reactions generate carbon monoxide (CO), which is highly toxic. Conduct the experiments in a properly functioning fume hood with the system fully contained and a sufficiently sized gas vent to prevent pressure buildup. Ideally, use carbon monoxide detectors to warn personnel of any gas leakage.

#### General synthetic procedure (GSP) of CpCo­(*p*-benzoquinone) complexes

In a Schlenk flask inside of an argon-filled glovebox, the respective *p*-benzoquinone (1.0 mmol, 1.0 equiv) and CpCo­(CO)_2_ (180 mg, 1.0 mmol, 1.0 equiv) were dissolved in 10 mL toluene. The flask was then taken out of the glovebox and refluxed under irradiation with 450 nm blue LED light for 5 h, while connected to a Schlenk line equipped with Stutz valve to release the evolving CO during the reaction (see the Supporting Information, Figure S1 for the full reaction setup). The crude reaction mixture was purified via flash column chromatography on silica gel using eluent composition DCM/MeOH (9:1, v/v) to obtain the corresponding CpCo­(*p*-benzoquinone) complexes. The characterization data of complexes are given below.

##### **CpCo­(DQ)** ^9^

The compound was synthesized according to the GSP, starting from duroquinone (**DQ**, 164 mg, 1.0 mmol, 1.0 equiv) and CpCo­(CO)_2_ (180 mg, 1.0 mmol, 1.0 equiv) and was obtained as dark red solid. Yield: 92% (265 mg, 0.92 mmol). Mp = 260 °C (decomp).

R_f_ (DCM/MeOH, 9:1 v/v) = 0.23. ^1^H NMR (300 MHz, CDCl_3_): δ = 4.74 (s, 5H), 2.08 (s, 12H). ^13^C­{^1^H} NMR (75 MHz, CDCl_3_): δ = 157.7, 91.6, 85.7, 14.6. IR (neat, cm^–1^): 3089, 1567, 1524, 1476, 1434, 1414, 1376, 1124, 1027, 852, 728. HRMS (ESI): *m*/*z* calcd. for C_15_H_18_CoO_2_ [M+H]^+^: 289.0633, found: 289.0633.

##### **CpCo­(*t*-Bu_2_Q)** ^15^

The compound was synthesized according to the GSP, starting from 2,5-di­(*tert*-butyl)-1,4-benzoquinone (***t*** **-Bu**_**2**_ **Q**, 220 mg, 1.0 mmol, 1.0 equiv) and CpCo­(CO)_2_ (180 mg, 1.0 mmol, 1.0 equiv) and obtained as dark red solid. Yield: 85% (293 mg, 0.85 mmol). Mp = 192 °C (decomp).

R_f_ (DCM/MeOH, 9:1 v/v) = 0.65. ^1^H NMR (300 MHz, CDCl_3_): δ = 5.30 (brs, 2H), 5.22 (s, 5H), 1.35 (s, 18H). ^13^C­{^1^H} NMR (126 MHz, CDCl_3_): δ = 160.0, 107.9, 83.0, 75.1, 34.7, 29.1. IR (neat, cm^–1^): 3094, 2955, 2909, 2869, 1612, 1585, 1480, 1443, 1405, 1386, 1363, 1245, 1089, 845, 820, 732, 681. HRMS (ESI): *m*/*z* calcd. for C_19_H_26_CoO_2_ [M+H]^+^: 345.1259, found: 345.1259.

##### CpCo­(Et_4_Q)

The compound was obtained according to the GSP, starting from 2,3,5,6-tetraethyl-1,4-benzoquinone (**Et**_**4**_ **Q**, 220 mg, 1.0 mmol, 1.0 equiv) and CpCo­(CO)_2_ (180 mg, 1.0 mmol, 1.0 equiv) and isolated as dark red solid. Yield: 96% (331 mg, 0.96 mmol). Mp = 230 °C (decomp).

R_f_ (DCM/MeOH, 9:1 v/v) = 0.44. ^1^H NMR (300 MHz, CDCl_3_): δ = 4.71 (s, 5H), 2.96 (q, *J* = 6.4 Hz, 4H), 2.14 (q, *J* = 6.4 Hz, 4H), 1.28 (t, *J* = 7.0 Hz, 12H). ^13^C­{^1^H} NMR (75 MHz, CDCl_3_): δ = 157.7, 96.8, 85.0, 23.2, 14.3. IR (neat, cm^–1^): 2967, 2937, 2876, 1581, 1558, 1449, 1428, 1399, 1362, 1336, 1252, 1130, 1056, 902, 838, 823, 708, 653. HRMS (ESI): *m*/*z* calcd. for C_19_H_26_CoO_2_ [M+H]^+^: 345.1259, found: 345.1259.

##### CpCo­(Ph_2_Q)

The compound was synthesized according to the GSP, starting from 2,5-diphenyl-1,4-benzoquinone (**Ph**_**2**_ **Q**, 260 mg, 1.0 mmol, 1.0 equiv) and CpCo­(CO)_2_ (180 mg, 1.0 mmol, 1.0 equiv) and isolated as dark red solid. Yield: 35% (135 mg, 0.35 mmol). Mp = 190 °C (decomp).

R_f_ (9/1 DCM/MeOH) = 0.63. ^1^H NMR (300 MHz, CDCl_3_): δ = 7.91–7.95 (m, 4H), 7.42–7.44 (m, 6H), 6.05 (s, 2H), 4.93 (s, 5H). ^13^C­{^1^H} NMR (126 MHz, CDCl_3_): δ = 159.4, 133.6, 129.9, 129.5, 128.9, 95.9, 86.6, 76.0 ppm. IR (neat, cm^–1^): 3085, 3054, 3028, 1586, 1574, 1495, 1446, 1401, 1241, 1177, 1010, 818, 762, 724, 687, 619. HRMS (ESI): *m*/*z* calcd. for C_23_H_18_CoO_2_ [M+H]^+^: 385.0633, found: 385.0633.

#### General Procedure for the Synthesis of Pyridines under Photochemical Conditions

A 5 mL microwave vial was charged with respective diyne (0.5 mmol, 1.0 equiv), nitrile (1.0 mmol, 2.0 equiv) and **CpCo­(Et**_**4**_ **Q)** (8.6 mg, 5 mol%) in 5 mL THF. The reaction mixture was irradiated at room temperature under 450 nm blue LED light for 24 h. The volatiles were removed under reduced pressure and the crude product was purified by flash column chromatography on silica gel using *n*-heptane/EtOAc as the eluent. The characterization data of pyridines are given below.

##### 3-Phenyl-5,6,7,8-tetrahydroisoquinoline (**3aa**)^7^

Colorless oil. Yield: 78% (82 mg). Eluent composition (*n*-heptane/EtOAc, 19:1 v/v). ^1^H NMR (300 MHz, CDCl_3_): δ = 8.38 (s, 1H), 7.93–7.96 (m, 2H), 7.34–7.48 (m, 4H), 2.76–2.82 (m, 4H), 1.82–1.86 (m, 4H). ^13^C­{^1^H} NMR (75 MHz, CDCl_3_): δ = 154.5, 150.4, 146.8, 139.8, 131.8, 128.7, 128.4, 126.8, 120.8, 29.0, 26.1, 22.8, 22.6. HRMS (ESI): *m*/*z* calcd. for C_15_H_16_N [M+H]^+^: 210.1277, found: 210.1283.

##### 3-(Piperidin-1-yl)-5,6,7,8-tetrahydroisoquinoline (**3ab**)

Colorless oil. Yield: 70% (76 mg). Eluent composition (*n*-heptane/EtOAc, 19:1 v/v). ^1^H NMR (300 MHz, CDCl_3_): δ = 7.91 (s, 1H), 6.36 (s, 1H), 3.41–3.43 (m, 4H), 2.60–2.68 (m, 4H), 1.73–1.77 (m, 4H), 1.62–1.65 (m, 6H). ^13^C­{^1^H} NMR (75 MHz, CDCl_3_): δ = 158.7, 148.0, 147.6, 122.4, 106.9, 47.1, 29.5, 25.7, 25.6, 24.9, 23.4, 22.9. GC-HRMS (EI): *m*/*z* calcd. for C_14_H_20_N_2_ [M]^+^: 216.1621, found: 216.1620.

##### 3-(Pyrrolidin-1-yl)-5,6,7,8-tetrahydroisoquinoline (**3ac**)^23^

White solid. Yield: 58% (59 mg). Eluent composition (*n*-heptane/EtOAc, 9:1 v/v). ^1^H NMR (300 MHz, CDCl_3_): δ = 7.87 (s, 1H), 6.06 (s, 1H), 3.37–3.42 (m, 4H), 2.59–2.68 (m, 4H), 1.94–1.99 (m, 4H), 1.72–1.76 (m, 4H). ^13^C­{^1^H} NMR (75 MHz, CDCl_3_): δ = 156.2, 148.1, 147.3, 120.6, 105.5, 46.8, 29.4, 25.7, 25.6, 23.5, 23.0. GC-HRMS (EI): *m*/*z* calcd. for C_13_H_18_N_2_ [M]^+^: 202.1465, found: 202.1464.

##### 6-Phenyl-1,3-dihydrofuro­[3,4-*c*]­pyridine (**3ba**)

White solid. Yield: 65% (64 mg). Eluent composition (*n*-heptane/EtOAc, 4:1 v/v). ^1^H NMR (300 MHz, CDCl_3_): δ = 8.59 (s, 1H), 7.95–7.98 (m, 2H), 7.60 (s, 1H), 7.38–7.50 (m, 3H), 5.13–5.17 (m, 4H). ^13^C­{^1^H} NMR (75 MHz, CDCl_3_): δ = 156.7, 150.0, 142.6, 139.4, 133.9, 129.1, 128.9, 127.1, 113.3, 73.0, 71.7. HRMS (ESI): *m*/*z* calcd. for C_13_H_12_NO [M+H]^+^: 198.0913, found: 198.0912.

##### 3-Phenyl-6,7-dihydro-5H-cyclopenta­[*c*]­pyridine (**3ca**)^8c^

White solid. Yield: 67% (67 mg). Eluent composition (*n*-heptane/EtOAc, 9:1 v/v). ^1^H NMR (300 MHz, CDCl_3_): δ = 8.54 (s, 1H), 7.93–7.97 (m, 2H), 7.60 (s, 1H), 7.35–7.48 (m, 3H), 2.96 (t, *J* = 7.5 Hz, 4H), 2.13 (quint, *J* = 7.5 Hz, 2H) ^13^C­{^1^H} NMR (75 MHz, CDCl_3_): δ = 155.5, 154.7, 145.5, 140.1, 138.8, 128.7, 128.5, 127.0, 116.9, 32.8, 30.1, 25.2. HRMS (ESI): *m*/*z* calcd. for C_14_H_14_N [M+H]^+^: 196.1121, found: 196.1124.

##### 6-Phenyl-2-tosyl-2,3-dihydro-1H-pyrrolo­[3,4-*c*]­pyridine (**3da**)^7^

White solid. Yield: 88% (154 mg). Eluent composition (*n*-heptane/EtOAc, 1:1 v/v). ^1^H NMR (300 MHz, CDCl_3_): δ = 8.52 (s, 1H), 7.89–7.92 (m, 2H), 7.78 (d, *J* = 8.3 Hz, 2H), 7.54 (s, 1H), 7.40–7.48 (m, 3H), 7.33 (d, *J* = 8.0 Hz, 2H), 4.66–4.68 (m, 4H), 2.40 (s, 3H). ^13^C­{^1^H} NMR (75 MHz, CDCl_3_): δ = 157.2, 146.7, 144.2, 144.1, 139.0, 133.6, 131.1, 130.1, 129.3, 128.9, 127.7, 127.0, 114.6, 53.5, 51.8, 21.6. HRMS (ESI): *m*/*z* calcd. for C_20_H_19_N_2_O_2_S [M+H]^+^: 351.1162, found: 351.1164.

#### [2+2+2] Cycloaddition of Diyne Substrate 3e Using the General Protocol

##### Product 3ea

White solid. Yield: 30% (68 mg). Eluent composition (*n*-heptane/EtOAc, 1:4 v/v). ^1^H NMR (300 MHz, CDCl_3_): δ = 8.54 (s, 1H), 7.97–8.01 (m, 2H), 7.59 (s, 1H), 7.37–7.49 (m, 3H), 5.73 (s, 1H), 5.01 (s, 2H), 2.24–2.47 (m, 5H), 2.03–2.13 (m, 1H), 1.84–1.99 (m, 3H), 1.53–1.73 (m, 4H), 1.25–1.42 (m, 3H), 1.17 (s, 3H), 1.04–1.14 (m, 4H), 0.67–0.89 (m, 2H). ^13^C­{^1^H} NMR (75 MHz, CDCl_3_): δ = 199.5, 170.9, 156.3, 150.7, 143.4, 140.0, 139.0, 129.2, 128.9, 127.0, 124.1, 112.9, 97.9, 71.2, 53.3, 49.7, 47.7, 38.7, 36.5, 36.2, 35.7, 34.0, 33.2, 32.8, 31.8, 23.6, 20.7, 17.5, 15.2. HRMS (ESI): *m*/*z* calcd. for C_31_H_36_NO_2_ [M+H]^+^: 454.2741, found: 454.2746.

##### Product 3ea’

White solid. Yield: 26% (59 mg). Eluent composition (*n*-heptane/EtOAc, 1:3 v/v). ^1^H NMR (300 MHz, CDCl_3_): δ = 8.55 (s, 1H), 7.94–7.97 (m, 2H), 7.40–7.52 (m, 4H), 5.72 (s, 1H), 5.03 (s, 2H), 2.26–2.43 (m, 5H), 2.03–2.16 (m, 1H), 1.85–2.00 (m, 3H), 1.51–1.72 (m, 4H), 1.29–1.43 (m, 3H), 1.06–1.22 (m, 7H), 0.64–0.90 (m, 2H). ^13^C­{^1^H} NMR (75 MHz, CDCl_3_): δ = 199.4, 170.7, 156.9, 155.7, 142.5, 139.6, 134.6, 129.1, 128.9, 127.2, 124.1, 114.5, 98.6, 69.8, 53.4, 50.4, 47.9, 38.7, 36.3, 36.2, 35.6, 34.0, 33.4, 32.8, 31.8, 23.9, 20.6, 17.5, 15.4. HRMS (ESI): *m*/*z* calcd. for C_31_H_36_NO_2_ [M+H]^+^: 454.2741, found: 454.2749.

#### General Procedure for the Synthesis of Benzene Derivatives under Photochemical Conditions

A 5 mL microwave vial was charged with triyne **4a-c** (0.4 mmol, 1.0 equiv) or phenylacetylene **6** (102 mg, 1.0 mmol) and **CpCo­(Et**_**4**_ **Q)** (10 mol%) in 2 mL THF. The reaction mixture was irradiated at room temperature under 450 nm blue LED light for 24 h. The volatiles were removed under reduced pressure and the crude product was purified by flash column chromatography on silica gel using *n*-heptane/EtOAc as the eluent.

#### General Procedure for the Synthesis of Benzene Derivatives under Thermal Conditions

A 5 mL microwave vial was charged with triyne **4a-c** (0.4 mmol, 1.0 equiv) or phenylacetylene **6** (102 mg, 1.0 mmol) and **CpCo­(** ***t*** **-Bu**_**2**_ **Q)** (5 mol%) in 2 mL DMF. The reaction mixture was stirred in a microwave for 10 min at 200 °C. The volatiles were removed under reduced pressure and the crude product was purified by flash column chromatography on silica gel using *n*-heptane/EtOAc as the eluent.

**The Characterization Data of **5a-d**, **7** and **8** Are Given Below**

#### 4,5-Bis­(4-(trifluoromethyl)­phenyl)-1,3,6,8-tetrahydrobenzo­[1,2-c:3,4-c’]­difuran (**5a**)^7^

White solid. Photochemical reaction yield: 61% (110 mg) and thermal reaction yield: 82% (148 mg). Eluent composition (*n*-heptane/EtOAc, 4:1 v/v). ^1^H NMR (300 MHz, CDCl_3_): δ = 7.47–7.50 (m, 4H), 7.13–7.16 (m, 4H), 5.15 (s, 4H), 4.96 (s, 4H). ^13^C­{^1^H} NMR (75 MHz, CDCl_3_): δ = 141.9, 139.1, 132.8, 132.6, 129.9, 129.6 (q, *J* = 32.6 Hz), 125.4 (q, *J* = 3.7 Hz), 124.0 (q, *J* = 272.2 Hz), 73.5, 72.8. ^19^F­{^1^H} NMR (471 MHz, CDCl_3_): δ = −62.67. GC-HRMS (EI): *m*/*z* calcd. for C_24_H_16_F_6_O_2_ [M] ^+^: 450.1049, found:450.1044.

#### 1,3,6,8-Tetrahydrobenzo­[1,2-c:3,4-c’]­difuran (**5b**)^7^

White solid. Photochemical reaction yield: 76% (49 mg) and thermal reaction yield: 78% (51 mg). Eluent composition (*n*-heptane/EtOAc, 4:1 v/v). ^1^H NMR (300 MHz, CDCl_3_): δ = 7.14 (s, 2H), 5.12 (s, 4H), 5.03 (s, 4H). ^13^C­{^1^H} NMR (75 MHz, CDCl_3_): δ = 138.8, 132.4, 120.0, 73.5, 72.3. HRMS (ESI): *m*/*z* calcd. for C_10_H_11_O_2_ [M+H]^+^: 163.0754, found: 163.0758.

#### 4,5-Dimethyl-1,3,6,8-tetrahydrobenzo­[1,2-c:3,4-c’]­difuran (**5c**)^7^

White solid. Photochemical reaction yield: 68% (52 mg) and thermal reaction yield: 52% (40 mg). Eluent composition (*n*-heptane/EtOAc, 3:1 v/v). ^1^H NMR (300 MHz, CDCl_3_): δ = 5.08 (s, 4H), 5.03 (s, 4H), 2.15 (s, 6H). ^13^C­{^1^H} NMR (75 MHz, CDCl_3_): δ = 138.3, 129.0, 128.7, 73.5, 73.0, 15.6. GC-HRMS (EI): *m*/*z* calcd. for C_12_H_14_O_2_ [M]^+^: 190.0988, found: 190.0989.

#### 4,5-Diphenyl-1,3,6,8-tetrahydrobenzo­[1,2-c:3,4-c’]­difuran (**5d**)^7^

White solid. Photochemical reaction yield: 24% (30 mg). Eluent composition (*n*-heptane/EtOAc, 9:1 v/v). ^1^H NMR (300 MHz, CDCl_3_): δ = 7.15–7.22 (m, 6H), 7.00–7.04 (m, 4H), 5.15 (s, 4H), 4.99 (s, 4H). ^13^C­{^1^H} NMR (75 MHz, CDCl_3_): δ = 138.9, 138.6, 134.4, 131.5, 129.6, 128.1, 127.0, 73.8, 72.9. GC-HRMS (EI): *m*/*z* calcd. for C_22_H_18_O_2_ [M]^+^: 314.1301, found: 314.1300.

#### 1,3,5-Triphenylbenzene (7) and 1,2,4-triphenylbenzene (8) (isomer mixture of 34:66 7:8)^7^

Yellow oil. Photochemical reaction yield: 55% (56 mg) and thermal reaction yield: 50% (51 mg). Eluent composition (*n*-heptane/EtOAc, 99:1 v/v). ^1^H NMR (300 MHz, CDCl_3_): δ = 7.86 (s, 3H), 7.70–7.78 (m, 14H), 7.42–7.59 (m, 18H), 7.25–7.30 (m, 19H). ^13^C­{^1^H} NMR (75 MHz, CDCl_3_): δ = 142.5, 141.6, 141.3, 141.2, 141.1, 140.7, 140.5, 139.7, 131.2, 130.0, 130.0, 129.5, 129.0, 128.9, 128.0, 128.0, 127.7, 127.5, 127.5, 127.3, 126.7, 126.6, 126.2, 125.3.

## Supplementary Material



## Notes

Detailed experimental information on the synthesis of starting materials, ligands, products, and spectral data for all synthesized compounds, as well as crystalographic details for **CpCo­(DQ)**, **CpCo­(Et_4_Q)**, **CpCo­(*t*-Bu_2_Q)**, and **CpCo­(Ph_2_Q)** (PDF)
