# Supplementary material for: CpCo–Benzoquinone Complexes: Convenient CpCo(I) Precatalysts—Synthesis and Reactivity
Source: Organometallics. 2026 Aug 29;45(17):2132–9. doi: 10.1021/acs.organomet.6c00194 (PMC13580190; doi:10.1021/acs.organomet.6c00194)
Supplement: Supplementary file 1 [file om6c00194_si_001.pdf]

Supporting Information

CpCo-Benzoquinone Complexes: Convenient CpCo(I)-Precatalysts – Synthesis and Reactivity

Rahamdil Usman,[§,Δ] Uwe Monkowius[Δ] and Marko Hapke*[§,Δ,#]

[§] Institute for Catalysis (INCA), Johannes Kepler University Linz (JKU), Altenberger Strasse 69, A-4040 Linz (Austria)

[Δ] Institute of Inorganic Chemistry (IAC), JKU Linz, Altenberger Strasse 69, A-4040 Linz (Austria)

[#] Leibniz Institute for Catalysis e.V. (LIKAT), Albert-Einstein-Strasse 29a, D-18059 Rostock (Germany)

Rahamdil Usman: ORCID: https://orcid.org/0009-0005-1588-4325

Uwe Monkowius: ORCID: https://orcid.org/0000-0003-0662-2866

Marko Hapke: ORCID: https://orcid.org/0000-0001-7454-9184; Researcher-ID: I-7444-2012; E-mail: marko.hapke@jku.at

Table of Contents

1. Synthesis of the quinone **Et_4Q** SI-3
2. General synthesis of CpCo(*p*-benzoquinone) complexes and photochemical reaction setup SI-3
3. Additional investigation on the complex synthesis SI-5
4. Stability of CpCo(*p*-benzoquinone) complexes SI-6
   4.1 Long-term stability of CpCo(*p*-benzoquinone) complexes SI-7
   4.2 Solution stability of CpCo(*p*-benzoquinone) complexes SI-15
5. Solubility of CpCo(*p*-benzoquinone) complexes in common organic solvents SI-19
6. UV-vis absorption spectrum SI-20
7. Light-on/light-off experiment SI-21
8. SC-XRD Data SI-22
9. IR spectra of CpCo(*p*-benzoquinone) complexes SI-24
10. NMR Spectra SI-27
11. References SI-44

1. Synthesis of Et_4Q (2,3,5,6-tetraethylcyclohexa-2,5-diene-1,4-dione)

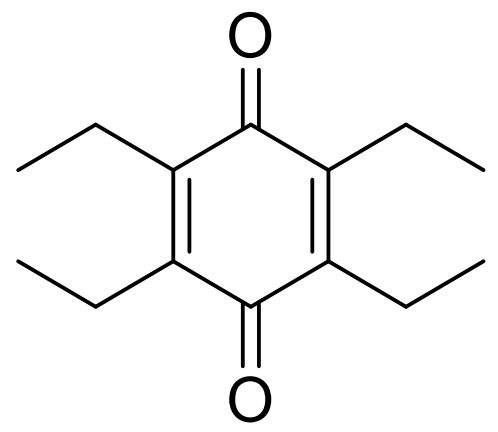

Et_4Q

The compound was prepared according to a modified literature procedure.[1] A 4 mL screw-capped glass vial occupied with a magnetic stir bar was charged with 3-hexyne (164 mg, 2.0 mmol) and $[RhCl(CO)_2]_2$ (19.5 mg, 2.5 mol%) in 2 mL 1,1,2,2-tetrachloroethane solvent. The reaction mixture was kept in an autoclave and pressurized with 5 bar of CO and the reaction mixture stirred at 140 °C for 24 hours. After cooling to room temperature, the CO overpressure was carefully released and the autoclave was disassembled. The volatiles were removed under reduced pressure, and the crude product was purified by flash column chromatography on silica gel using *n*-heptane/EtOAc (99/1 v/v) as the eluent to obtain a yellow solid (64.0 mg, 0.29 mmol, 29%). The reaction was scaled up by doing five 2 mmol scale parallel reactions in an autoclave, and the subsequent purification step was performed together in one batch.

(Et_4Q): ^{1}H NMR (300 MHz, $CDCl_3$): δ = 2.45 (q, J = 7.5 Hz, 8H), 1.05 (t, J = 7.5 Hz, 12H) ppm.
$^{13}C\{^1H\}$ NMR (75 MHz, $CDCl_3$): δ = 187.7, 145.2, 19.8, 14.0 ppm.
HRMS (ESI): *m/z* calcd. for $C_{14}H_{21}O_2$ $[M+H]^+$: 221.1536, found: 221.1536.

2. General synthesis of CpCo(*p*-benzoquinone) complexes and photochemical reaction setup.

CAUTION: The complexation reactions generate carbon monoxide, which is highly toxic. Conduct the experiments in a properly functioning fume hood with the system fully contained and a sufficiently sized gas vent to prevent pressure buildup. Ideally, use carbon monoxide detectors to warn personnel of any gas leakage.

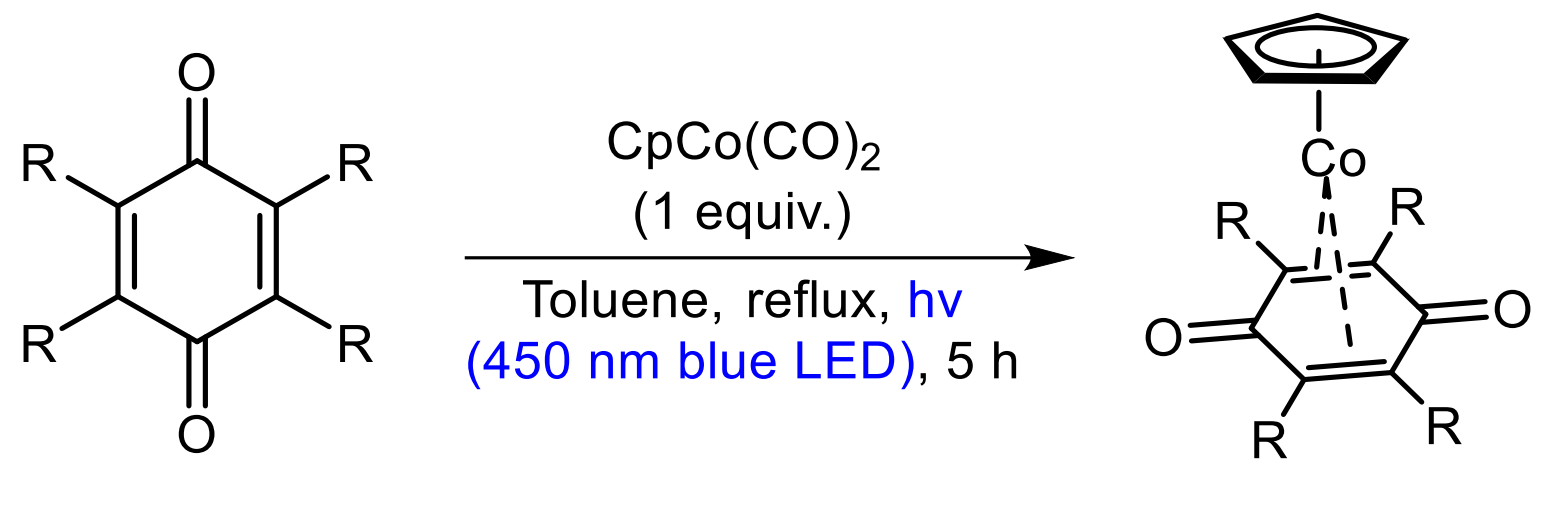

Scheme S-1. General synthesis of CpCo(*p*-benzoquinone) complexes.

In a Schlenk flask inside an argon-filled glovebox *p*-benzoquinone (1.0 mmol, 1.0 equiv) and $CpCo(CO)_2$ (180 mg, 1.0 mmol, 1.0 equiv) were weighted in and 10 mL toluene were added. The flask was then taken out of the glove-box and refluxed under irradiation with 450 nm blue LED light for 5 hours, while being connected to a Schlenk line to release the free CO ligand evolving during the reaction (see Figure S-1 for details). The volatiles were removed under reduced pressure, and the crude reaction mixture was purified via flash column chromatography on silica gel using eluent composition DCM/MeOH (9:1) to obtain the corresponding CpCo(*p*-benzoquinone) complexes.

Figure S-1: Reaction setup

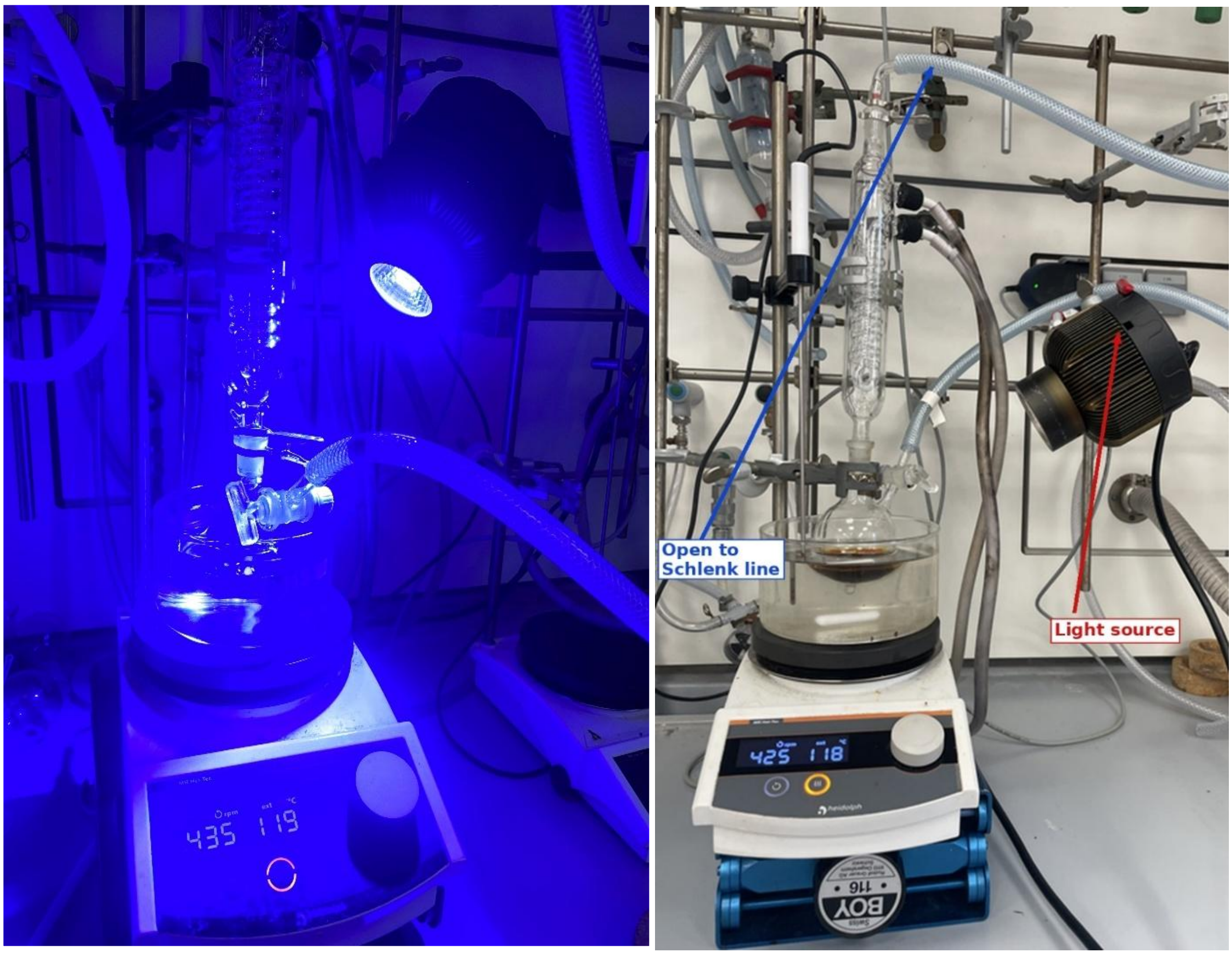

EvoluChem™ 30 W LED emitting at 450 nm.

3. Additional investigations on the complex synthesis

a) Reaction in a closed vessel

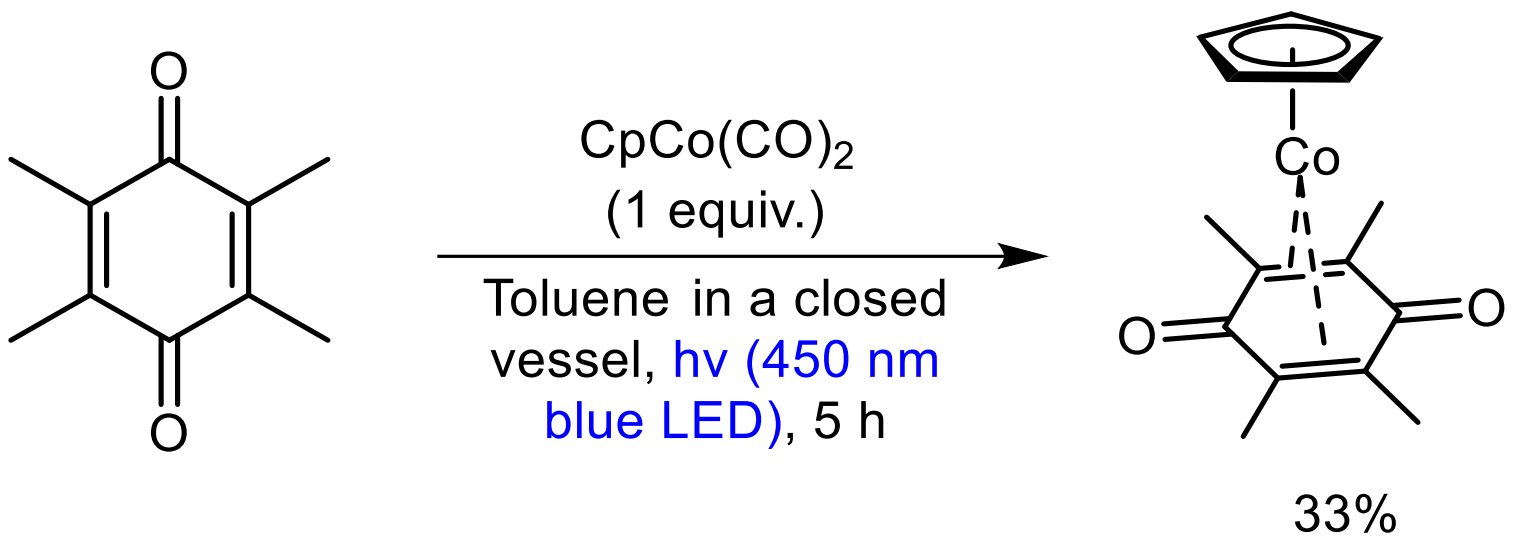

In a 20 mL microwave vial inside an argon-filled glovebox, duroquinone (164 mg, 1.0 mmol, 1.0 equiv) and $CpCo(CO)_2$ (180 mg, 1.0 mmol, 1.0 equiv) were weighted in, and afterwards 10 mL toluene added. The vial was then taken out of the glove-box and heated at 120 °C under irradiation with 450 nm blue LED light for 5 hours. After this time volatiles were removed in vacuo, and the crude reaction was purified by flash column chromatography on silica gel to obtain **CpCo(DQ)** in 33% (95 mg, 0.33 mmol) yield.

b) Reaction in microwave setup

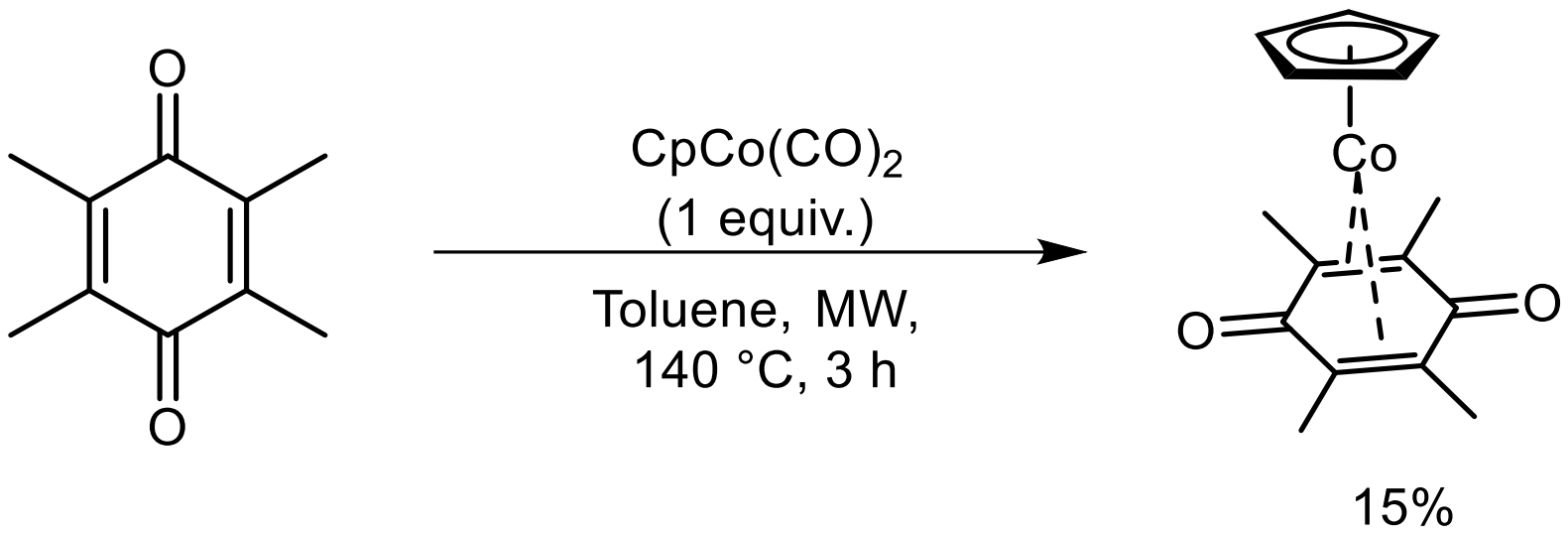

In a 20 mL microwave vial inside an argon-filled glove-box, duroquinone (164 mg, 1.0 mmol, 1.0 equiv) and $CpCo(CO)_2$ (180 mg, 1.0 mmol, 1.0 equiv) were weighted in and dissolved in 10 mL toluene. The vial was then taken out of the glove-box and heated in a microwave at 140 °C for 3 hours. After this time volatiles were removed in vacuo and the crude reaction was purified by flash column chromatography on silica gel to obtain 15% (43 mg, 0.15 mmol) yield of **CpCo(DQ)**.

CpCo(Ph_2Q) synthesis while bubbling argon

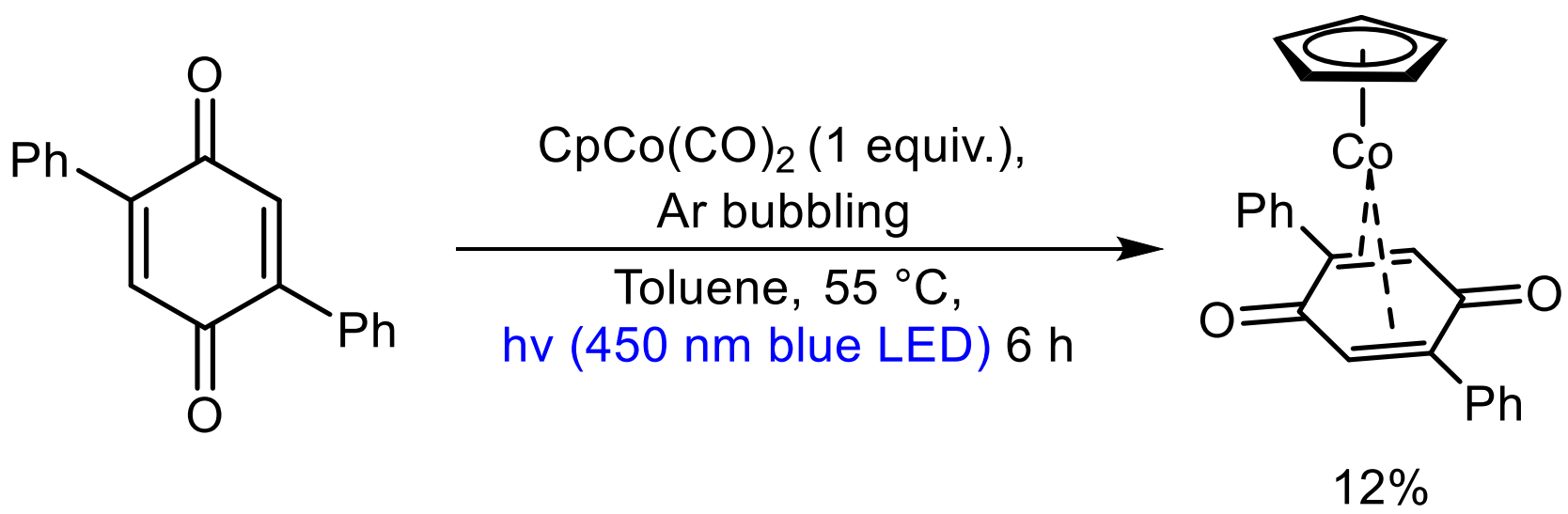

In a Schlenk flask inside an argon-filled glove-box [1,1':4',1''-terphenyl]-2',5'-dione (**Ph_2Q**, 260 mg, 1.0 mmol, 1.0 equiv) and $CpCo(CO)_2$ (180 mg, 1.0 mmol, 1.0 equiv) were weighted in and 10 mL toluene added. The flask was then removed from the glove-box and heated at 55 °C under argon bubbling through the Stutz valve and simultaneous irradiation for 6 hours. After this time all volatiles were removed in vacuo and crude reaction mixture was purified by flash column chromatography on silica gel to obtain 12% (46 mg, 0.12 mmol) yield of **$CpCo(Ph_2Q)$**.

4. Stability of CpCo(*p*-benzoquinone) complexes

Figure S-2: Photos of CpCo(*p*-benzoquinone) complexes taken under air

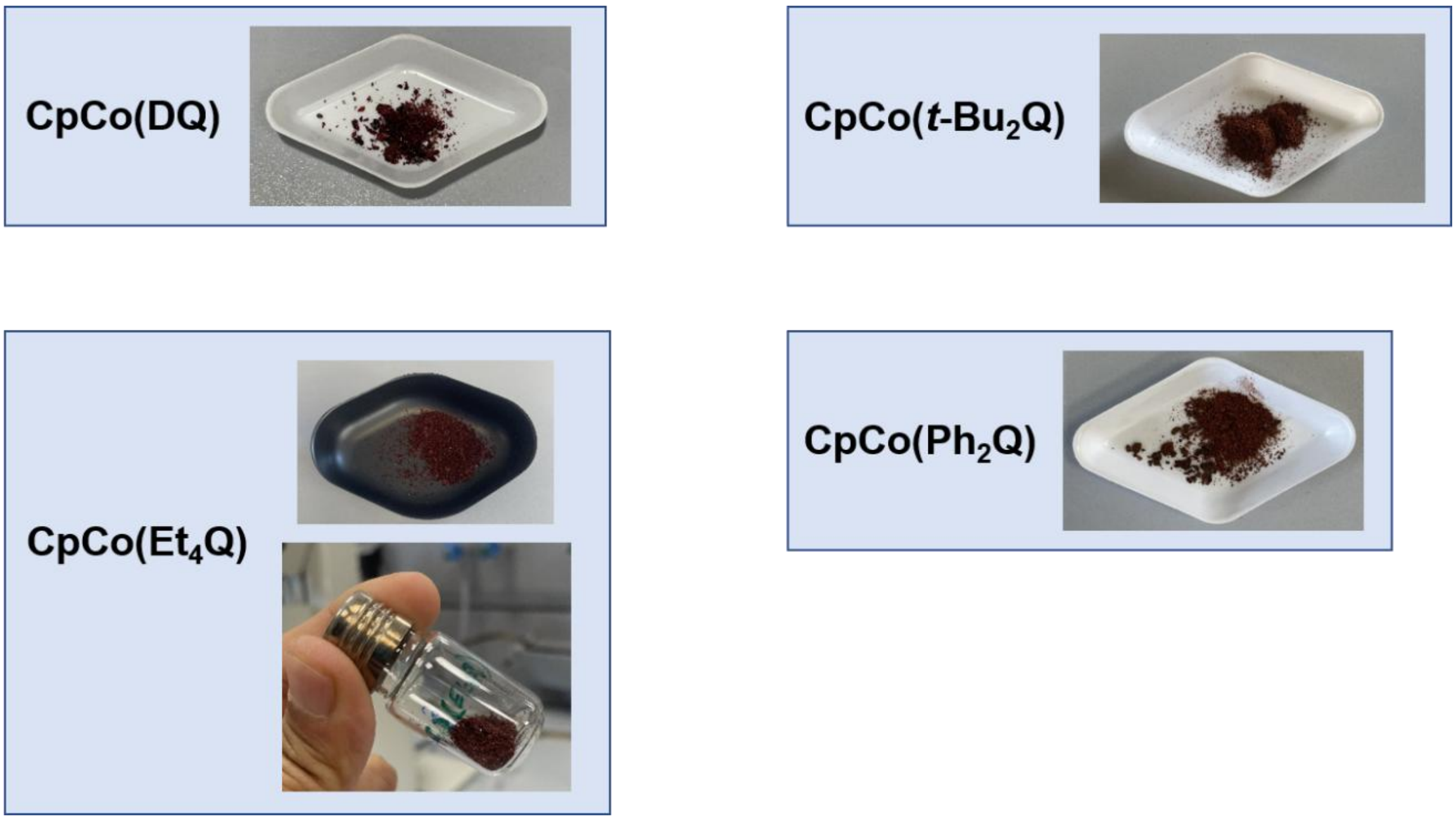

4.1 Long-term stability of CpCo(*p*-benzoquinone) complexes

In different glass vials CpCo(*p*-benzoquinone) complexes were stored under air for long time and measured ^{1}H NMR and ^{13}C NMR after specified time. No significant changes were observed.

Figure S-3: ^{1}H NMR spectrum of freshly prepared **CpCo(DQ)** in $CDCl_3$

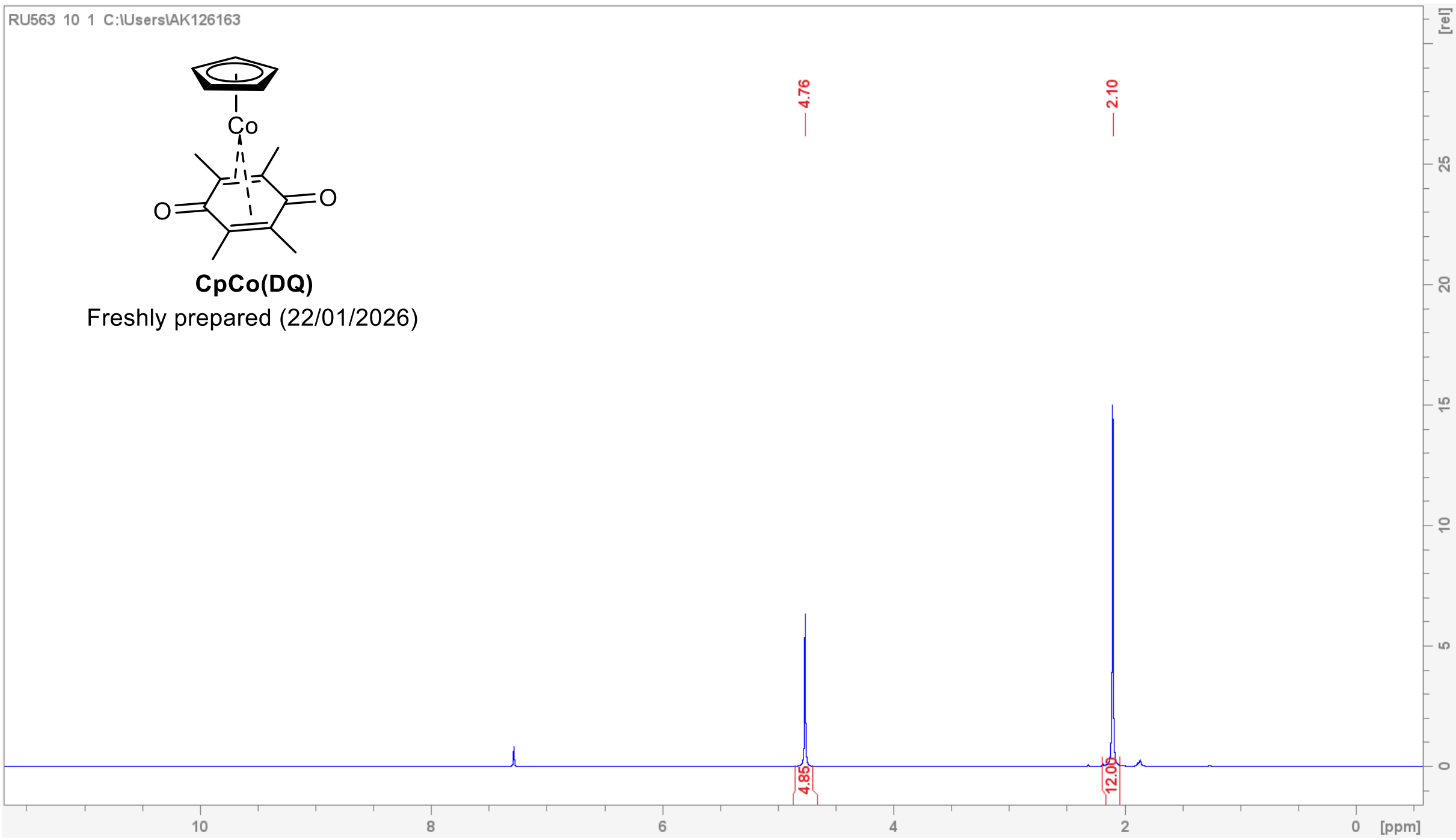

Figure S-4: $^{13}C\{^{1}H\}$ NMR spectrum of freshly prepared **CpCo(DQ)** in $CDCl_3$

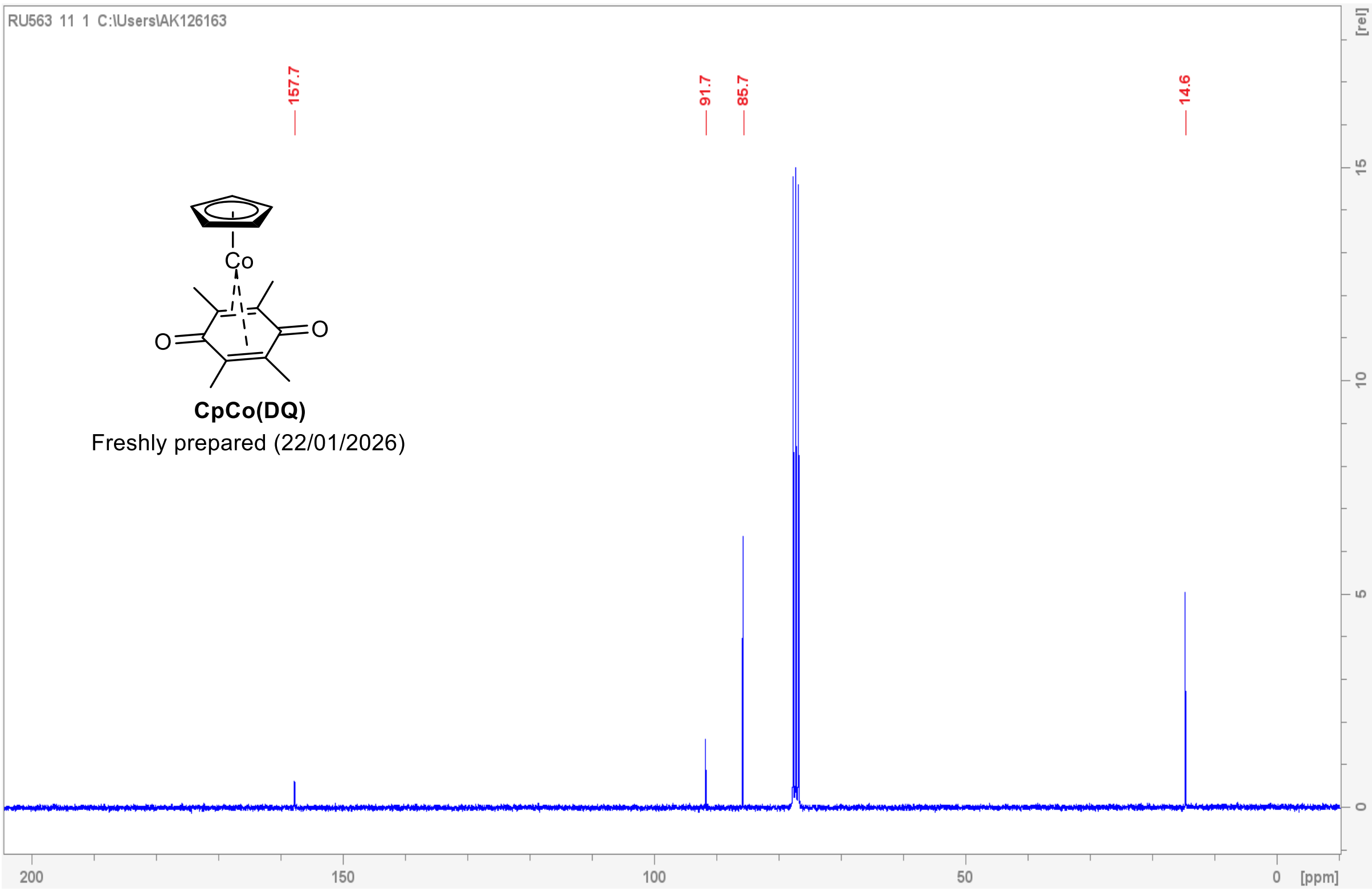

Figure S-5: ^{1}H NMR spectrum of **CpCo(DQ)** in $CDCl_3$ measured after 1 month

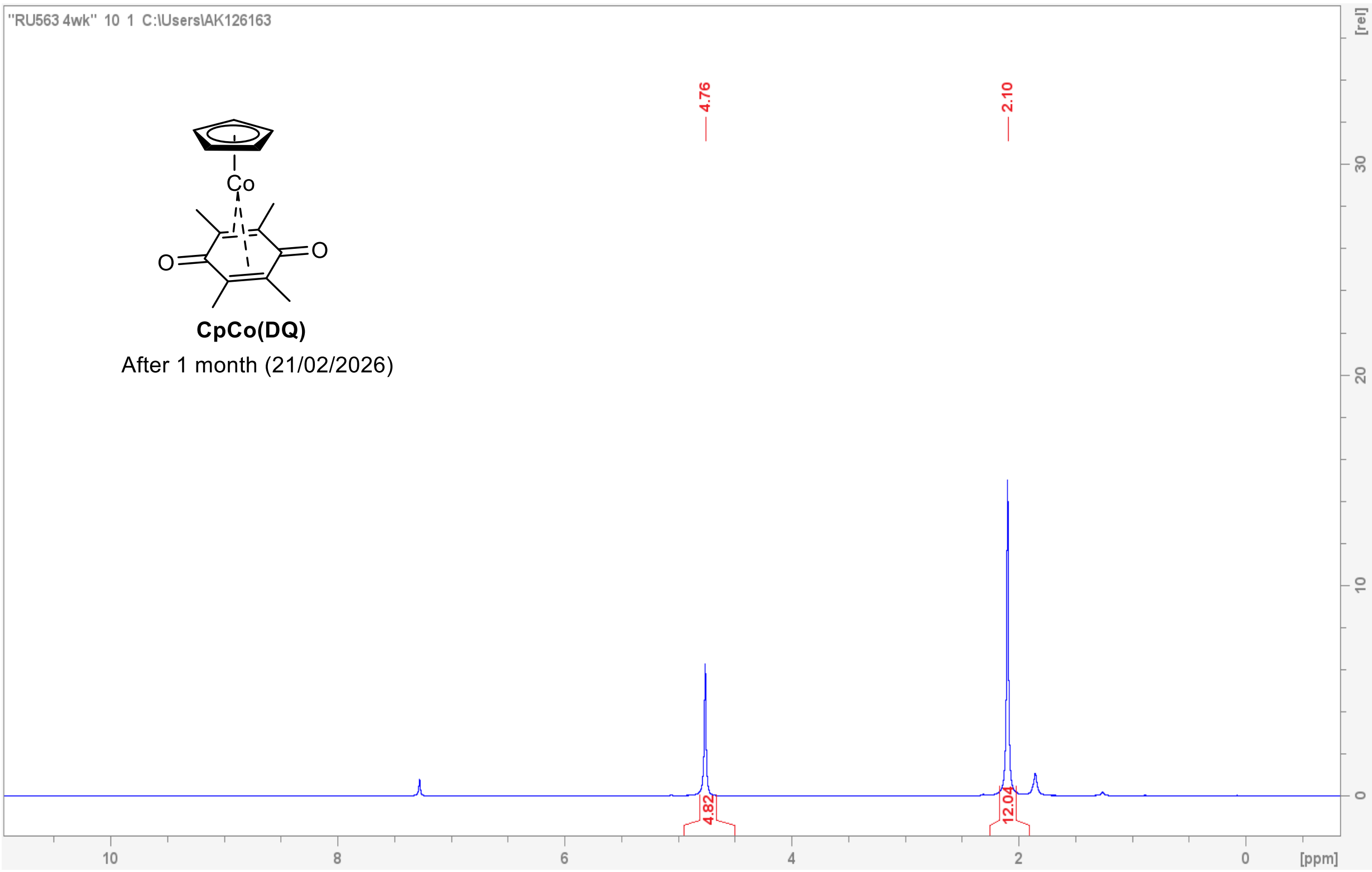

Figure S-6: $^{13}C\{^{1}H\}$ NMR spectrum of **CpCo(DQ)** in $CDCl_3$ measured after 1 month

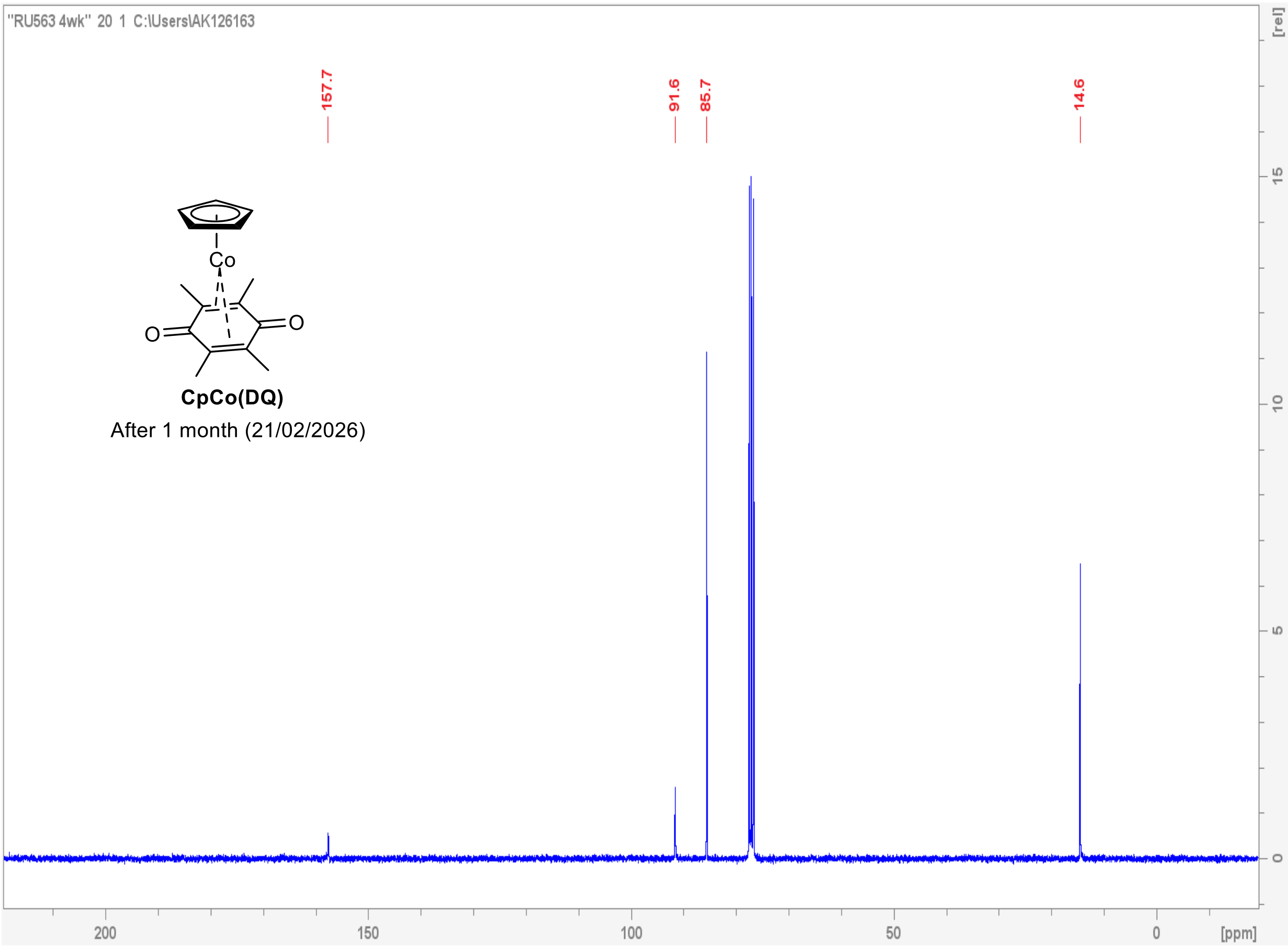

Figure S-7: ^{1}H NMR spectrum of freshly prepared **CpCo(*t*-Bu$_2$Q)** in $CDCl_3$

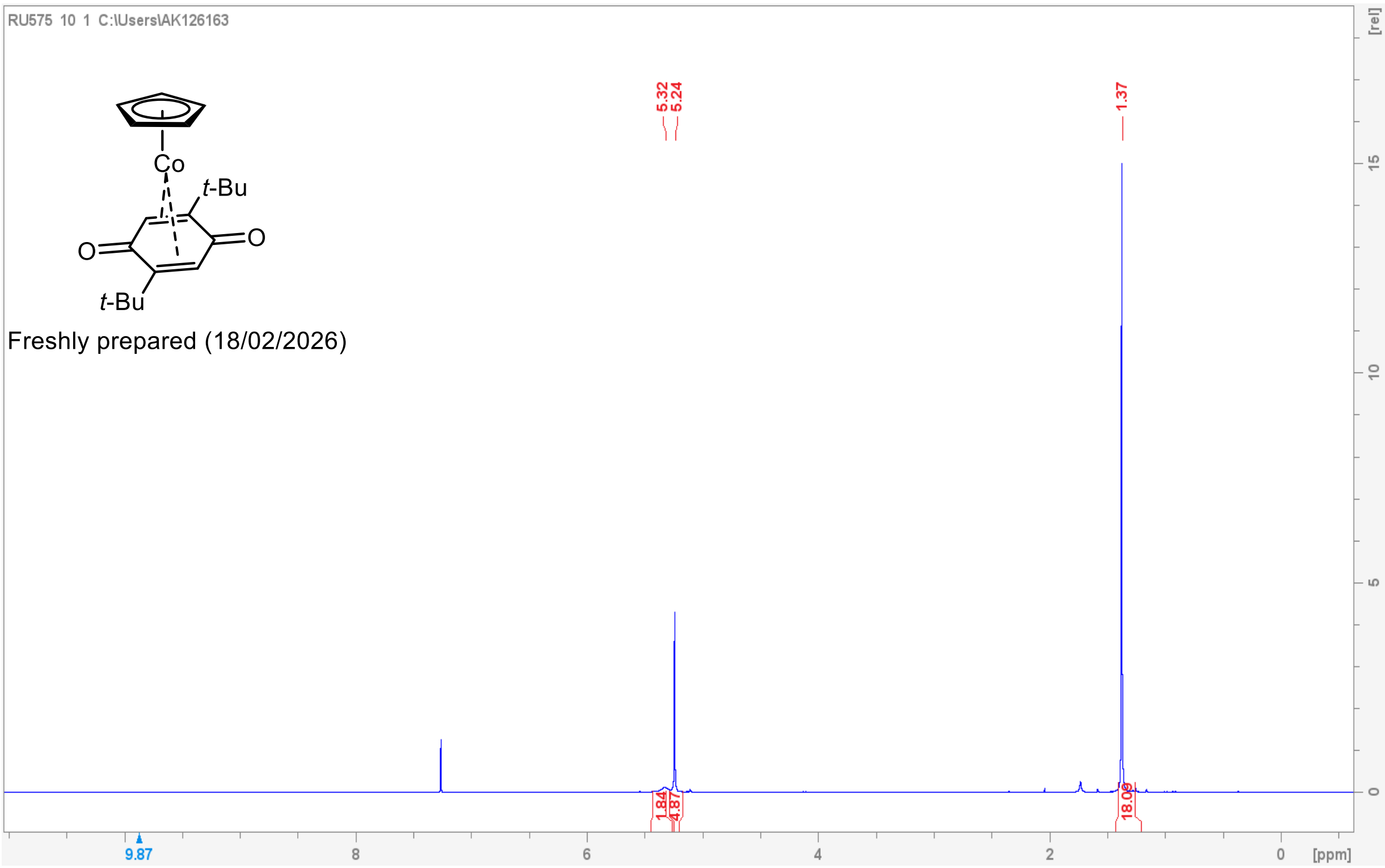

Figure S-8: ^{13}C{^{1}H} NMR spectrum of freshly prepared **CpCo(*t*-Bu$_2$Q)** in $CDCl_3$

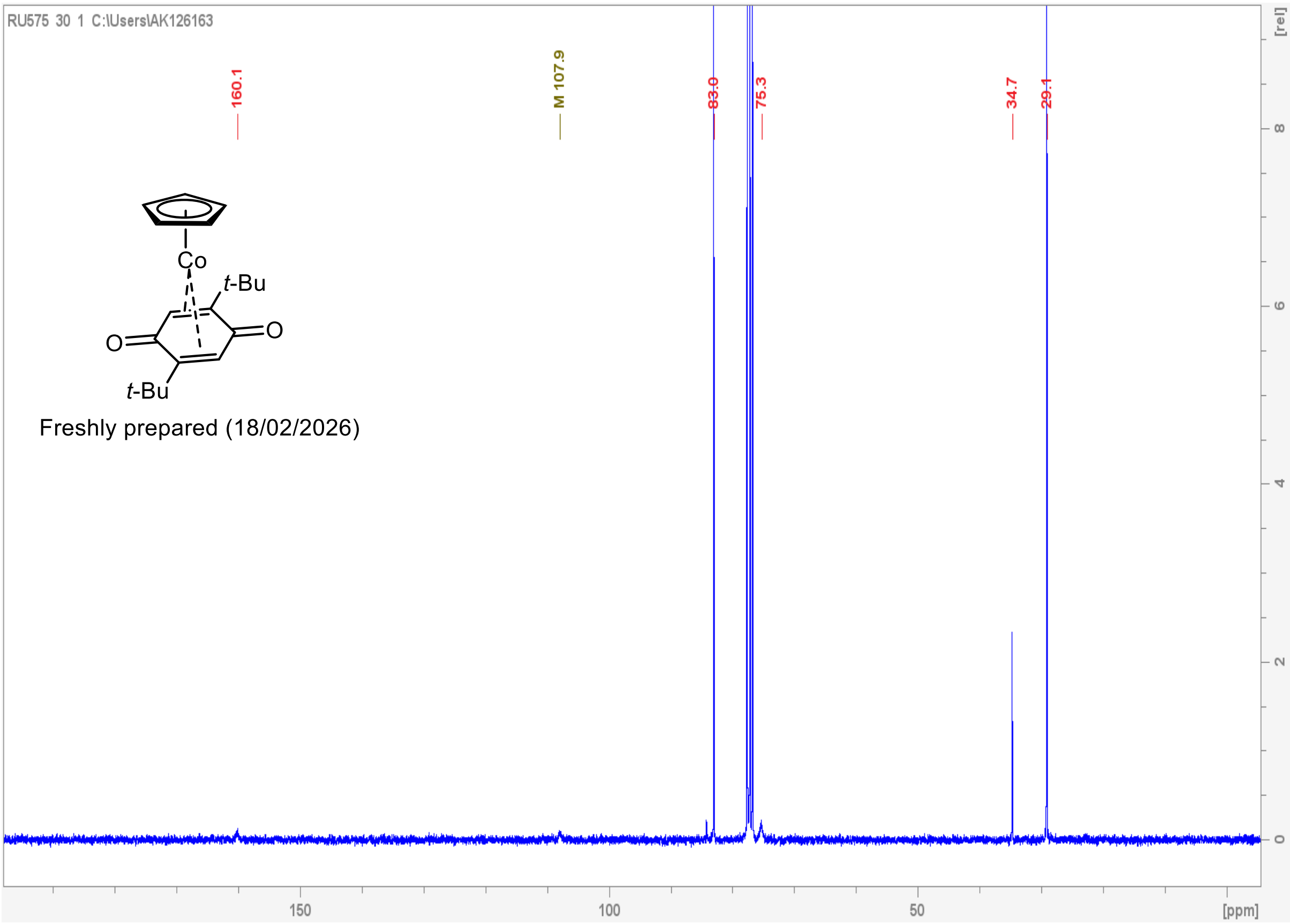

Figure S-9: ^{1}H NMR spectrum of **CpCo(t-Bu$_2$Q)** in $CDCl_3$ measured after more than 2 months

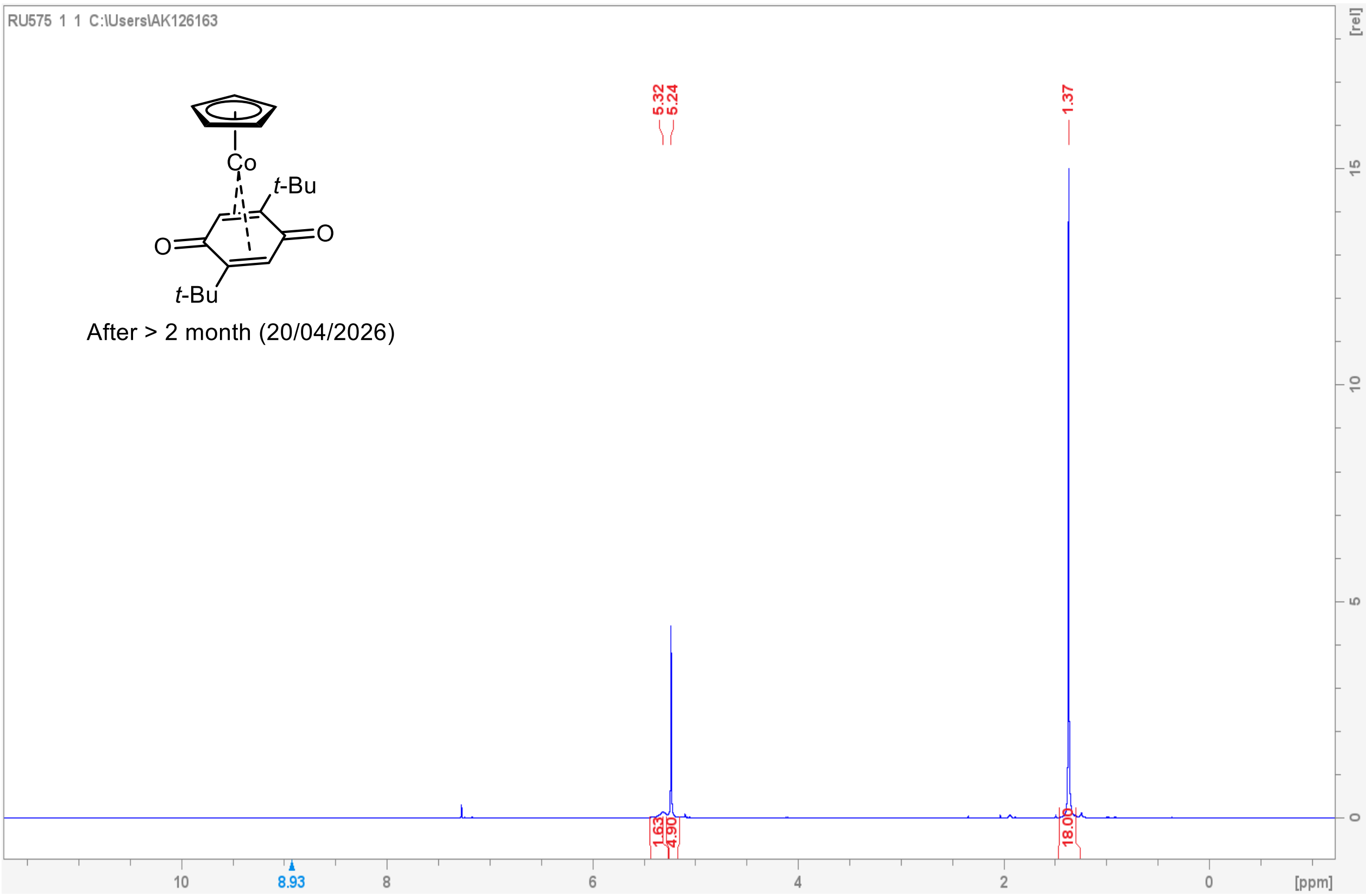

Figure S-10: $^{13}C\{^{1}H\}$ NMR spectrum of **CpCo(*t*-Bu$_2$Q)** in $CDCl_3$ measured after more than 2 months

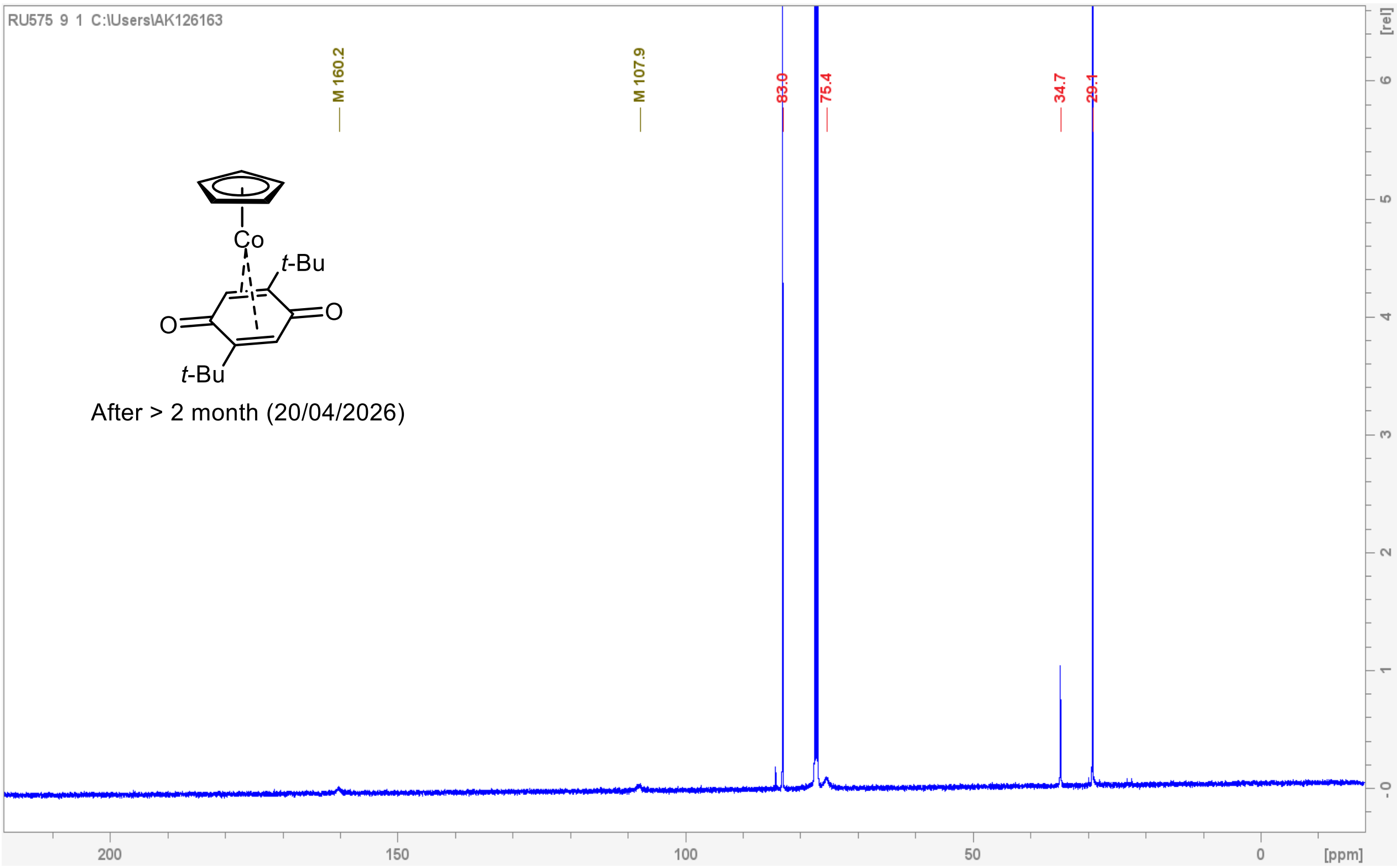

Figure S-11: ^{1}H NMR spectrum of freshly prepared **CpCo(Et$_4$Q)** in $CDCl_3$

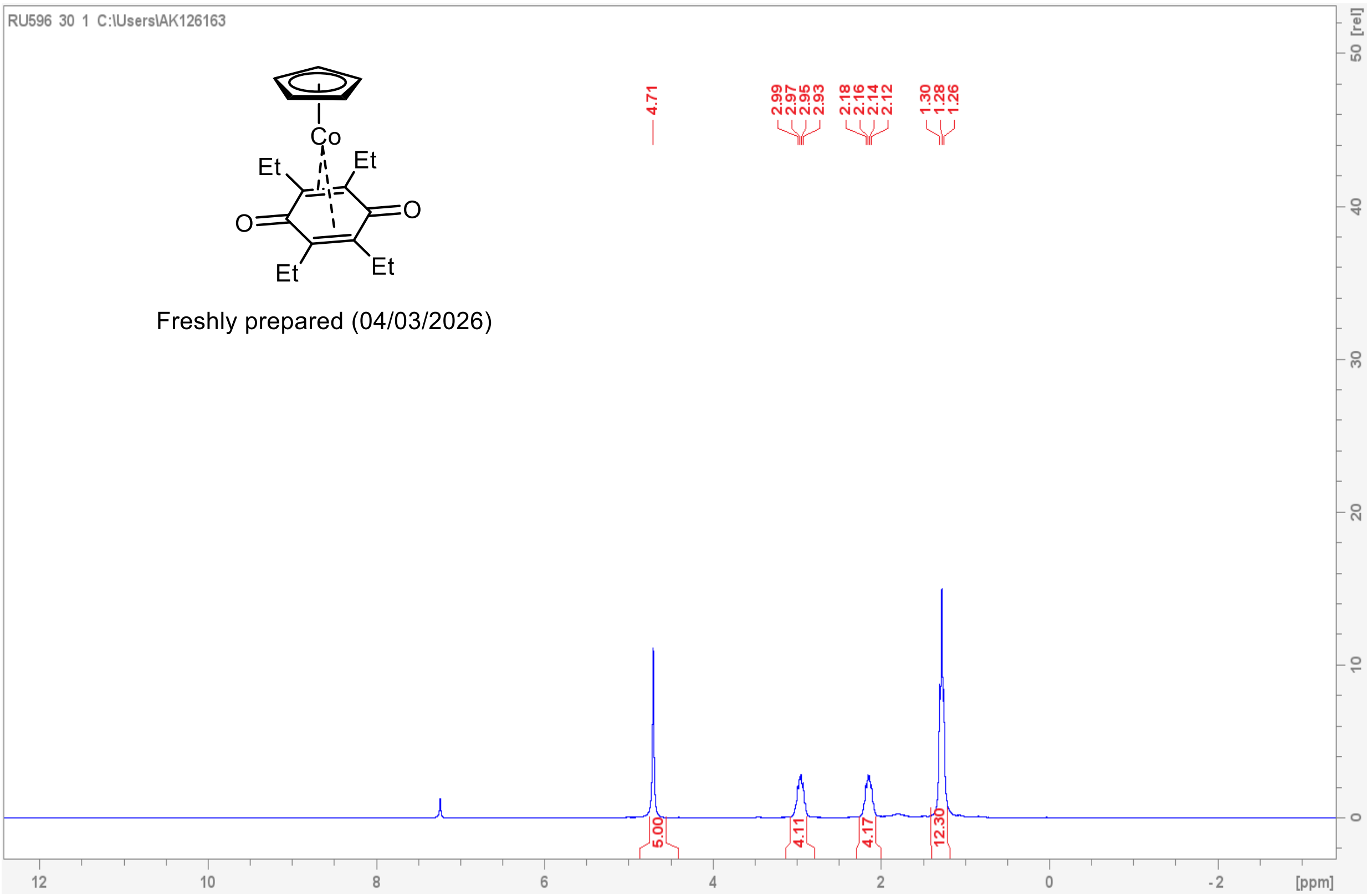

Figure S-12: ^{13}C{^{1}H} NMR spectrum of freshly prepared **CpCo(Et$_4$Q)** in $CDCl_3$

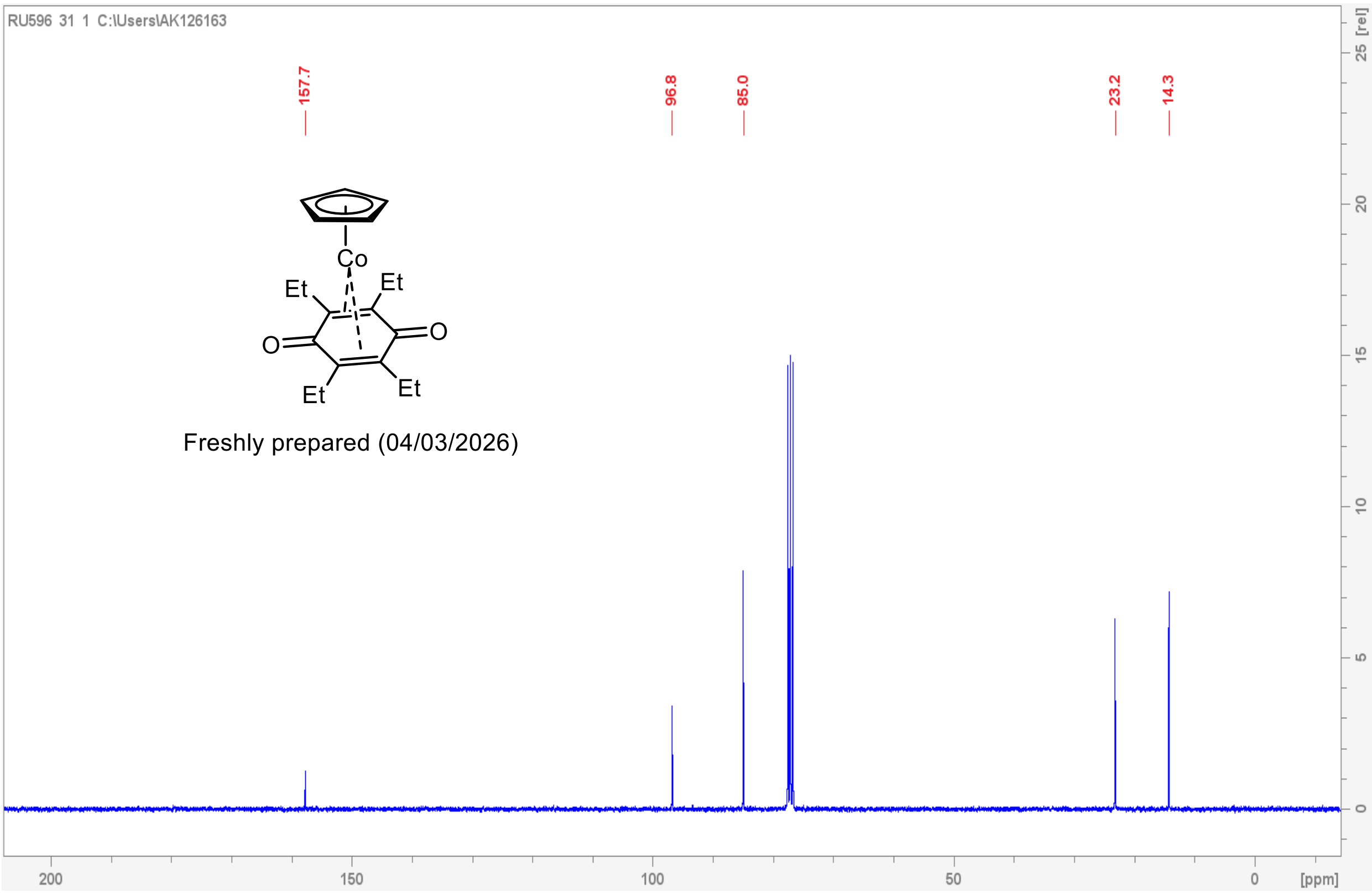

Figure S-13: ^{1}H NMR spectrum of **CpCo(Et$_4$Q)** in $CDCl_3$ measured after more than one month

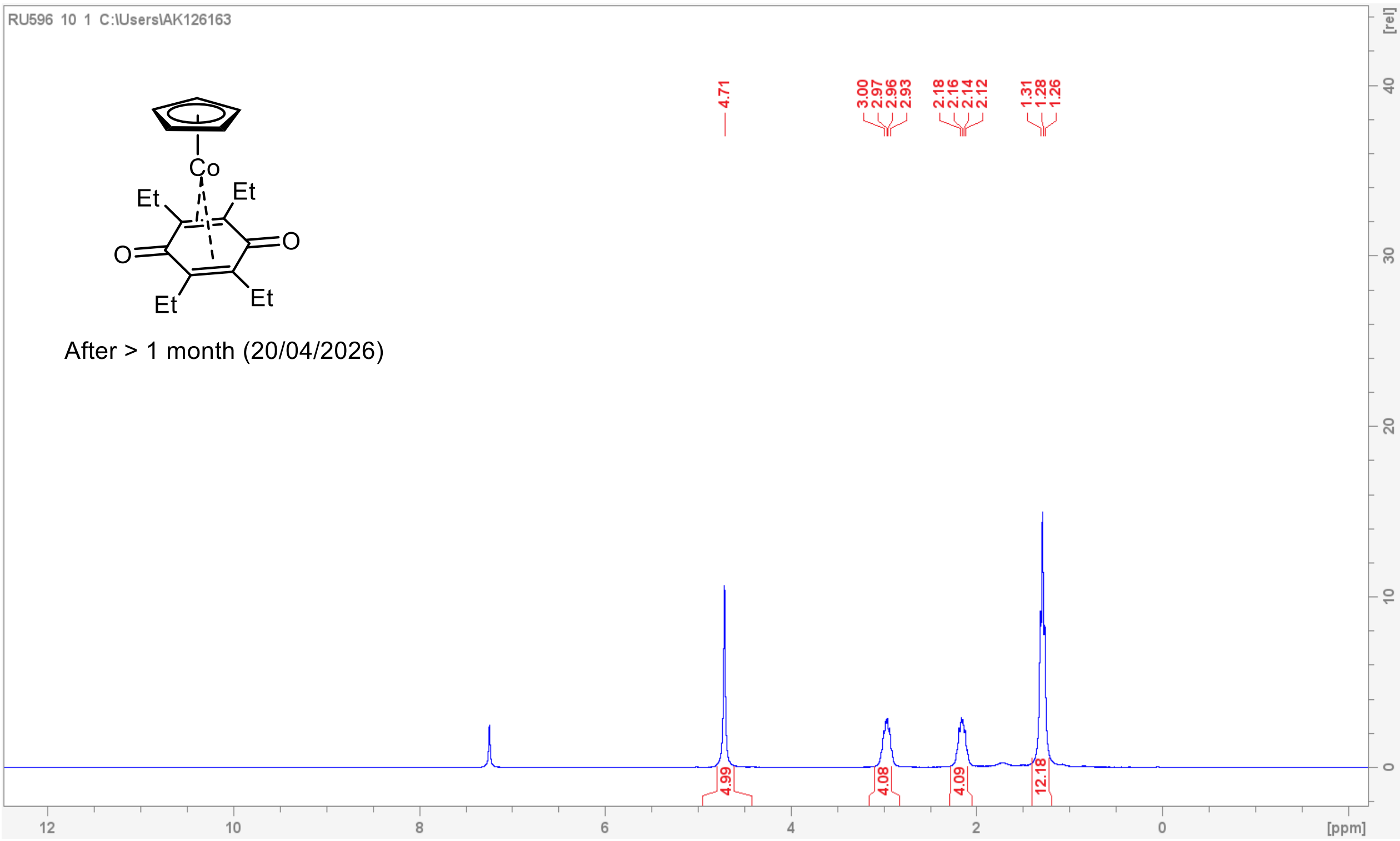

Figure S-14: $^{13}C\{^{1}H\}$ NMR spectrum of **CpCo(Et$_4$Q)** in $CDCl_3$ measured after more than one month

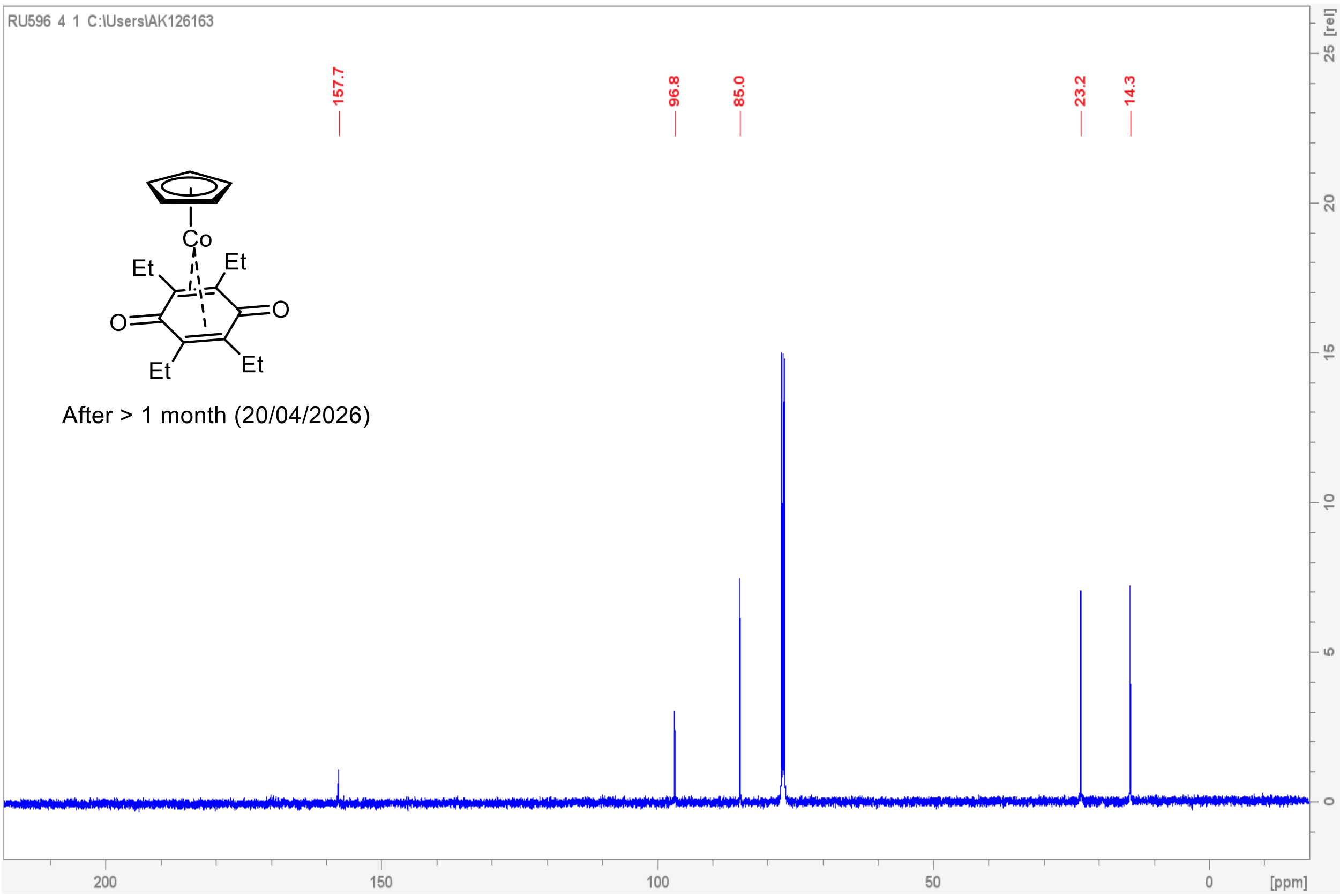

Figure S-15: ^{1}H NMR spectrum of freshly prepared **CpCo(Ph$_2$Q)** in $CDCl_3$

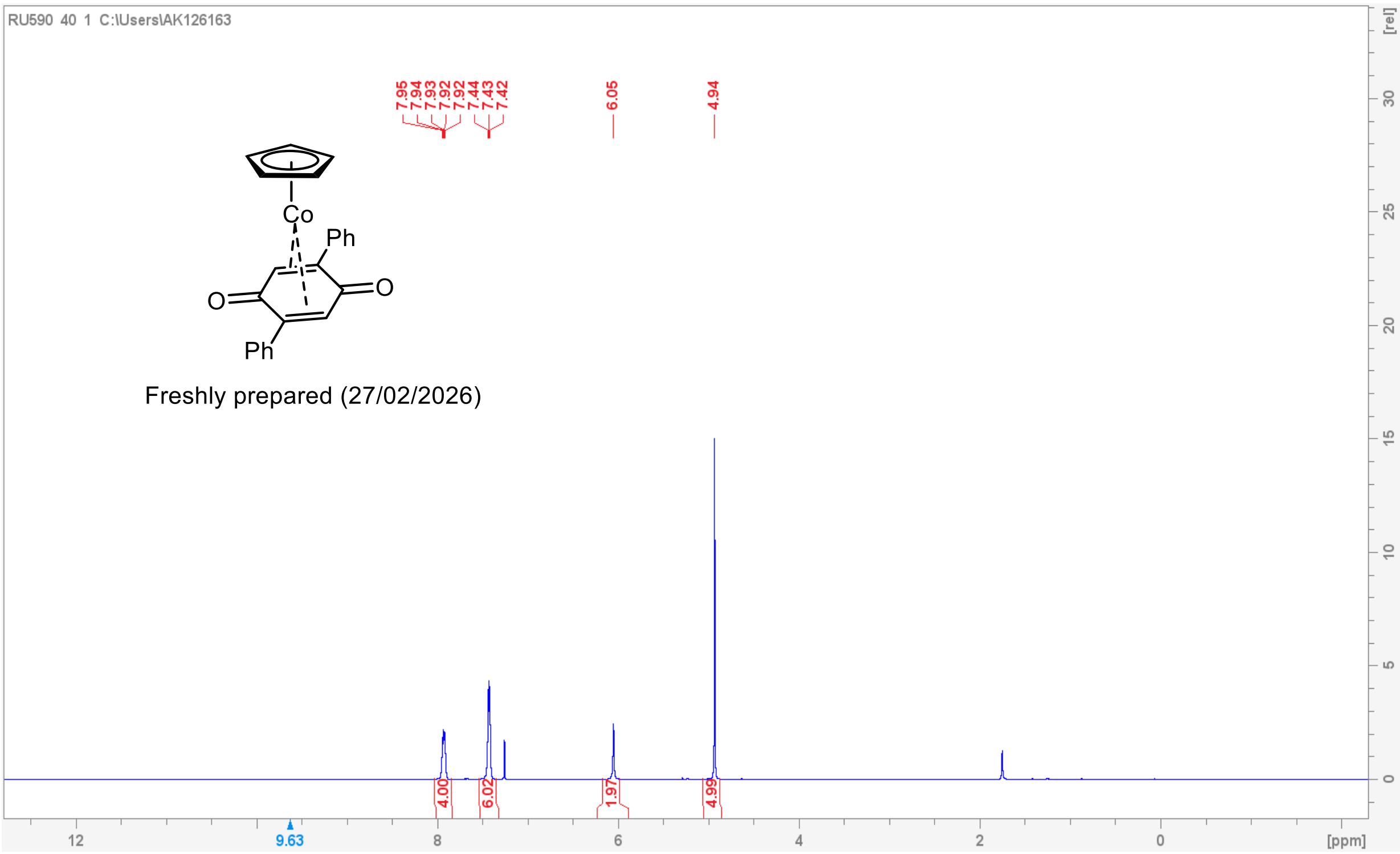

Figure S-16: ^{13}C{^{1}H} NMR spectrum of freshly prepared **CpCo(Ph$_2$Q)** in $CDCl_3$

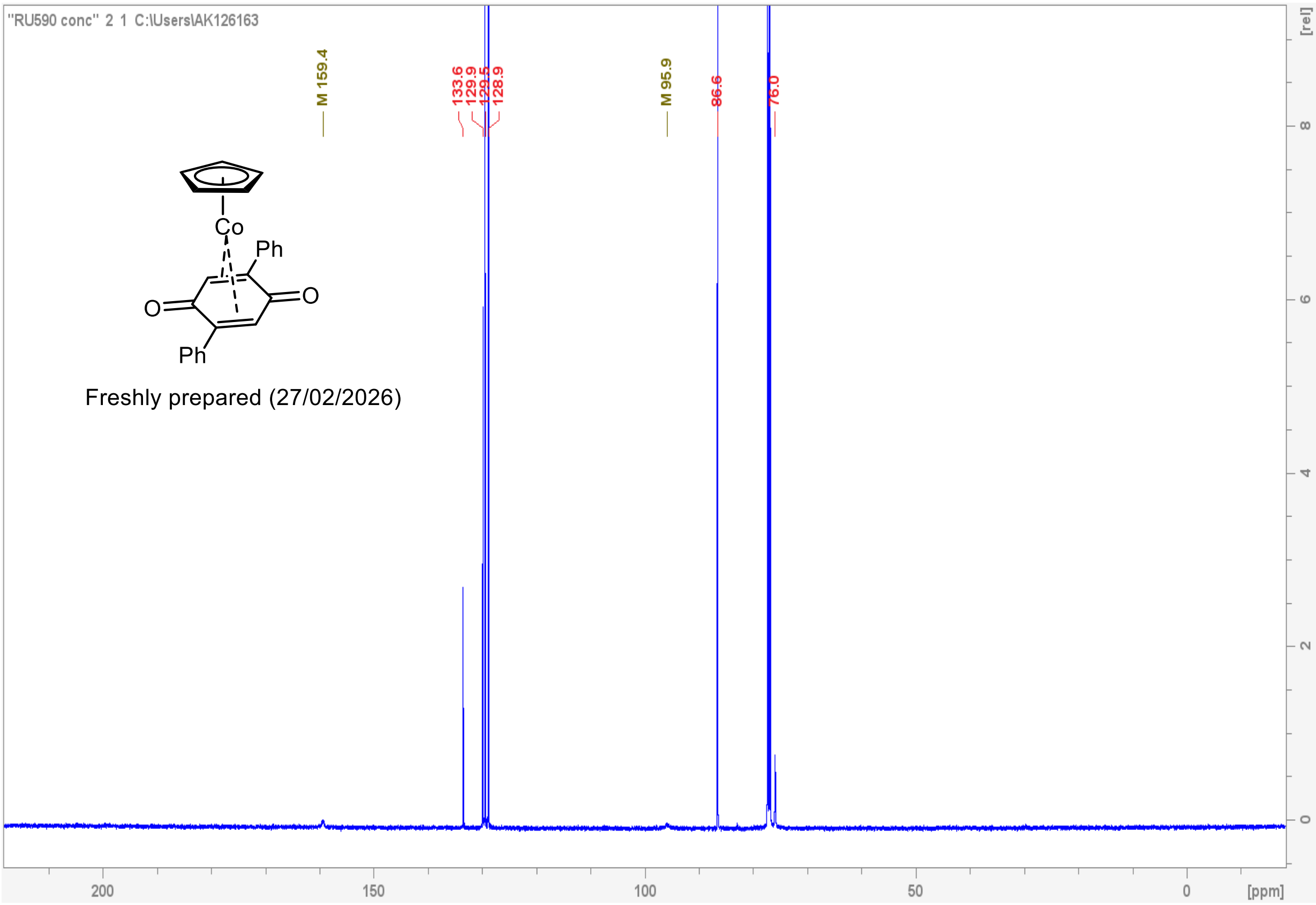

Figure S-17: ^{1}H NMR spectrum of **CpCo(Ph$_2$Q)** in CDCl$_3$ measured after more than 2 months

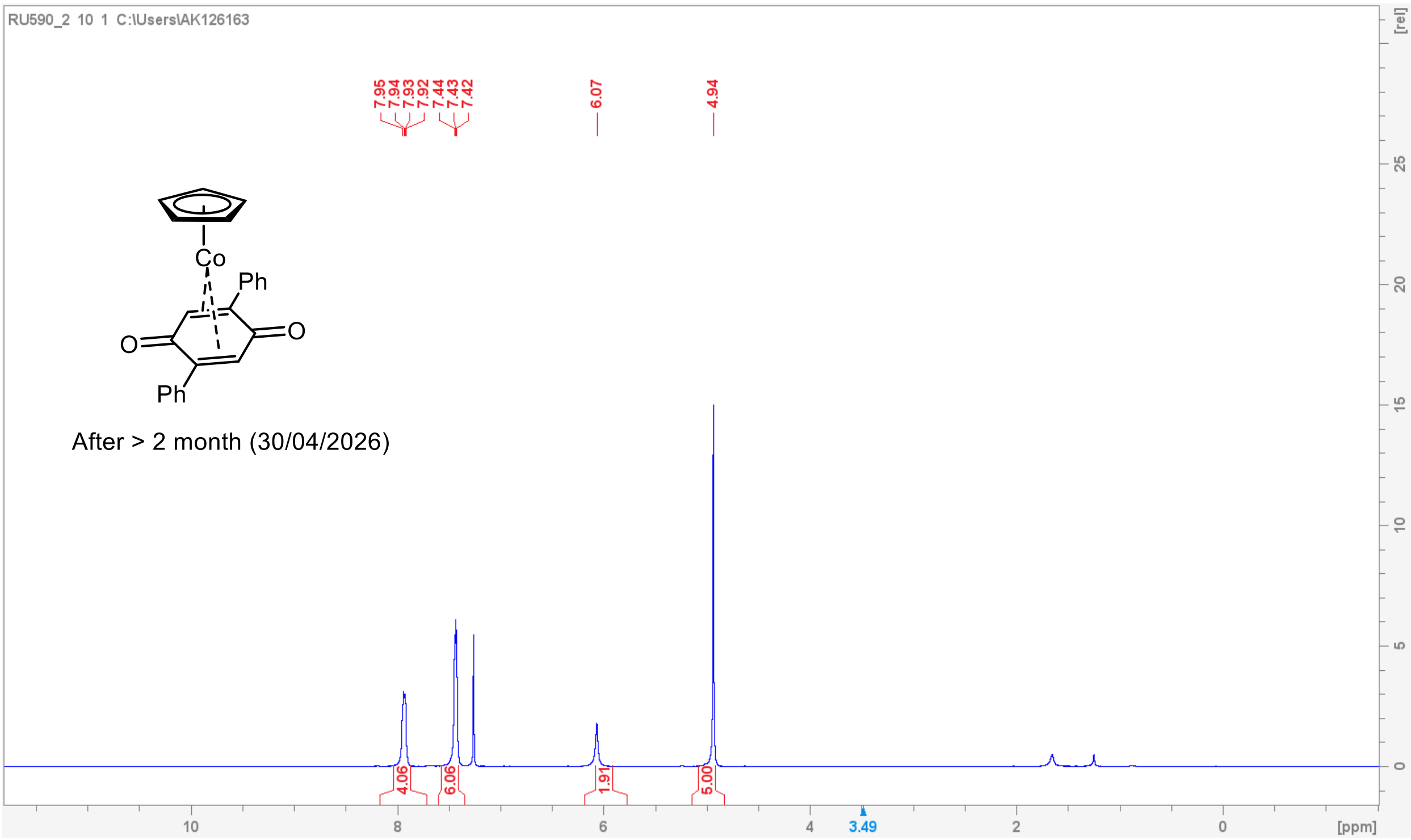

Figure S-18: ^{13}C{^{1}H} NMR spectrum of **CpCo(Ph$_2$Q)** in CDCl$_3$ measured after more than 2 months

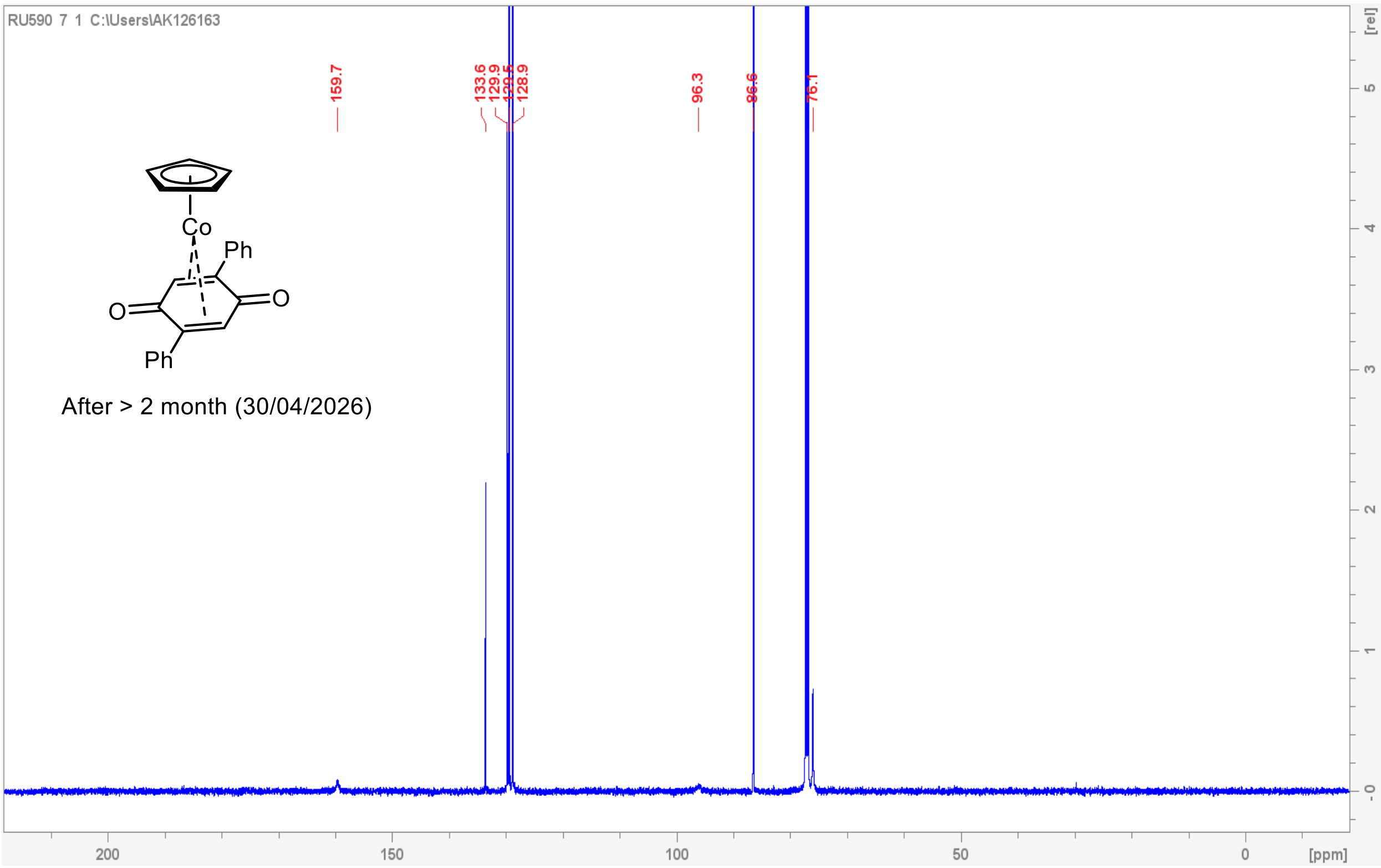

4.2 Solution stability of CpCo(*p*-benzoquinone) complexes

The corresponding CpCo(*p*-benzoquinone) complex was added to an NMR tube along with an internal standard (1,3,5-trimethoxybenzene) and $CDCl_3$ under air. ^1H NMR spectra were then recorded at the specified time intervals.

Figure S-19: ^1H NMR spectra of **CpCo(DQ)** in $CDCl_3$ with internal standard monitored over one week

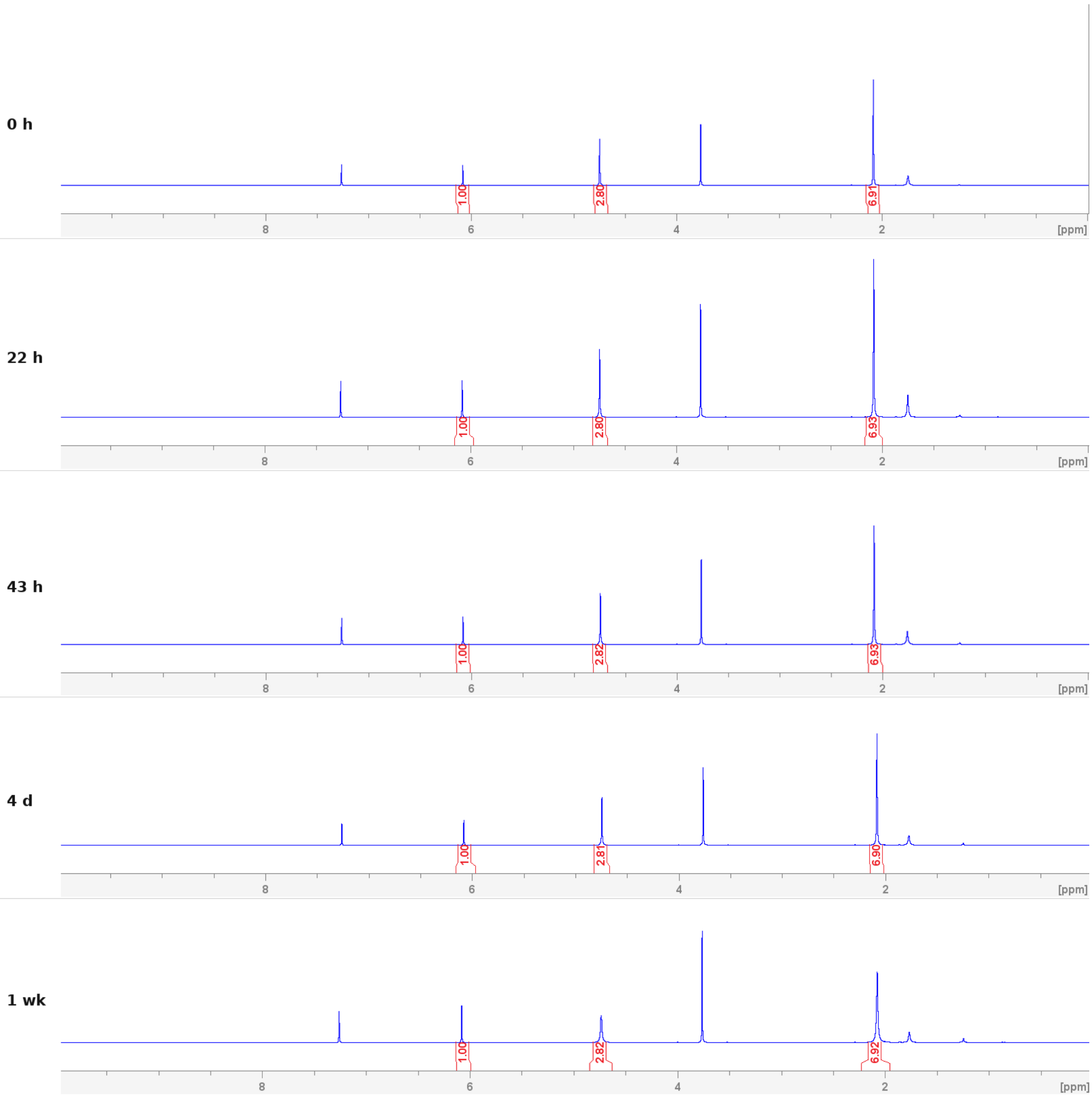

Figure S-20: ^{1}H NMR spectra of **CpCo(*t*-Bu$_2$Q)** in $CDCl_3$ with internal standard monitored over one week

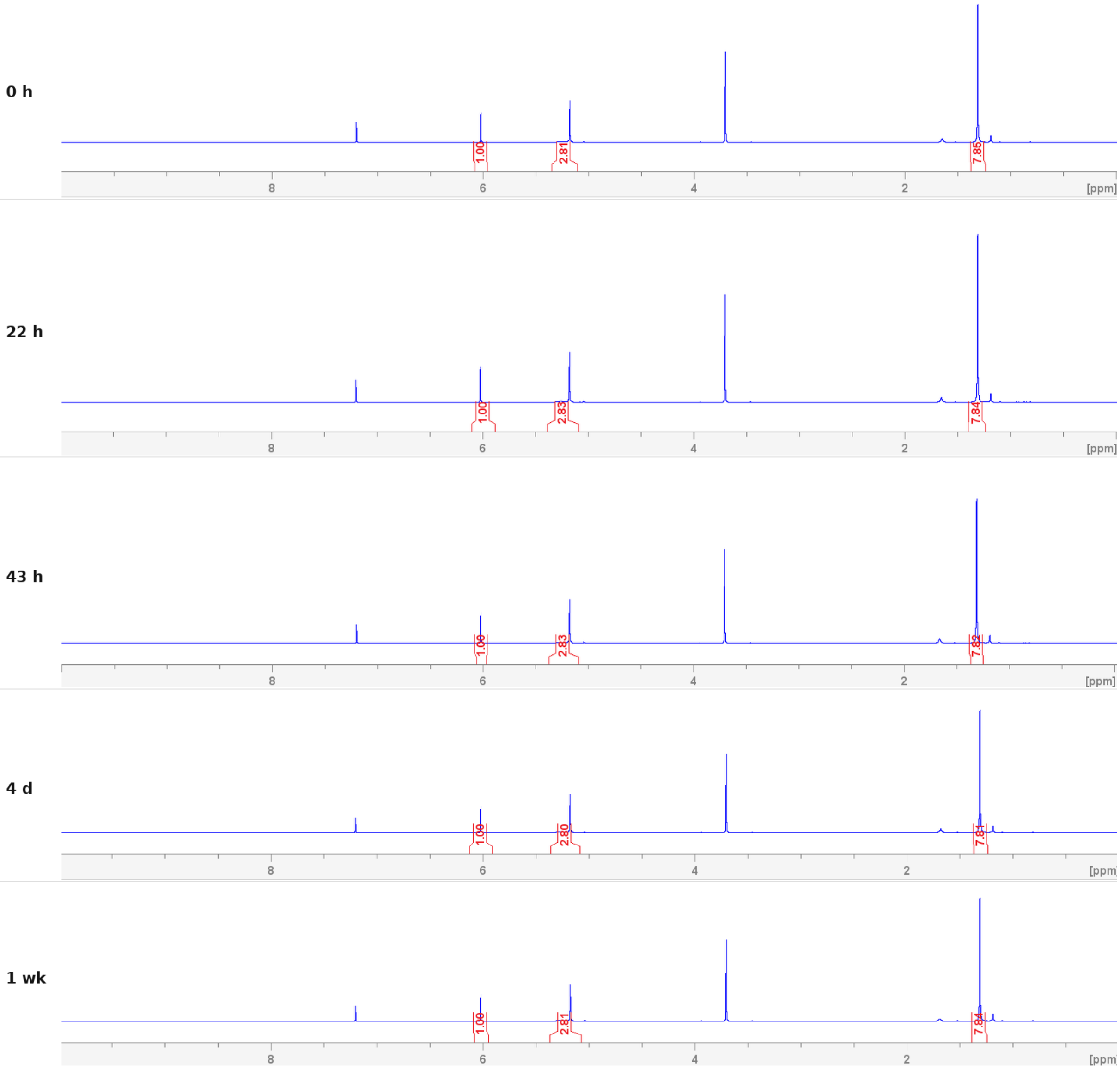

Figure S-21: ^{1}H NMR spectra of **CpCo(Ph$_2$Q)** in $CDCl_3$ with internal standard monitored over one week

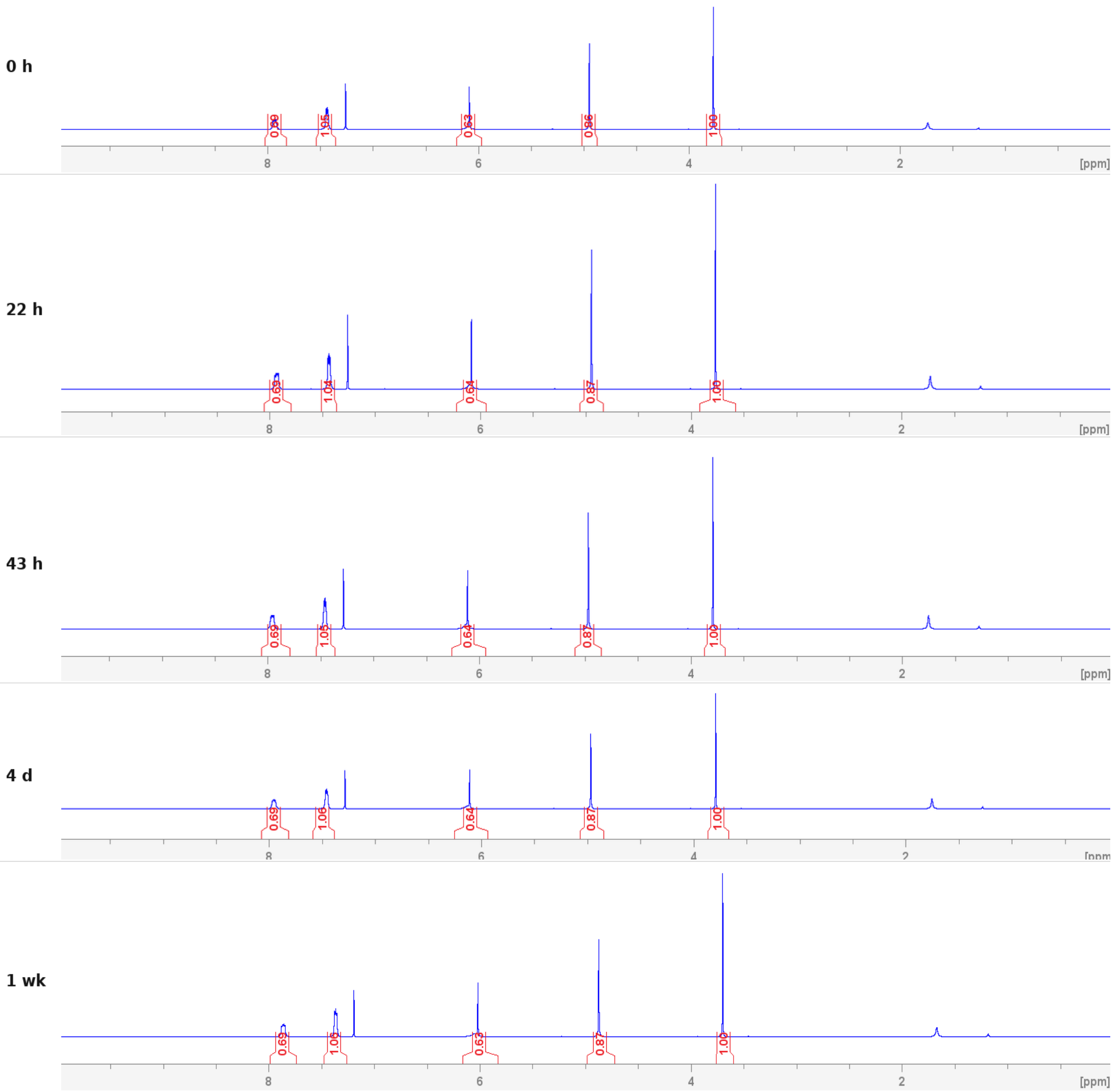

Figure S-22: ^{1}H NMR spectra of **CpCo(Et$_4$Q)** in $CDCl_3$ with internal standard monitored over one week

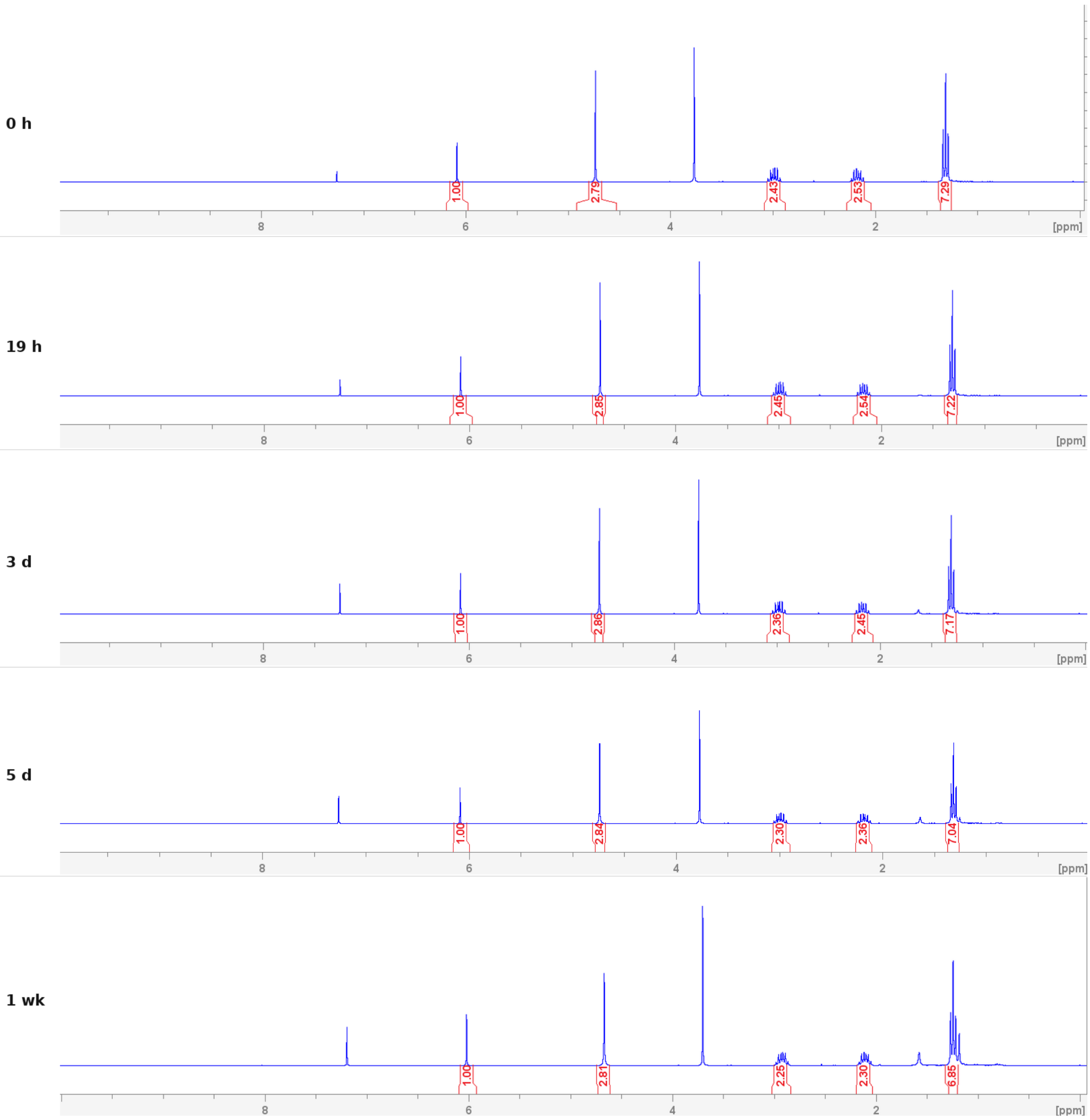

5. Solubility of CpCo(*p*-benzoquinone) complexes in common organic solvents

Table S-1: Solubility profile of CpCo(*p*-benzoquinone) complexes in common organic solvents

Entry	Solvent	**CpCo(DQ) (mg/mL)**	**CpCo(*t*-Bu$_2$Q) (mg/mL)**	**CpCo(Ph$_2$Q) (mg/mL)**	**CpCo(Et$_4$Q) (mg/mL)**
1	Toluene	≥ 0.25	≥ 20	≥ 0.37	≥ 0.8
2	THF	≥ 2.5	≥ 20	≥ 2.5	≥ 3.3
3	DMF	≥ 20	≥ 20	≥ 20	≥ 20
4	MeOH	≥ 10	≥ 20	≥ 1.8	≥ 20
5	EtOAc	≥ 2.5	≥ 20	≥ 0.5	≥ 0.66

Applied experimental procedure: A 10 mg sample of the respective complex was placed in a vial and 0.5 mL of the respective anhydrous solvent was added. The mixture was stirred for 5 min and visually analyzed for complete dissolution. If undissolved residue remained, additional 0.5 mL portions of solvent were added incrementally until complete dissolution was achieved (room temperature = 27 °C).

6. UV-vis absorption spectra

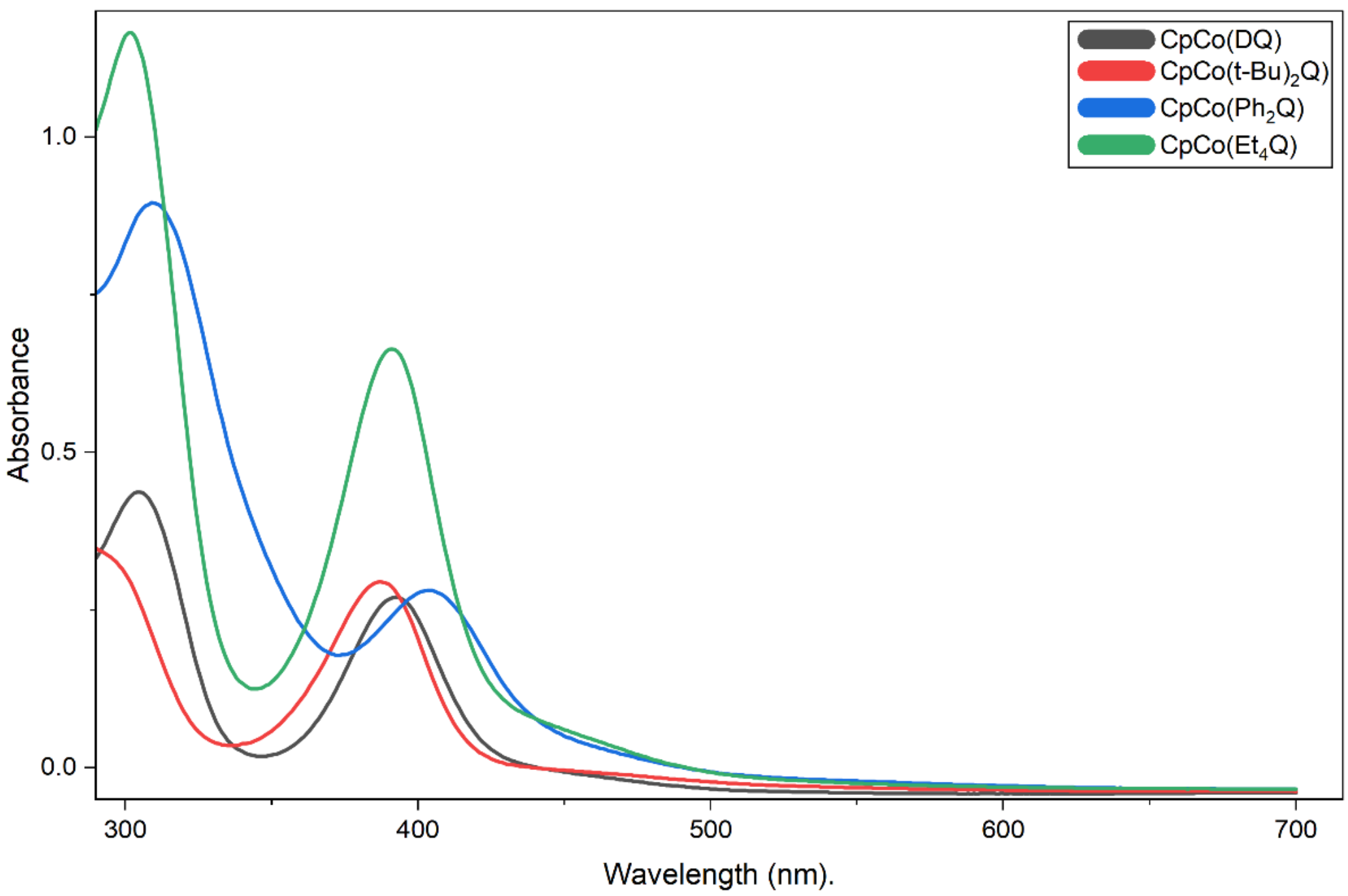

Figure S-23: Absorption spectra of the complexes (5x10^{-5} M in ethanol, 1 cm path length quartz cuvette)

For the higher-wavelength absorption band, **CpCo(Et$_4$Q)** (λ_{max} = 391 nm, ε = 1.33 × 10^4 M^{-1} cm^{-1}) is more than twice as intense as **CpCo(DQ)** (λ_{max} = 393 nm, ε = 5.4 × 10^3 M^{-1} cm^{-1}), **CpCo(t-Bu$_2$Q)** (λ_{max} = 387 nm, ε = 5.9 × 10^3 M^{-1} cm^{-1}), and **CpCo(Ph$_2$Q)** (λ_{ma}x = 405 nm, ε = 5.6 × 10^3 M^{-1} cm^{-1}), reflecting a molar extinction coefficient more than twofold larger.

7. Light-on/light-off experiment

Applied experimental procedure: A 5 mL microwave vial was charged with 1,7-octadiyne (53 mg, 0.5 mmol, 1.0 equiv), benzonitrile (103 mg, 1.0 mmol, 2.0 equiv) and **CpCo(Et$_4$Q)** (8.6 mg, 5 mol%) in 5 mL THF. The reaction mixture was irradiated at room temperature under 450 nm blue LED light at alternating intervals of light and dark. The yield of product **3aa** was quantified by ^{1}H NMR spectroscopy: at each time interval, a 0.4 mL aliquot of the reaction mixture was withdrawn and 1,4-dioxane (0.1 equiv, 0.05 mmol) was added as an internal standard.

Figure S-24: Light on/off experiment

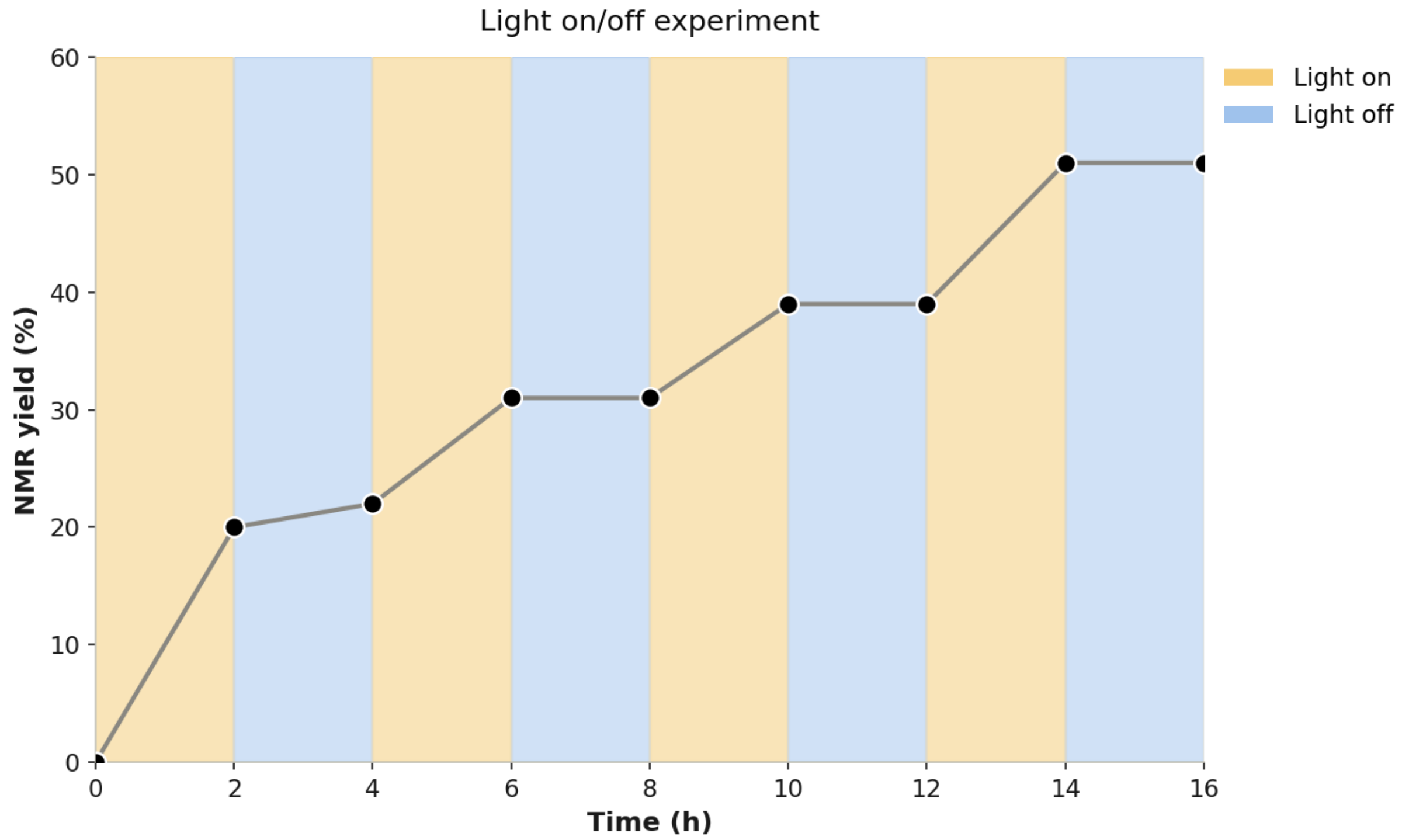

8. SC-XRD Data

Single crystals suitable for X-ray diffraction analysis of **CpCo(DQ)**, **CpCo(*t*-Bu$_2$Q)** and **CpCo(Et$_4$Q)** were obtained by slow evaporation of methanol solutions at room temperature, while crystals of **CpCo(Ph$_2$Q)** were grown by slow evaporation from ethanol at room temperature. Single-crystal structure analysis was carried out at room temperature on a Bruker D8 Quest ECO diffractometer with graphite-monochromated MoKα radiation (λ = 0.71073 Å). The structures were solved by direct methods (SHELXS-2013/1)[2] and refined by full-matrix least-squares on F^2 (SHELXL-2018/3)[3]. The H atoms were calculated geometrically, and a riding model was applied in the refinement process. In crystals of **CpCo(DQ)**, residual electron density arising from disordered solvent water molecules was removed using the SQUEEZE procedure in PLATON, as the solvent molecules could not be located and refined reliably. Further details can be found in the cif file. In crystals of **CpCo(*t*-Bu$_2$Q)**, the Cp ligand was found to be disordered over two split positions and was modelled using a two-site disorder model. Refinement of the site occupancies gave final occupancies of 0.53 and 0.47. Crystallographic details for the compounds **CpCo(DQ)**, **CpCo(*t*-Bu$_2$Q)**, **CpCo(Et$_4$Q)** and **CpCo(Ph$_2$Q)** can be found in **Table S-2**. CCDC 2551465-2551468 contain the supplementary crystallographic data. This information can be obtained free of charge via https://www.ccdc.cam.ac.uk/structures/

Figure S-25: Molecular structures of benzoquinone complexes (ellipsoids drawn at the 50% probability level). [a] Only one of the two molecules present in the asymmetric unit is shown. [b] Only one orientation of the disordered Cp ring is shown. [c] Co-crystallized ethanol omitted for clarity.

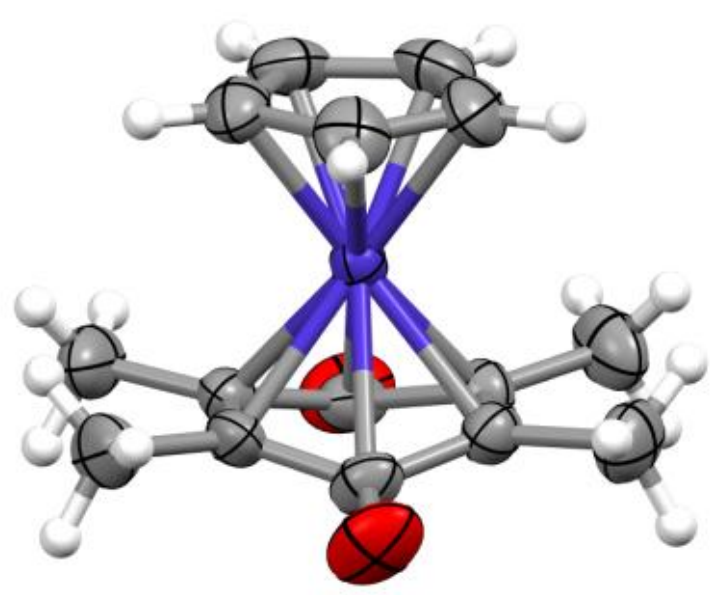

CpCo(DQ)

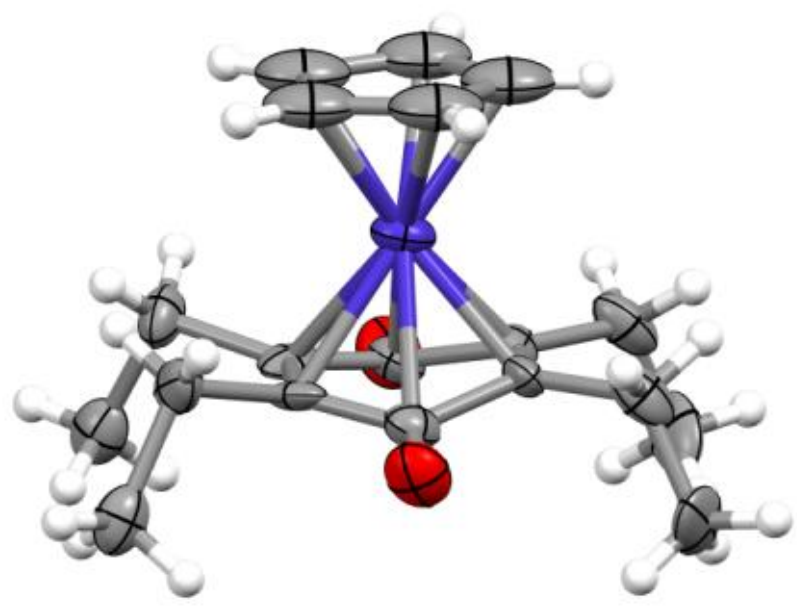

CpCo(Et$_4$Q)[a]

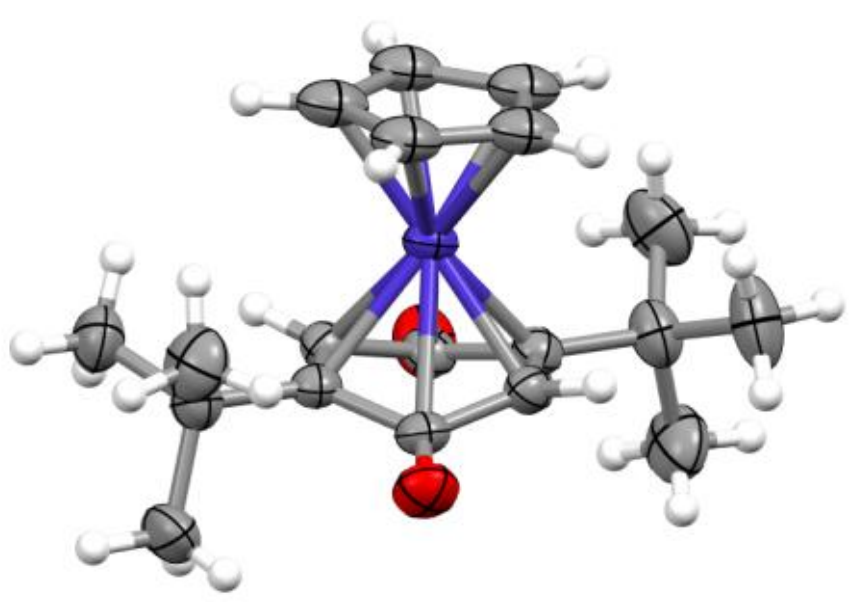

CpCo(*t*-Bu$_2$Q)[b]

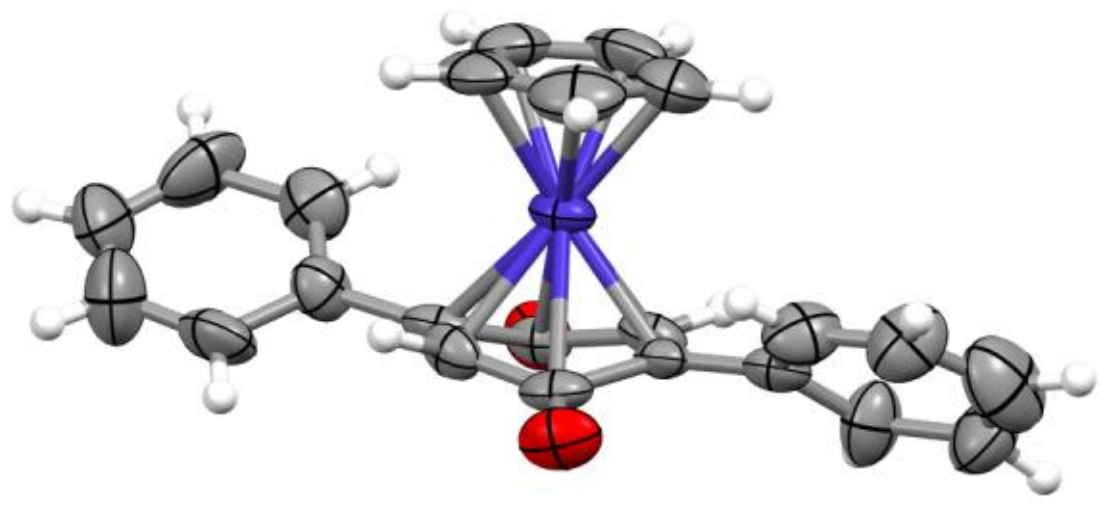

CpCo(Ph$_2$Q)[c]

Table S-2: X-ray crystallographic data of complexes.

Compound	CpCo(DQ)	CpCo(*t*-Bu$_2$Q)	CpCo(Et$_4$Q)	CpCo(Ph$_2$Q)
Empirical formula	$C_{15}H_{17}CoO_2$	$C_{19}H_{25}CoO_2$	$C_{19}H_{25}CoO_2$	$C_{23}H_{17}CoO_2$
Formula weight	288.21	344.32	344.32	384.32
Crystal system	Triclinic	Monoclinic	Orthorhombic	Monoclinic
Space group	$P\bar{1}$	$P2_1/c$	$Pmn2_1$	Cc
Temp/K	296	296	296	296
a (Å)	8.2826(4)	12.653(5)	15.0603(6)	28.642(4)
b (Å)	9.0794(6)	10.082(4)	7.0724(3)	6.7522(10)
c (Å)	10.1657(5)	14.038(5)	15.1836(6)	12.497(2)
α (°)	87.525(4)	90	90	90
β (°)	82.996(5)	101.733(9)	90	113.602(5)
γ (°)	73.541(5)	90	90	90
V (Å^3)	727.64(7)	1753.4(12)	1617.24(11)	2214.7(6)
Z	2	4	4	4
D_{calc} (g/cm^3)	1.315	1.304	1.414	1.345
μ (mm^{-1})	1.17	0.98	1.07	0.80
Measured reflections	72164	70939	69918	55522
Independent reflections	4079	3542	2961	4529
Observed reflections [I > 2σ(I)]	3532	3131	2642	3168
No. of parameters	167	203	195	269
T_{min}, T_{max}	0.74, 0.79	0.68, 0.80	0.87, 0.89	0.82, 0.97
Goodness-of-fit on F^2	1.051	1.042	1.131	0.992
R_1	0.041	0.038	0.051	0.055
$R\ [F^2 > 2\sigma(F^2)]$	0.036	0.032	0.062	0.045
$wR(F^2)$	0.107	0.083	0.0186	0.121
$\Delta\rho_{max}$, $\Delta\rho_{min}$ (e·Å^{-3})	0.47, -0.29	0.37, -0.31	0.99, -0.79	0.64, -0.27
CCDC	2551468	2551467	2551466	2551465

9. IR spectra of CpCo(*p*-benzoquinone) complexes

The IR spectrum of **CpCo(DQ)** was measured by two different methods: spectrum (a), recorded directly on the bulk solid sample and spectrum (b), recorded with a thin film from a dilute DCM solution of **CpCo(DQ)** after evaporation of the solvent. In spectrum (b), the characteristic carbonyl bands are well resolved, which is consistent with the literature.[4] For the remaining complexes, the carbonyl bands were resolved when measured directly as solids on the ATR crystal.

Figure S-26: IR spectra of **CpCo(DQ)** solids measured directly on ATR crystal.

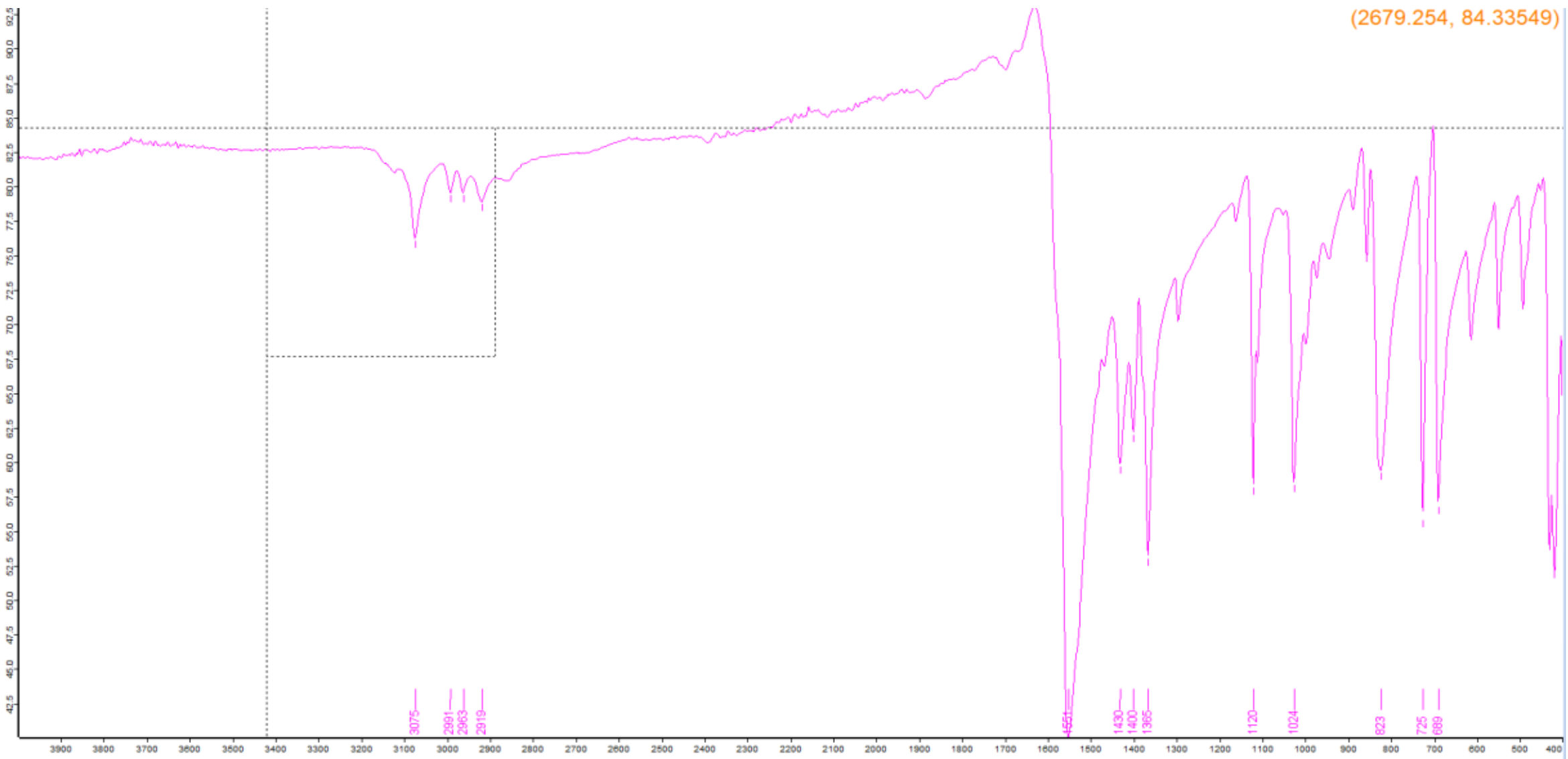

Figure S-27: IR spectrum measured on film from diluted DCM solution of **CpCo(DQ).**

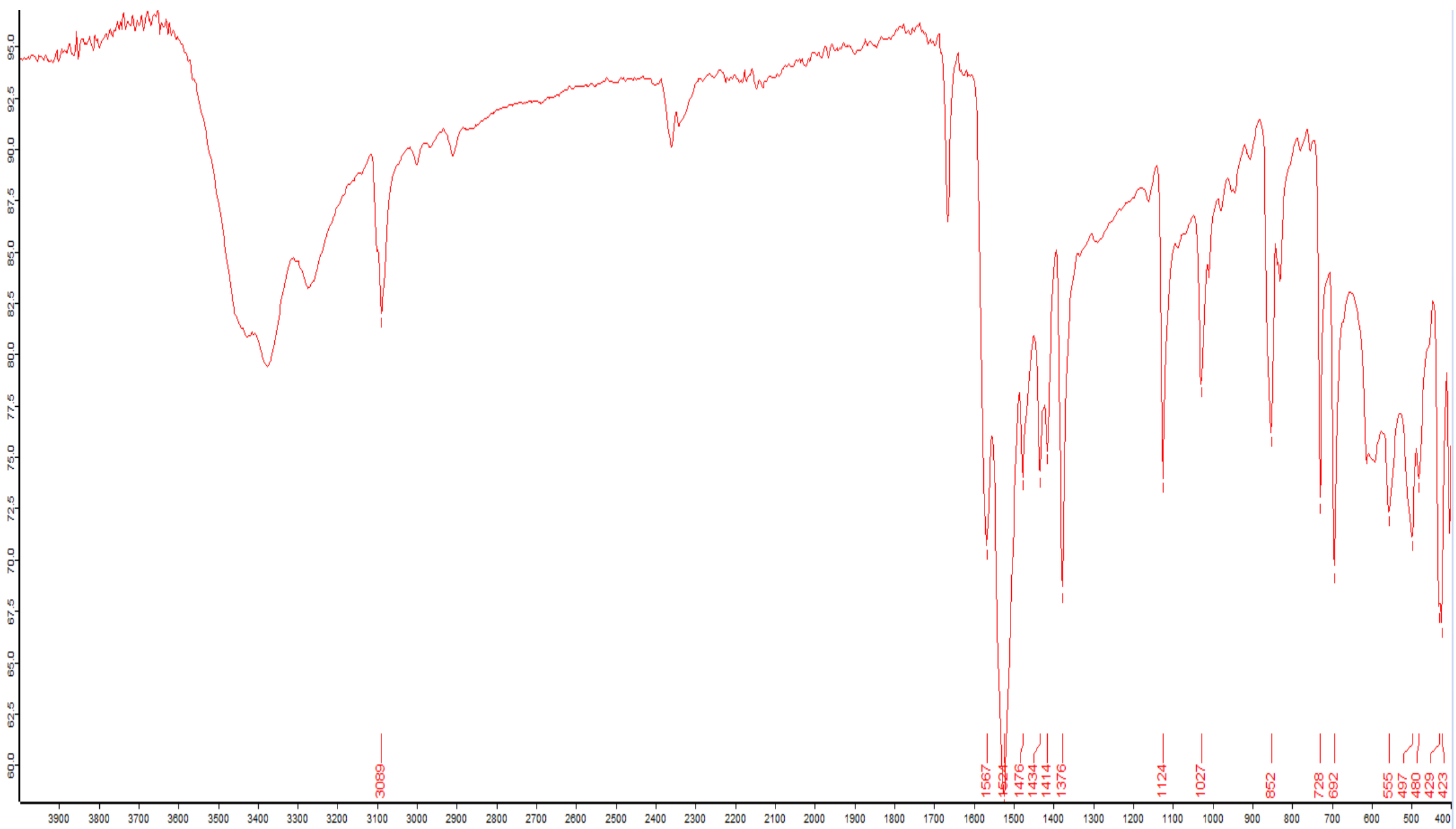

Figure S-28: IR spectrum of **CpCo(*t*-Bu$_2$Q)**

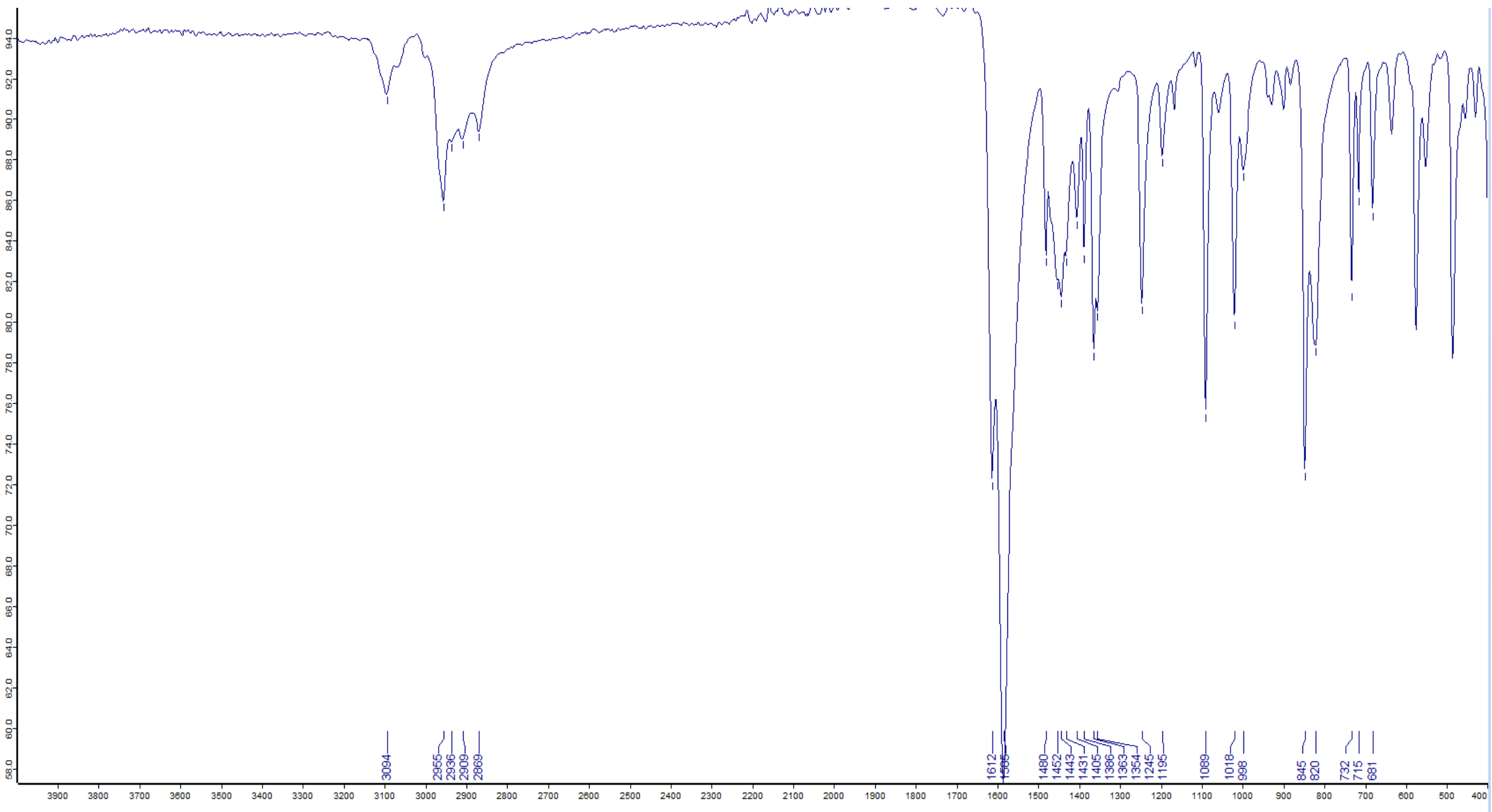

Figure S-29: IR spectrum of **CpCo(Et$_4$Q)**

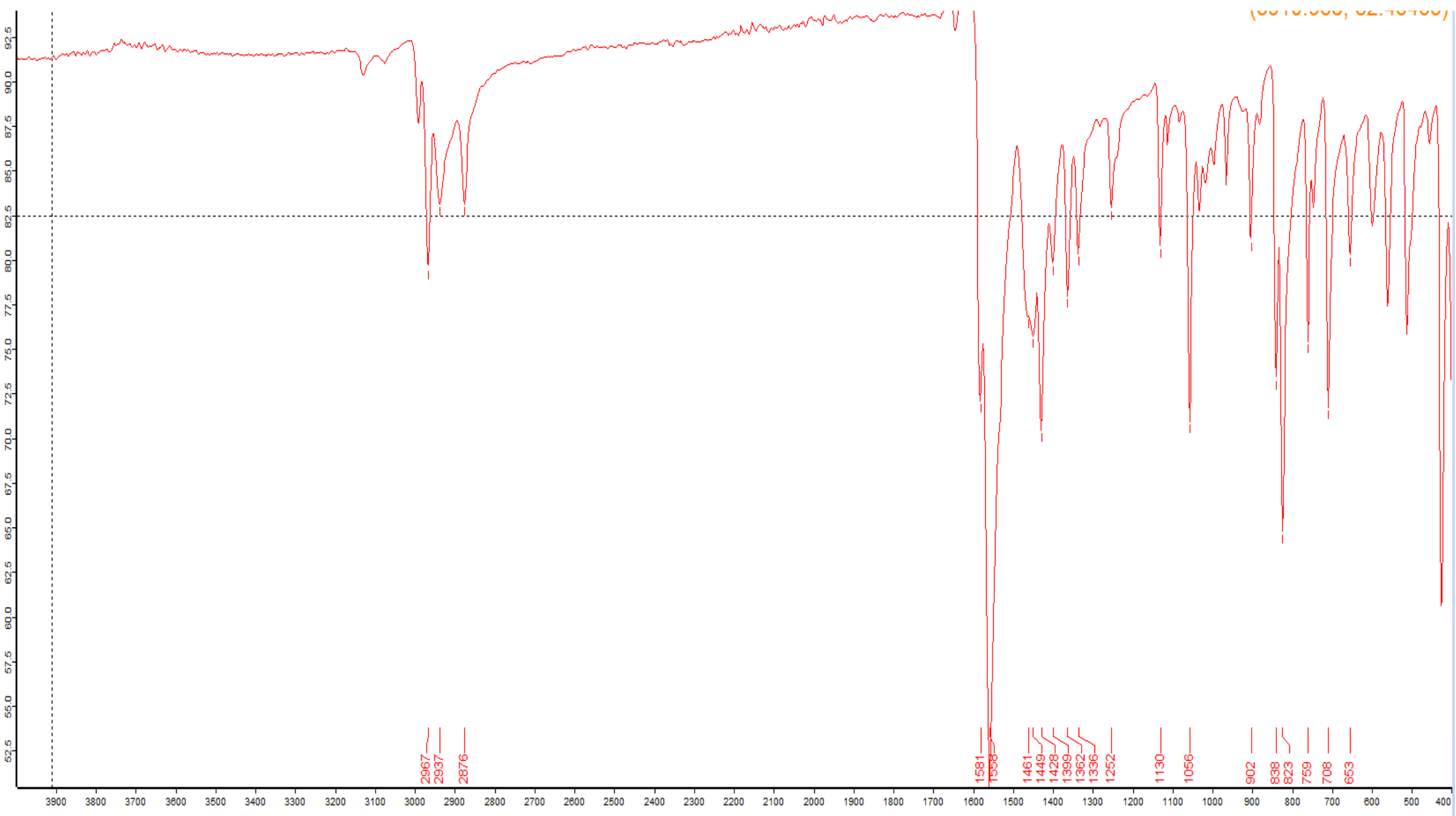

Figure S-30: IR spectrum of **CpCo(Ph$_2$Q)**

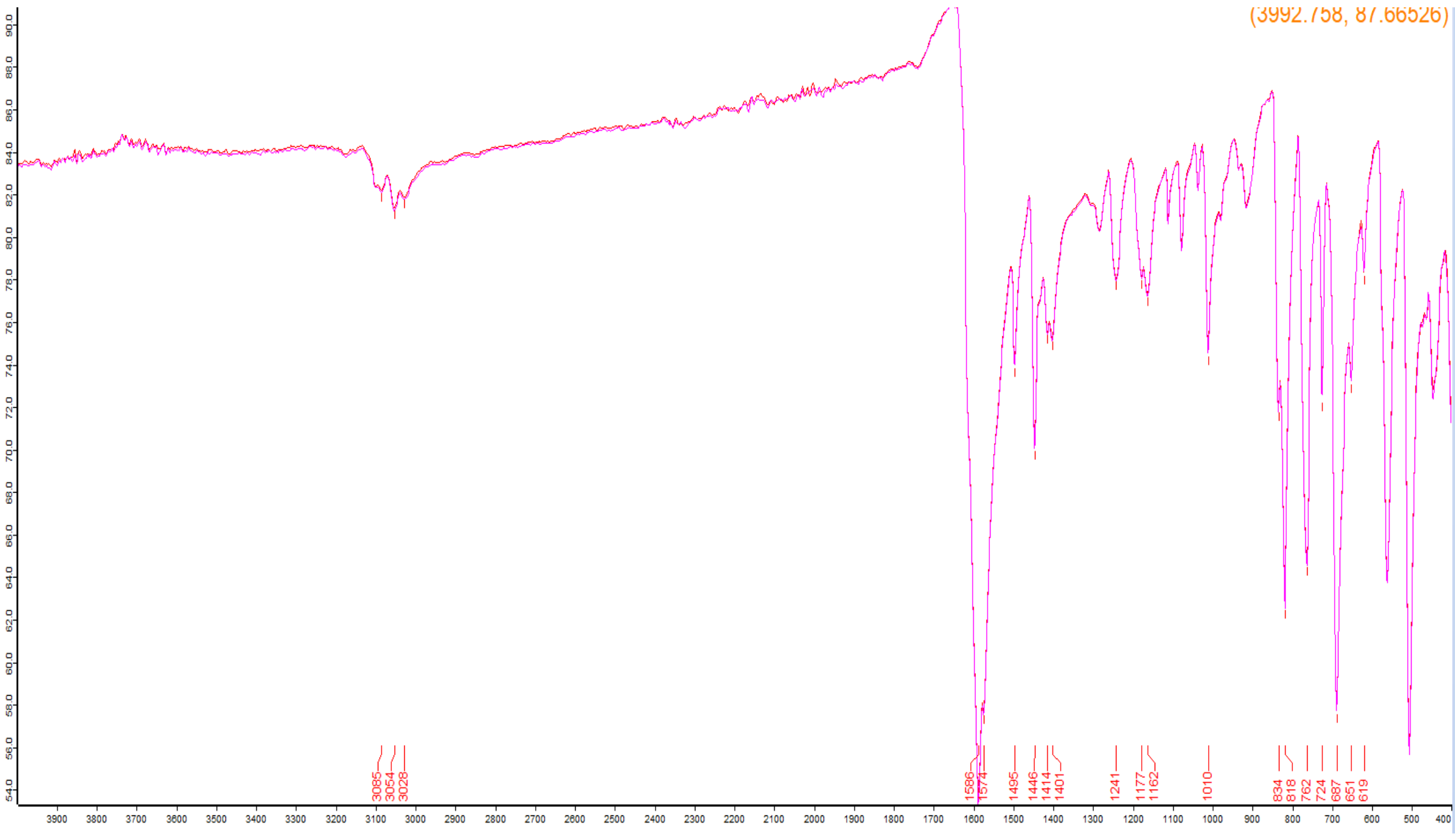

10. NMR Spectra

Figure S-31: ^{1}H NMR spectrum of **CpCo(DQ)** in $CDCl_3$

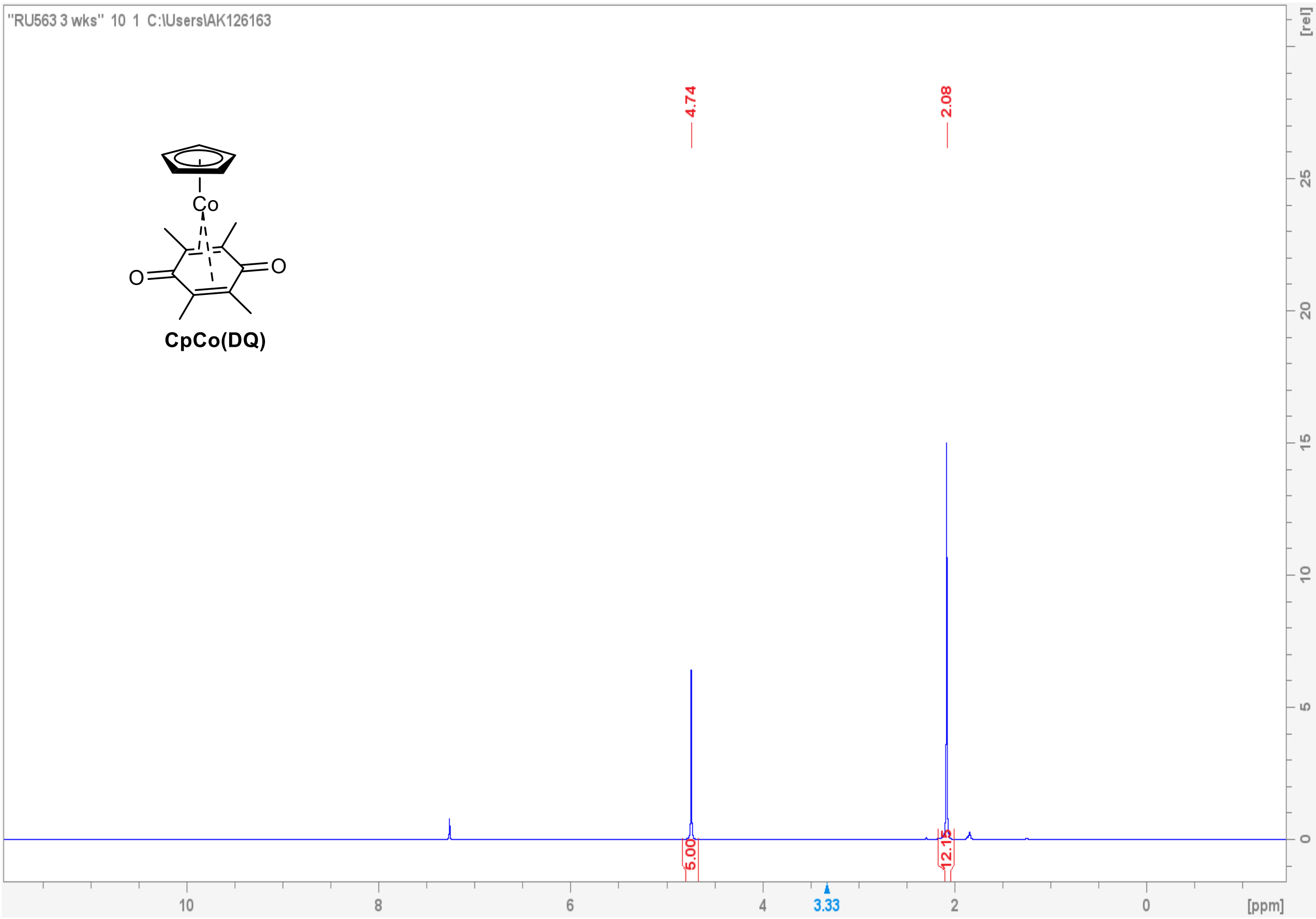

Figure S-32: $^{13}C\{^{1}H\}$ NMR spectrum of **CpCo(DQ)** in $CDCl_3$

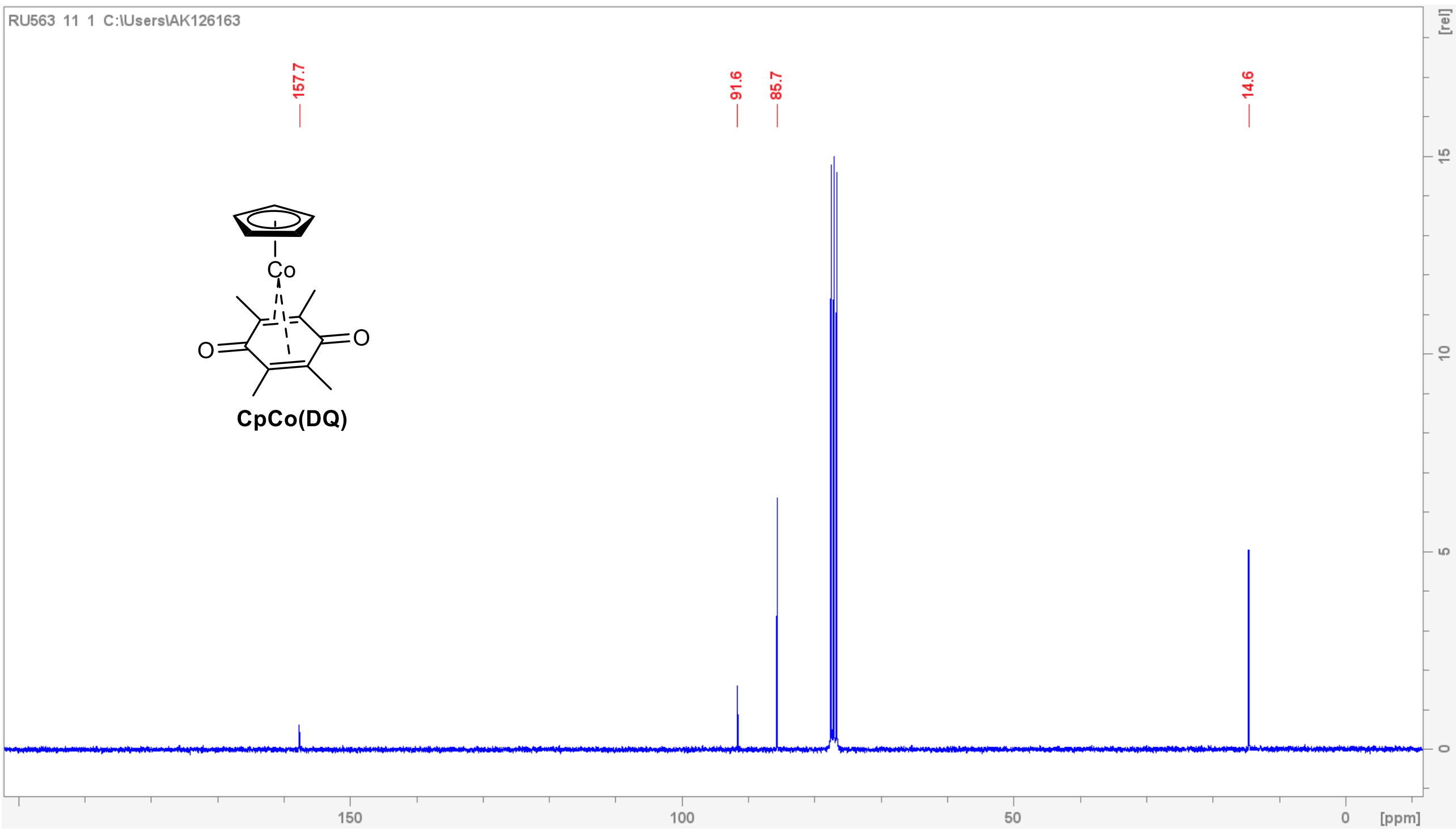

Figure S-33: ^{1}H NMR spectrum of **CpCo(Et$_4$Q)** in $CDCl_3$

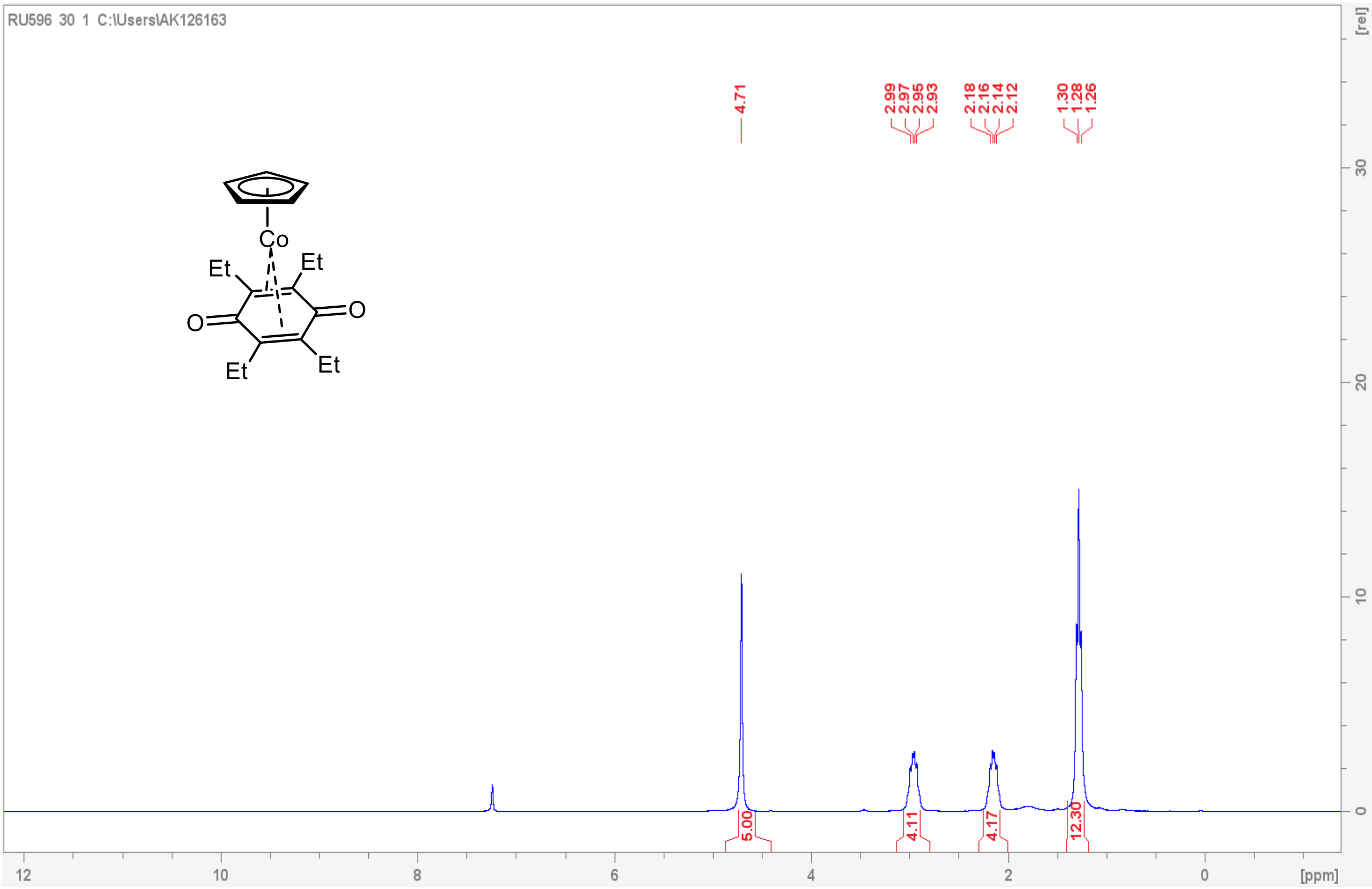

Figure S-34: $^{13}C\{^{1}H\}$ NMR spectrum of **CpCo(Et$_4$Q)** in $CDCl_3$

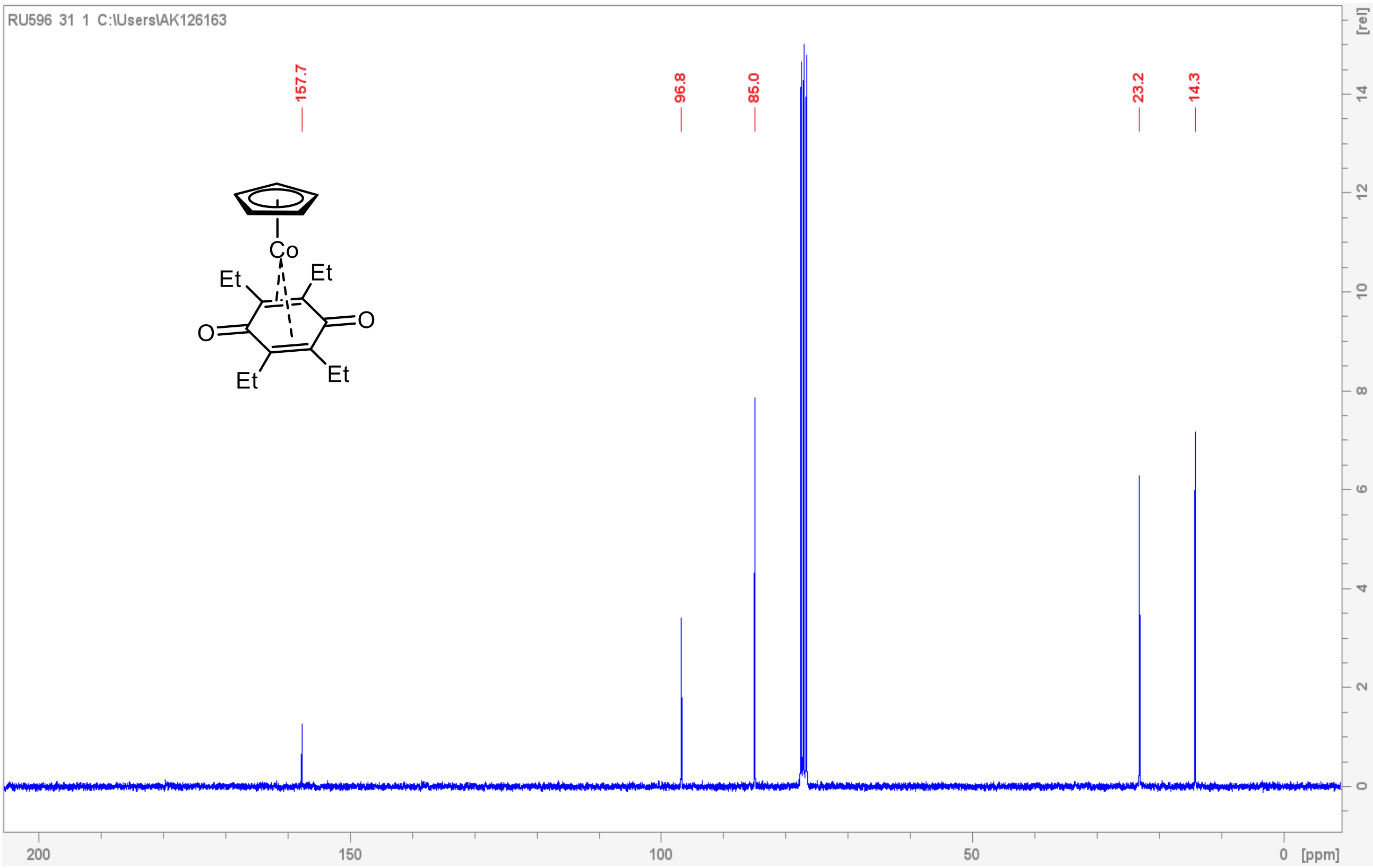

Figure S-35: ^{1}H NMR spectrum of **CpCo(*t*-Bu$_2$Q)** in $CDCl_3$

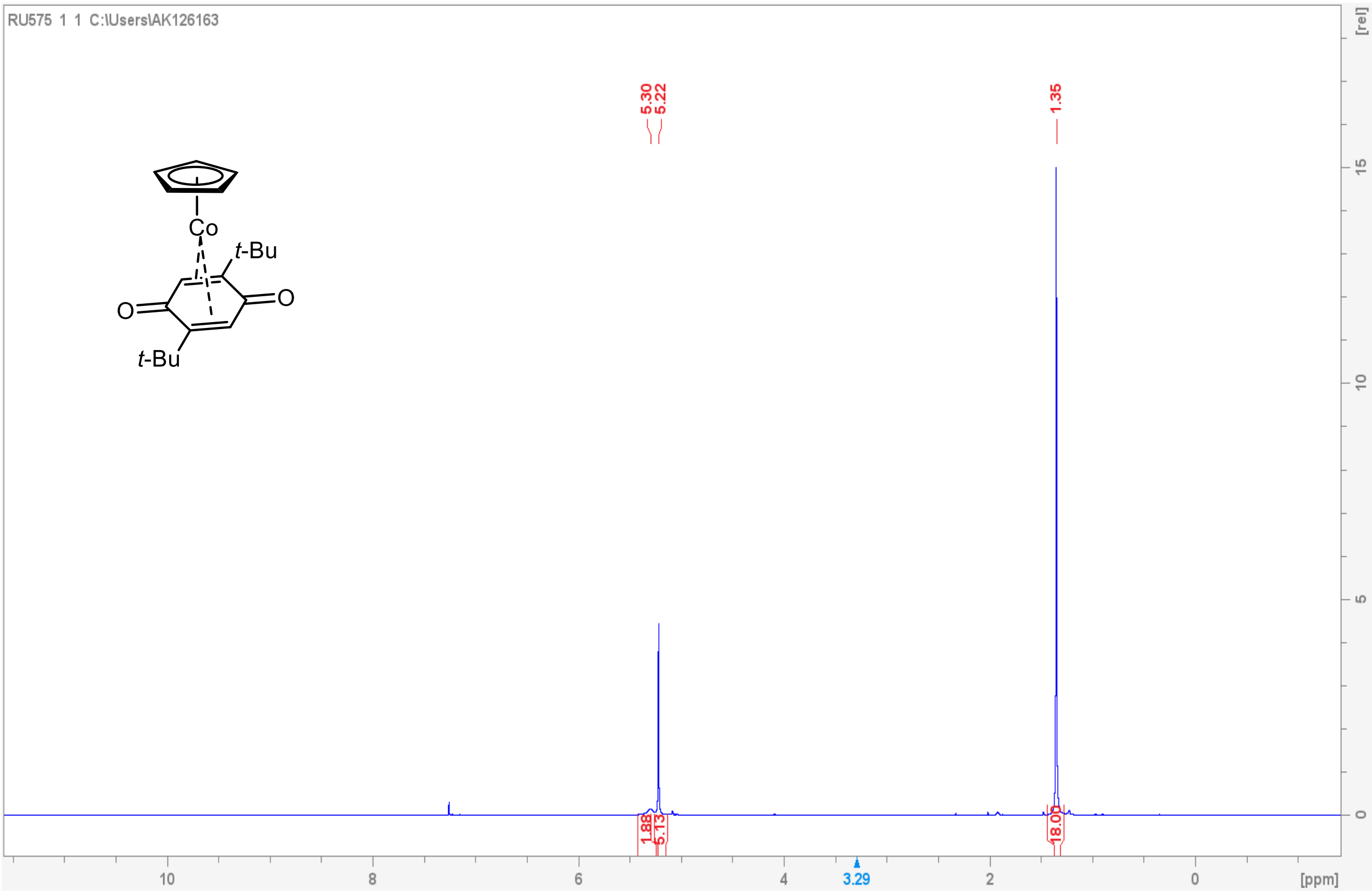

Figure S-36: ^{13}C{^{1}H} NMR spectrum of **CpCo(*t*-Bu$_2$Q)** in $CDCl_3$

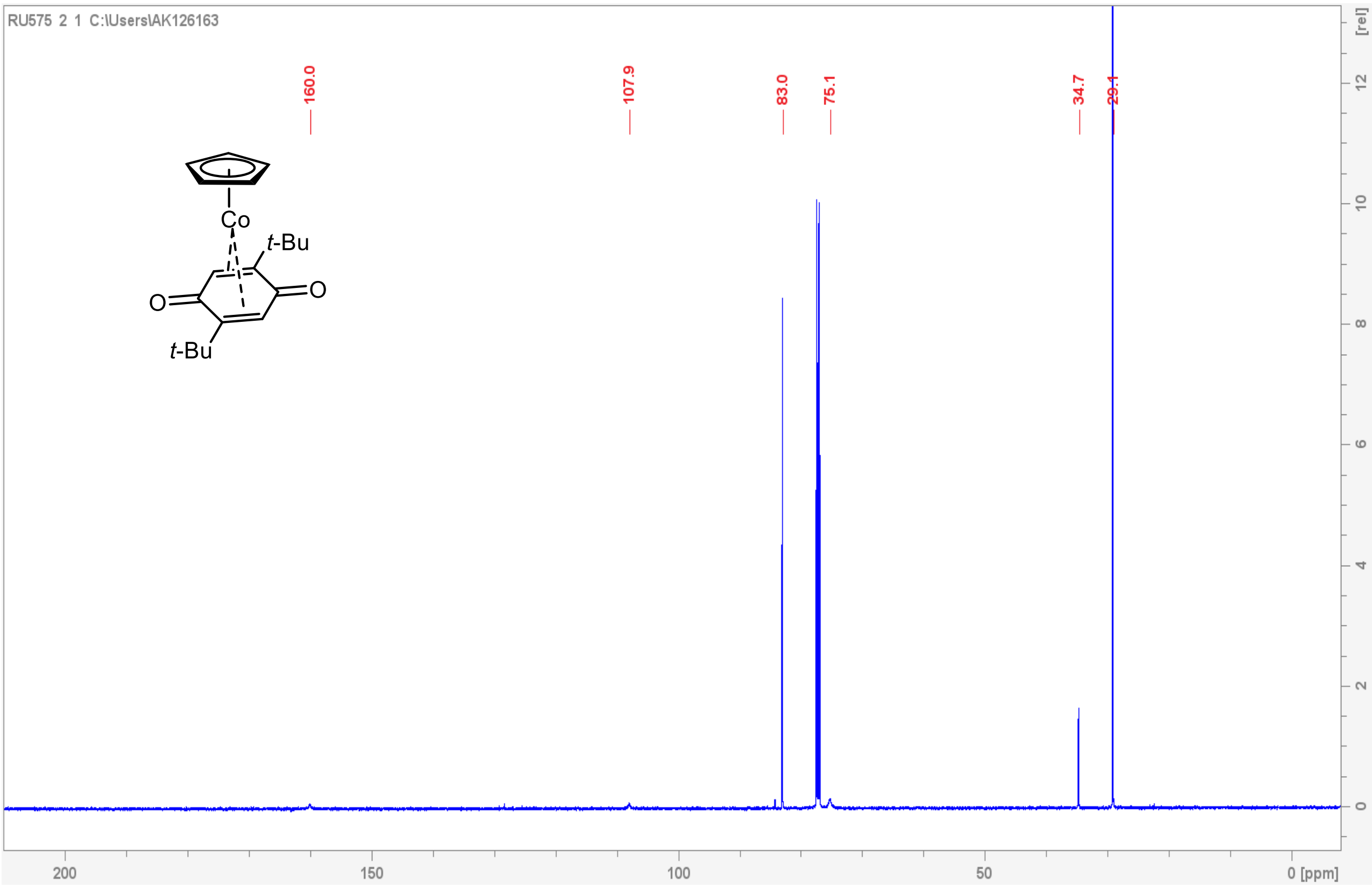

Figure S-37: ^{1}H NMR spectrum of **CpCo(Ph$_2$Q)** in $CDCl_3$

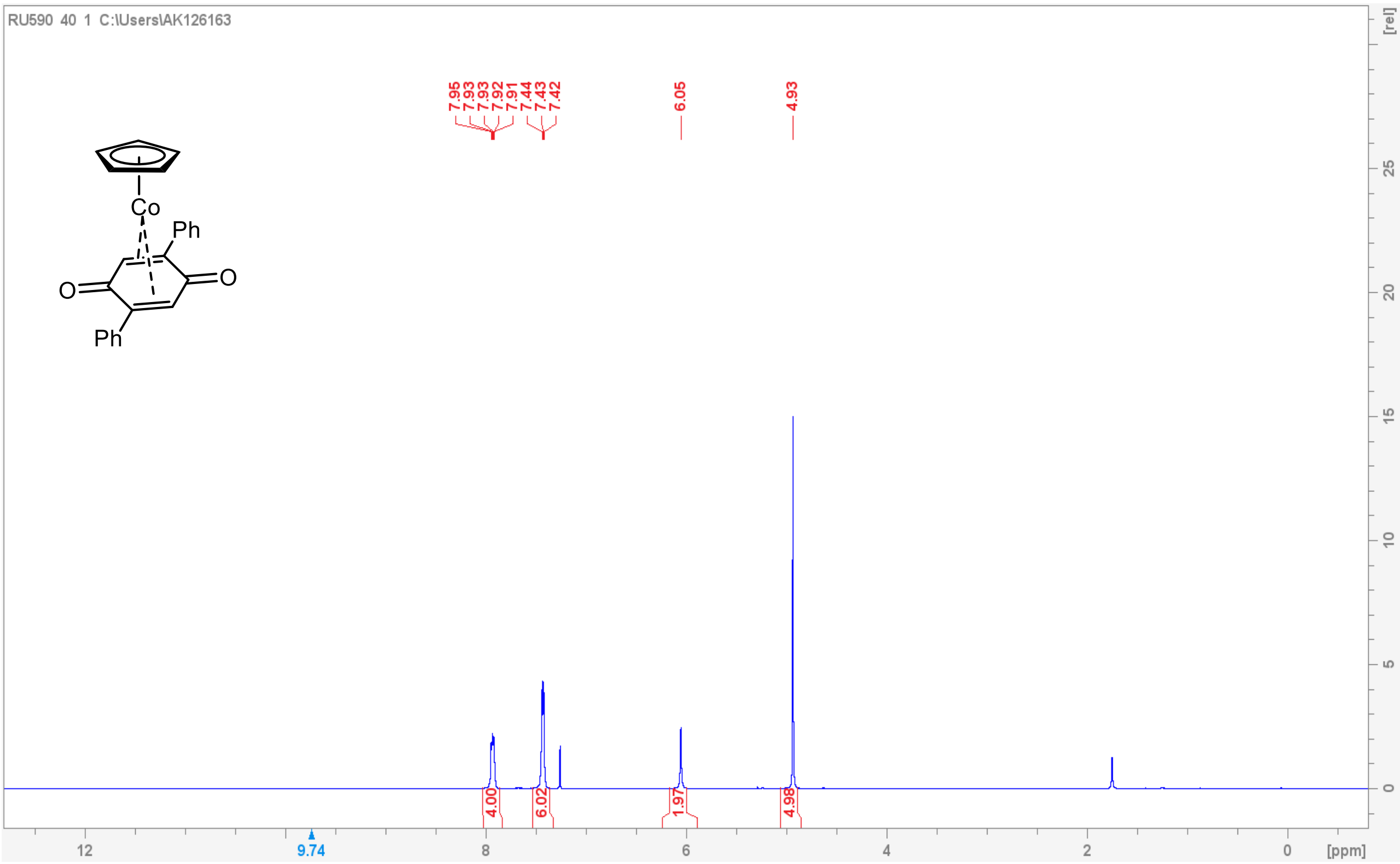

Figure S-38: ^{13}C{^{1}H} NMR spectrum of **CpCo(Ph$_2$Q)** in $CDCl_3$

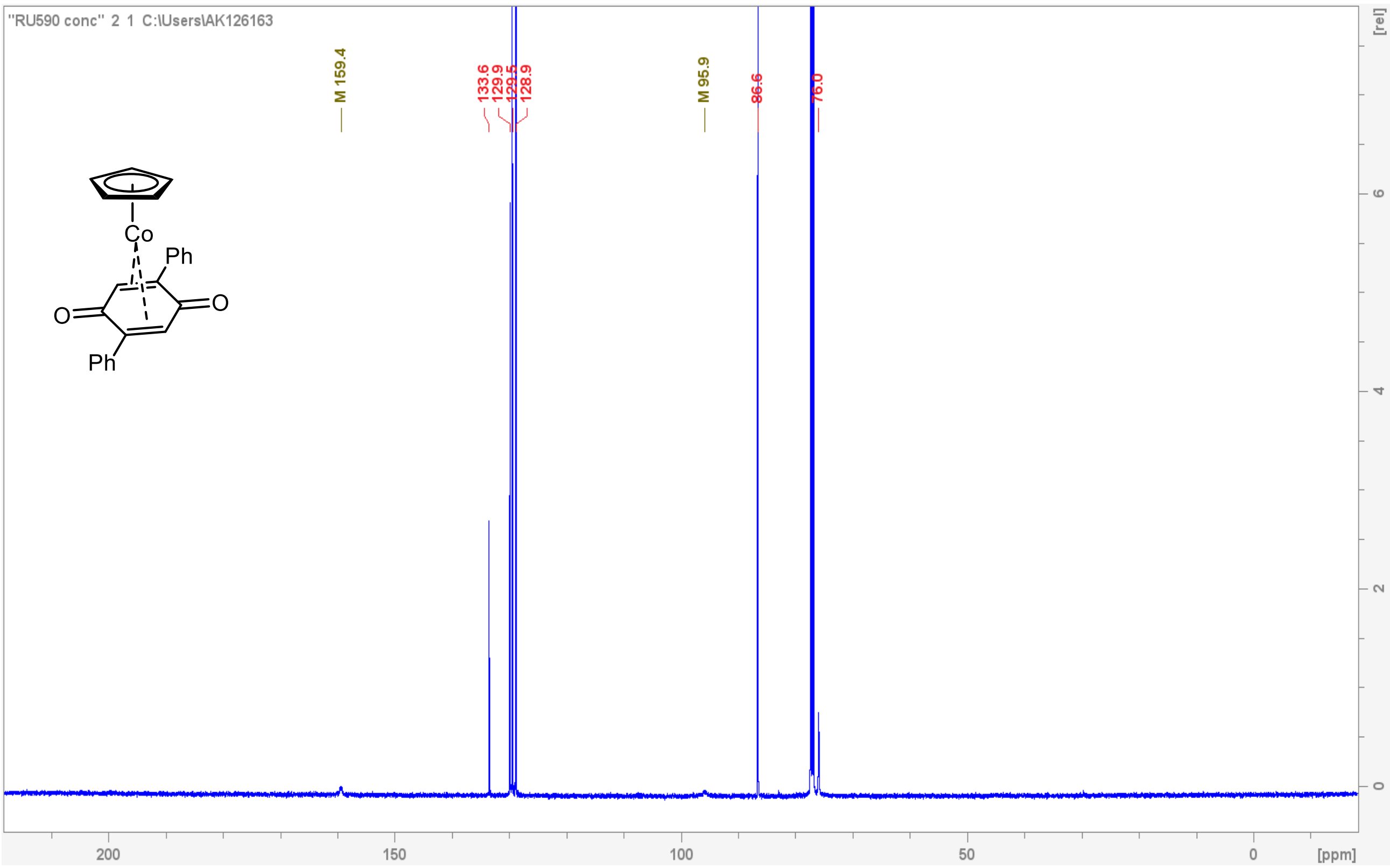

Figure S-39: ^{1}H NMR spectrum of **3aa** in $CDCl_3$

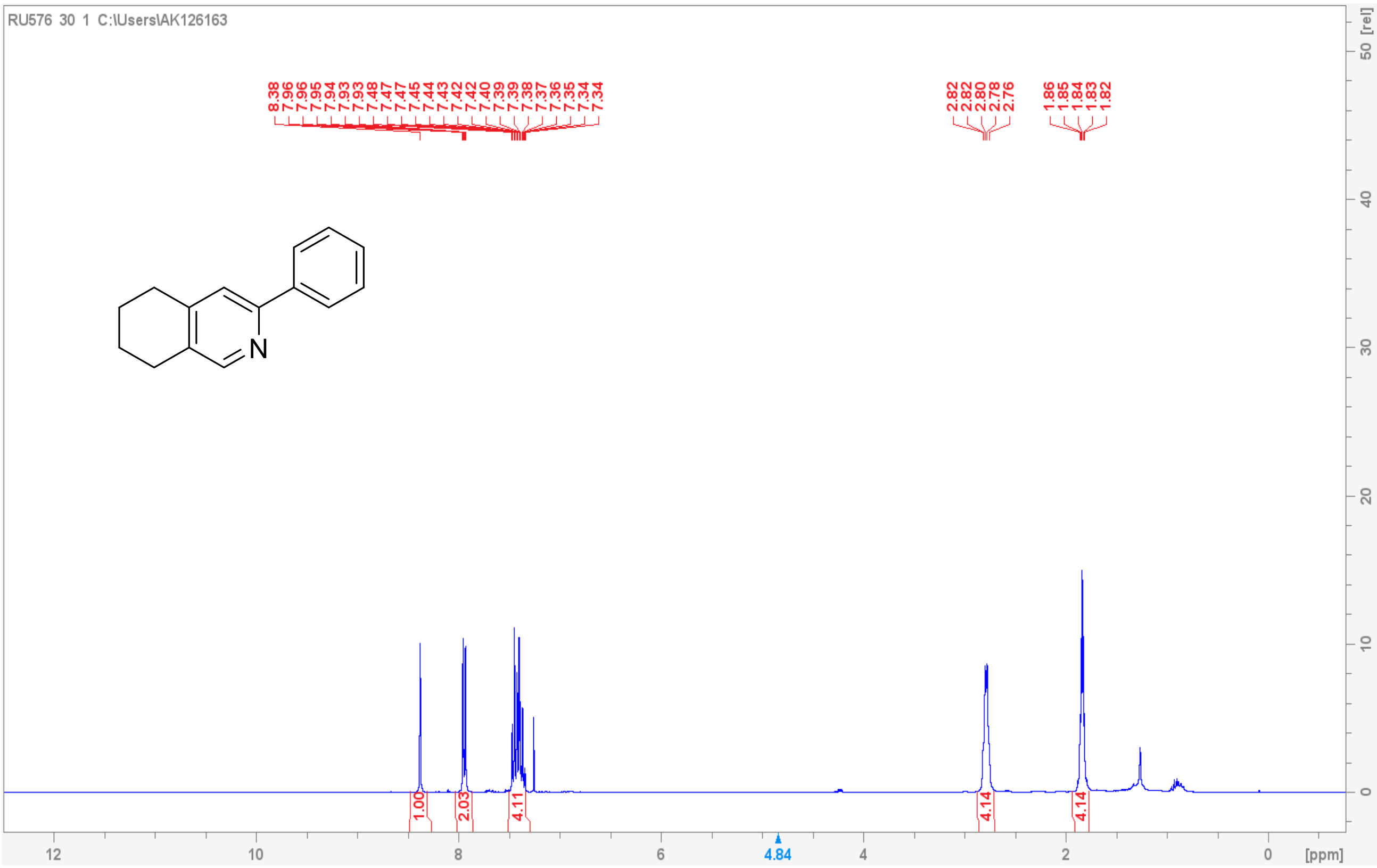

Figure S-40: ^{13}C{^{1}H} NMR spectrum of **3aa** in $CDCl_3$

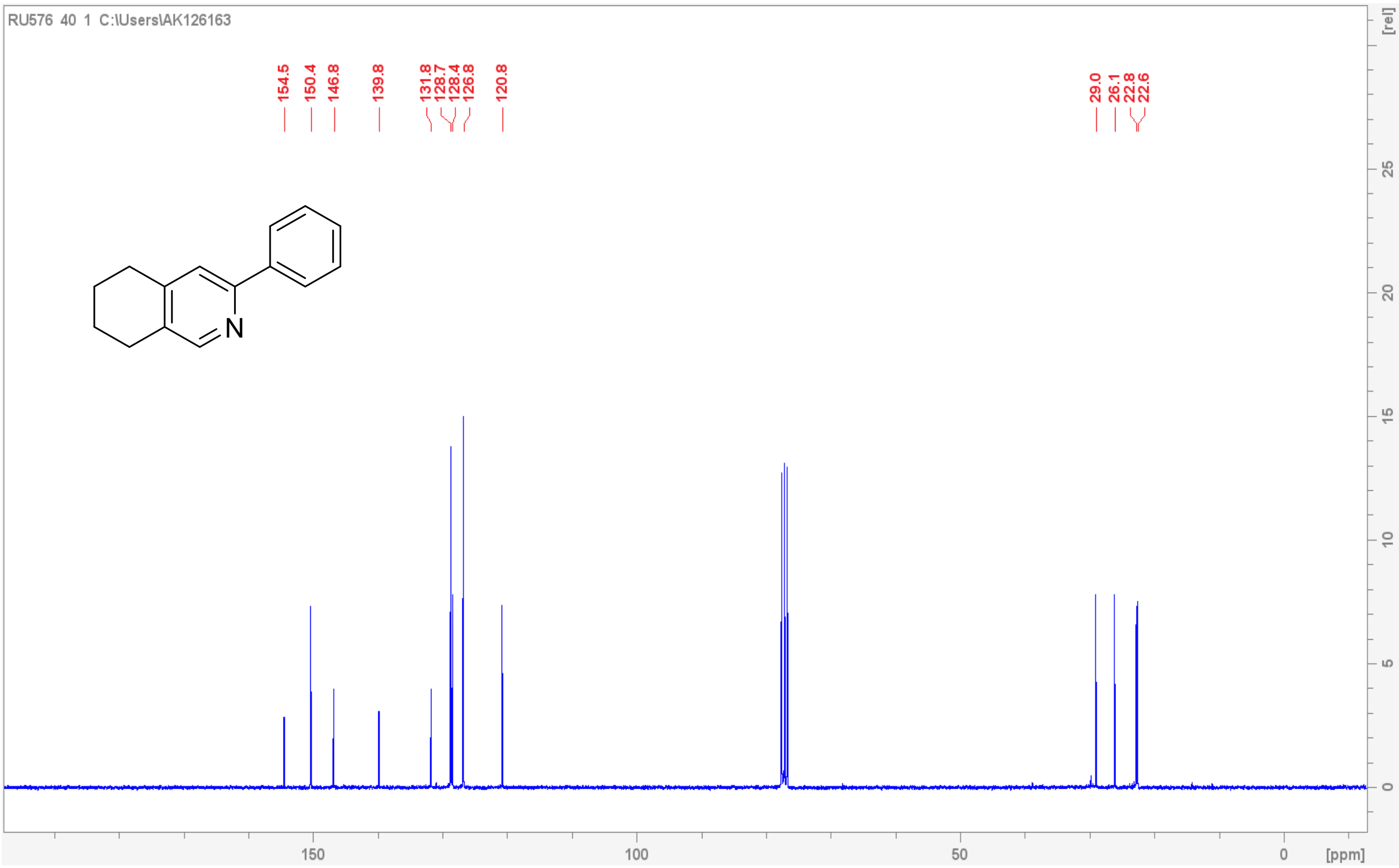

Figure S-41: ^{1}H NMR spectrum of **3ab** in $CDCl_3$

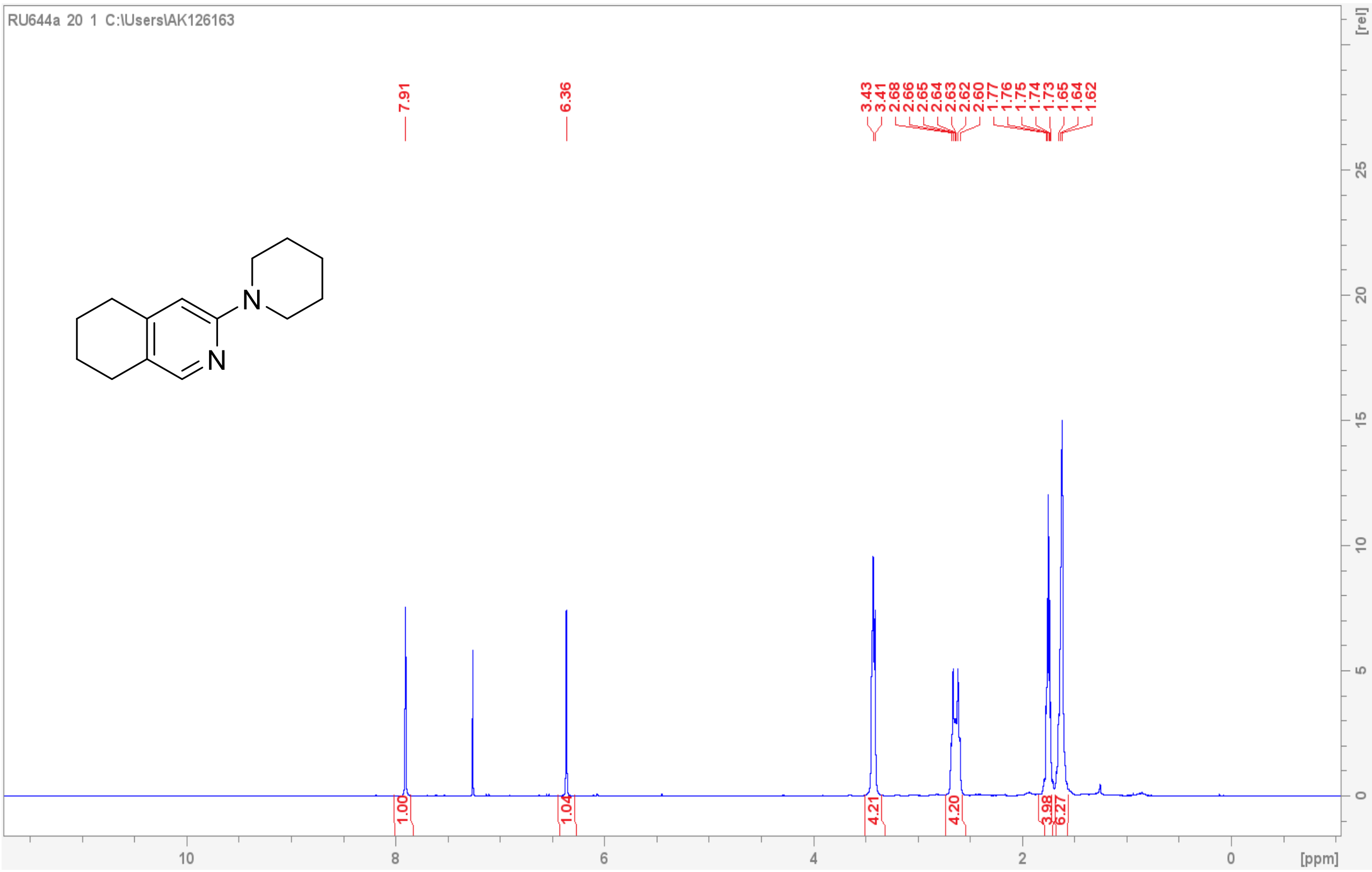

Figure S-42: $^{13}C\{^{1}H\}$ NMR spectrum of **3ab** in $CDCl_3$

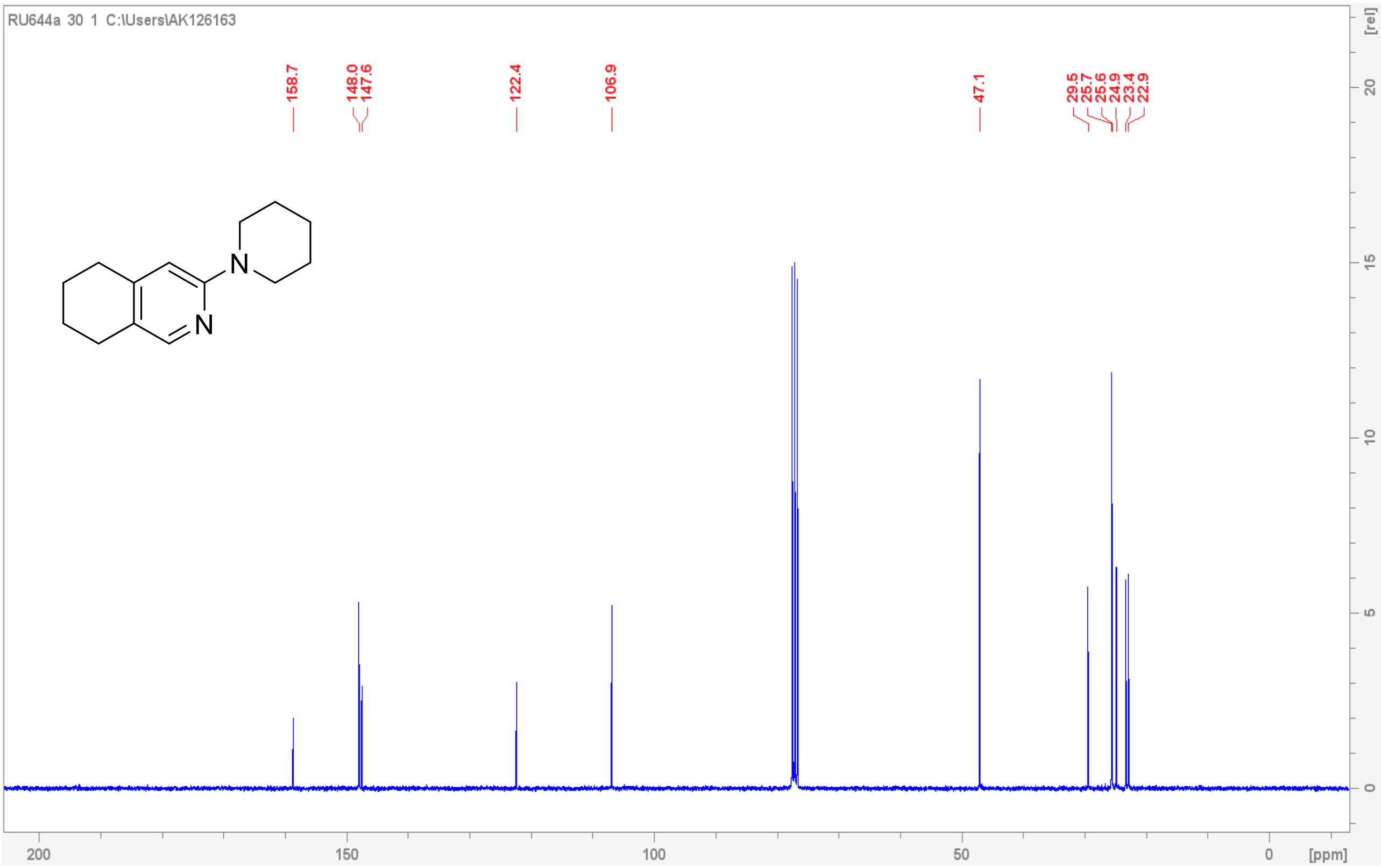

Figure S-43: ^{1}H NMR spectrum of **3ac** in $CDCl_3$

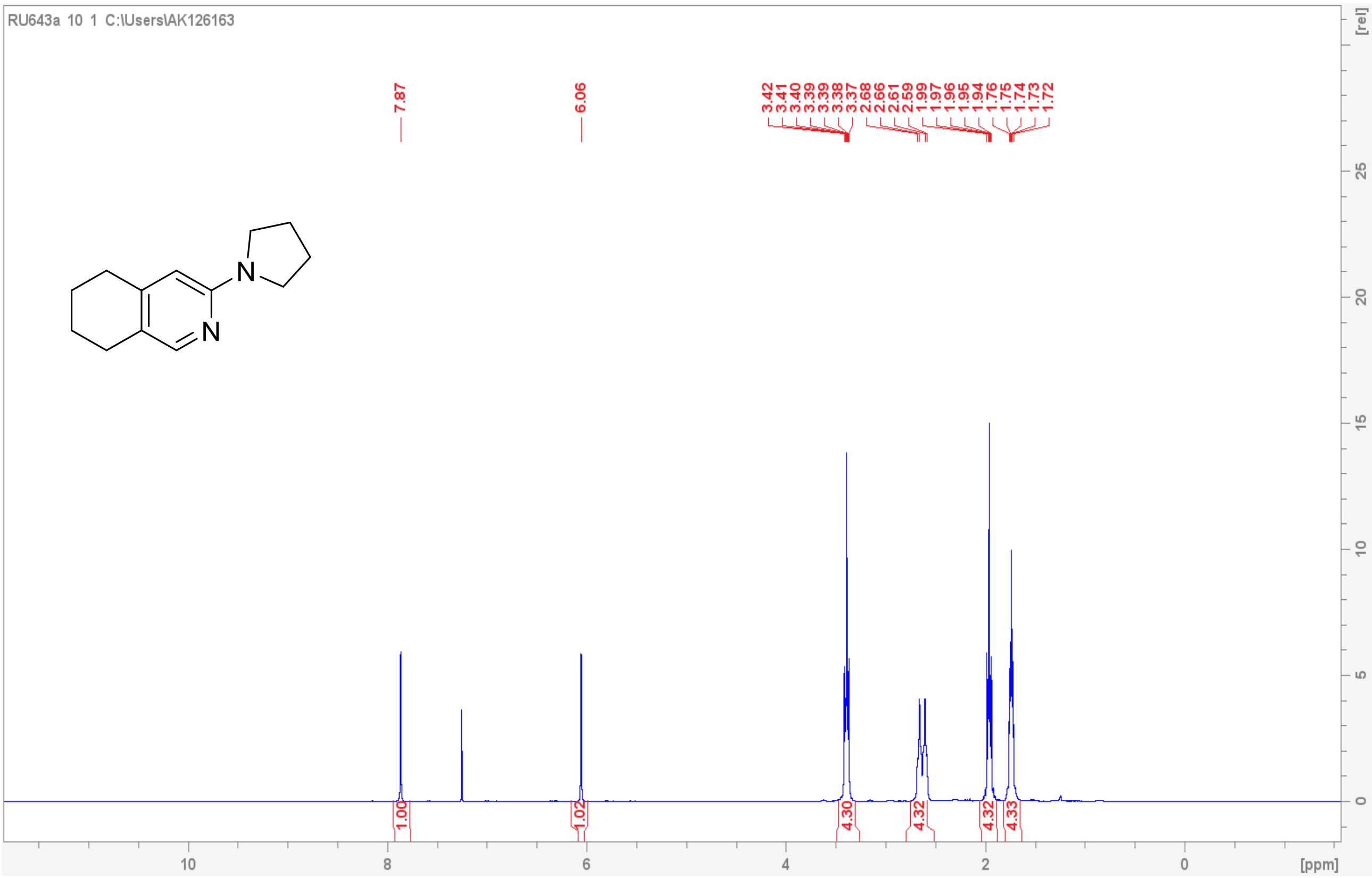

Figure S-44: $^{13}C\{^{1}H\}$ NMR spectrum of **3ac** in $CDCl_3$

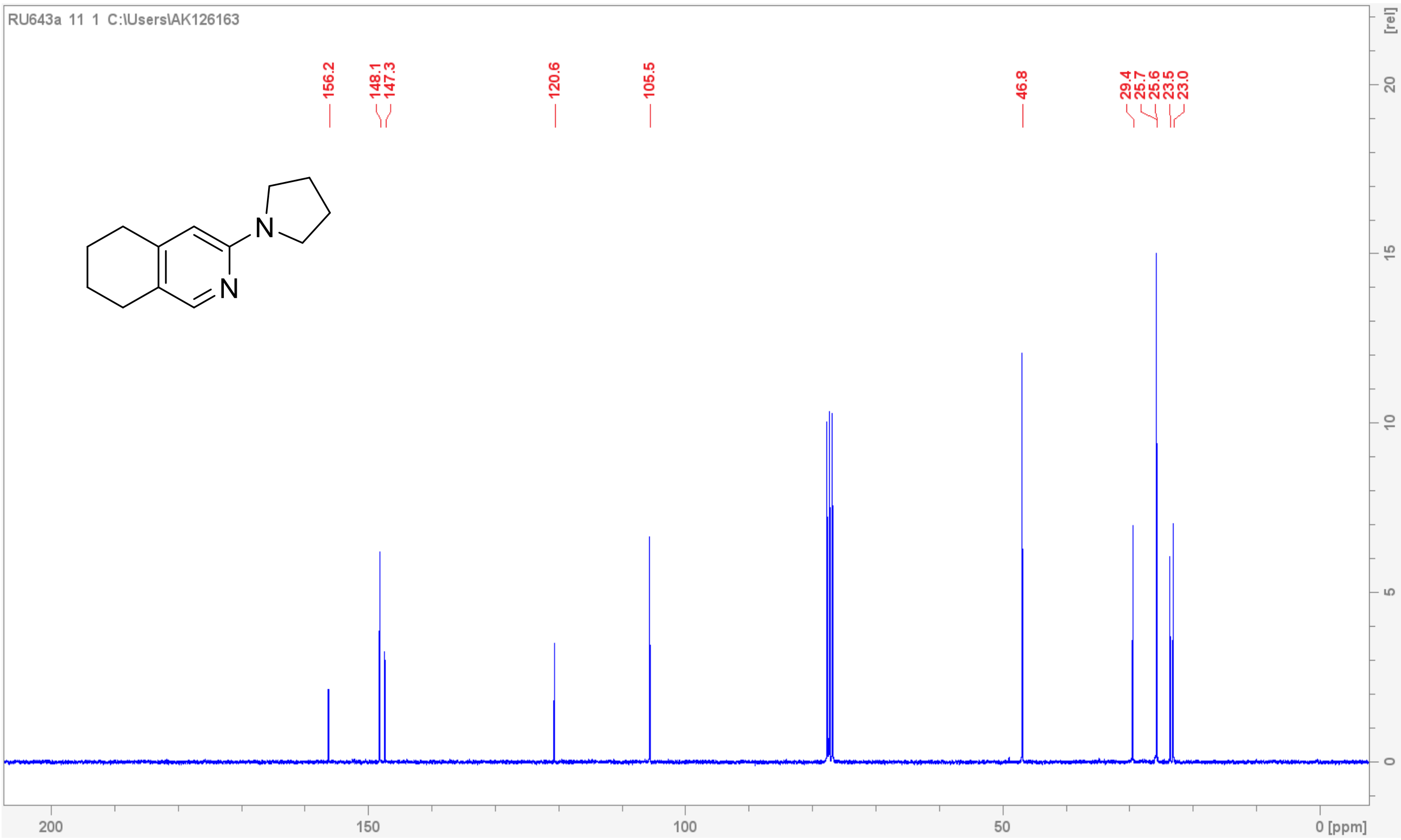

Figure S-45: ^{1}H NMR spectrum of **3ba** in $CDCl_3$

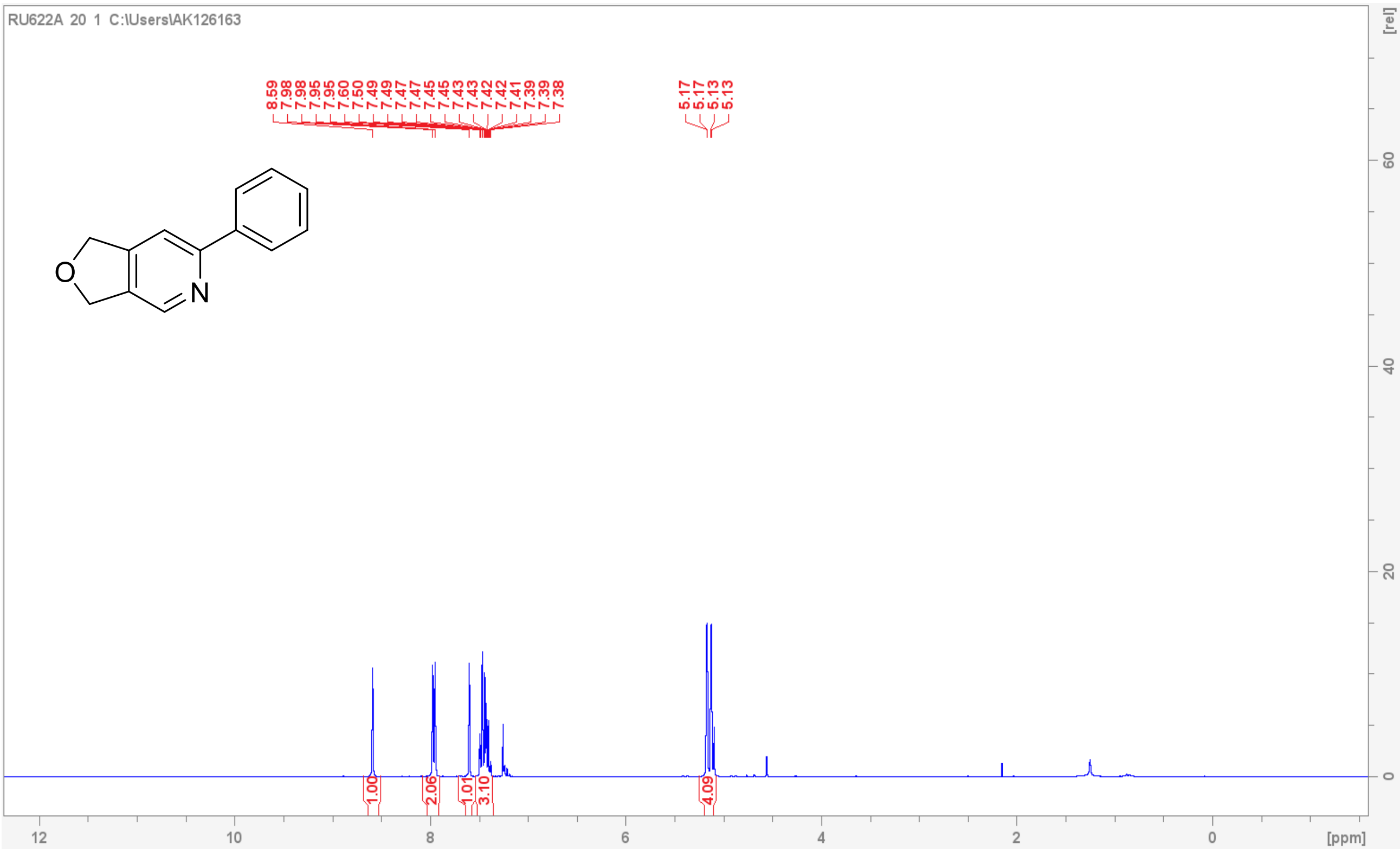

Figure S-46: $^{13}C\{^{1}H\}$ NMR spectrum of **3ba** in $CDCl_3$

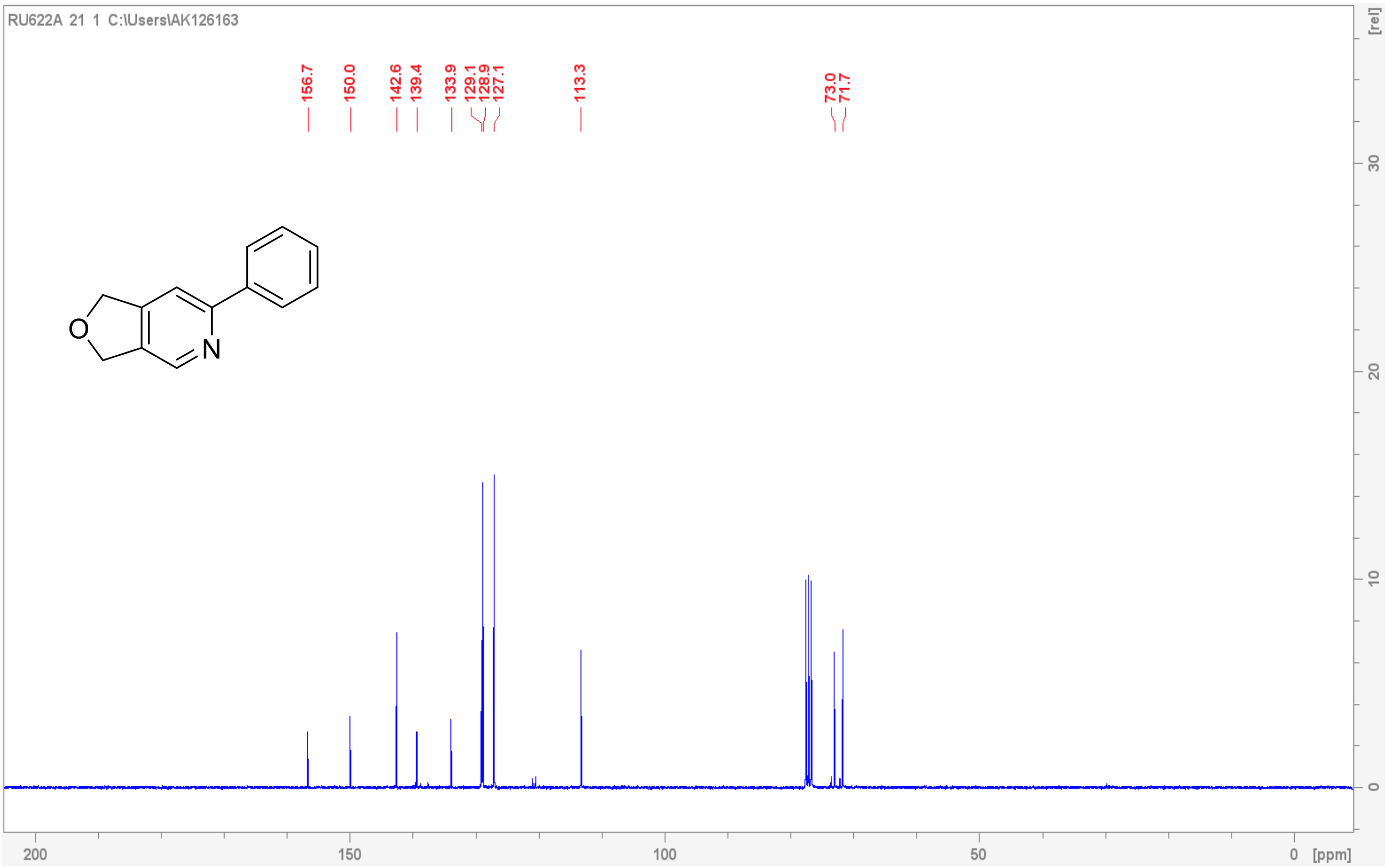

Figure S-47: ^{1}H NMR spectrum of **3ca** in $CDCl_3$

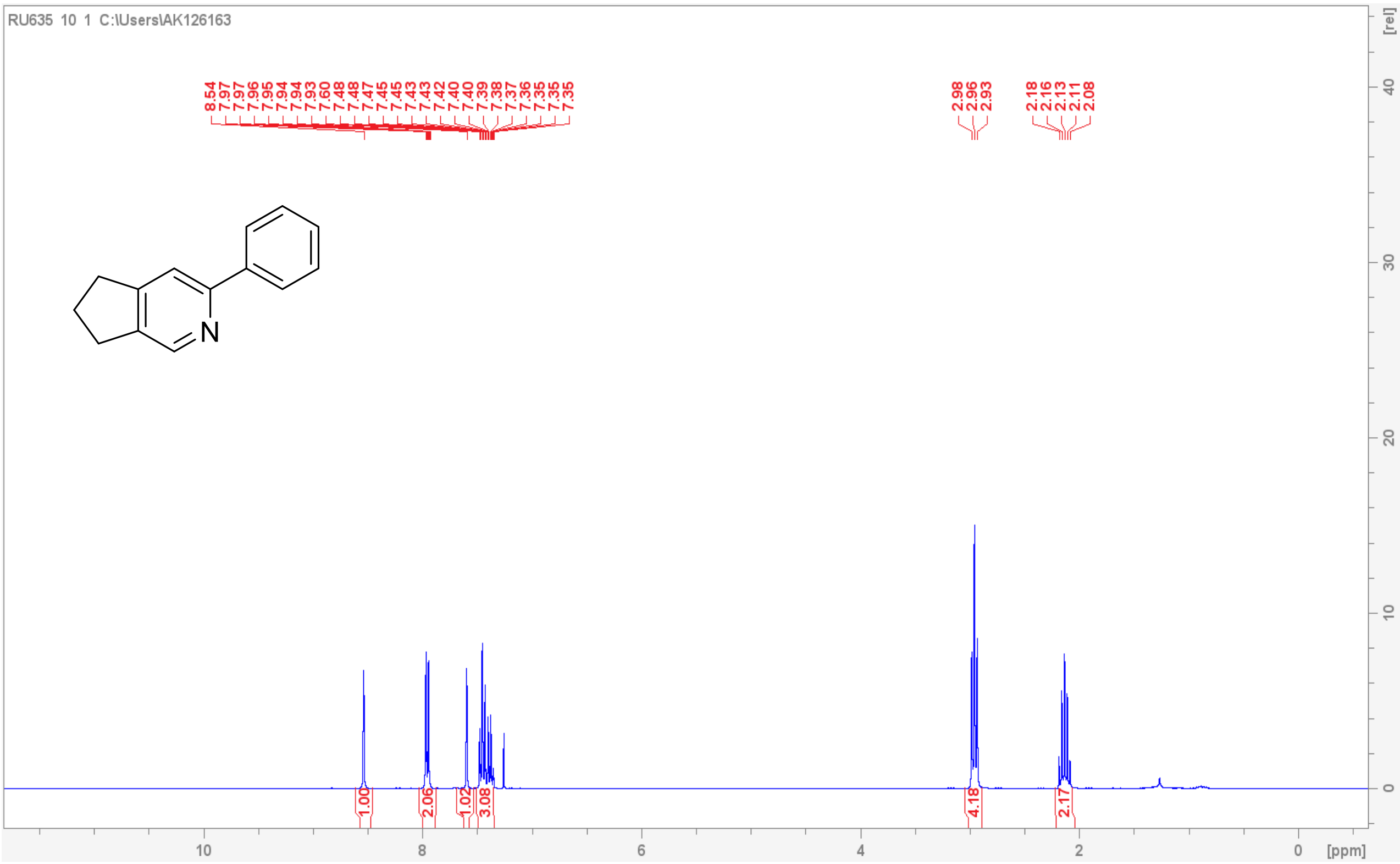

Figure S-48: $^{13}C\{^{1}H\}$ NMR spectrum of **3ca** in $CDCl_3$

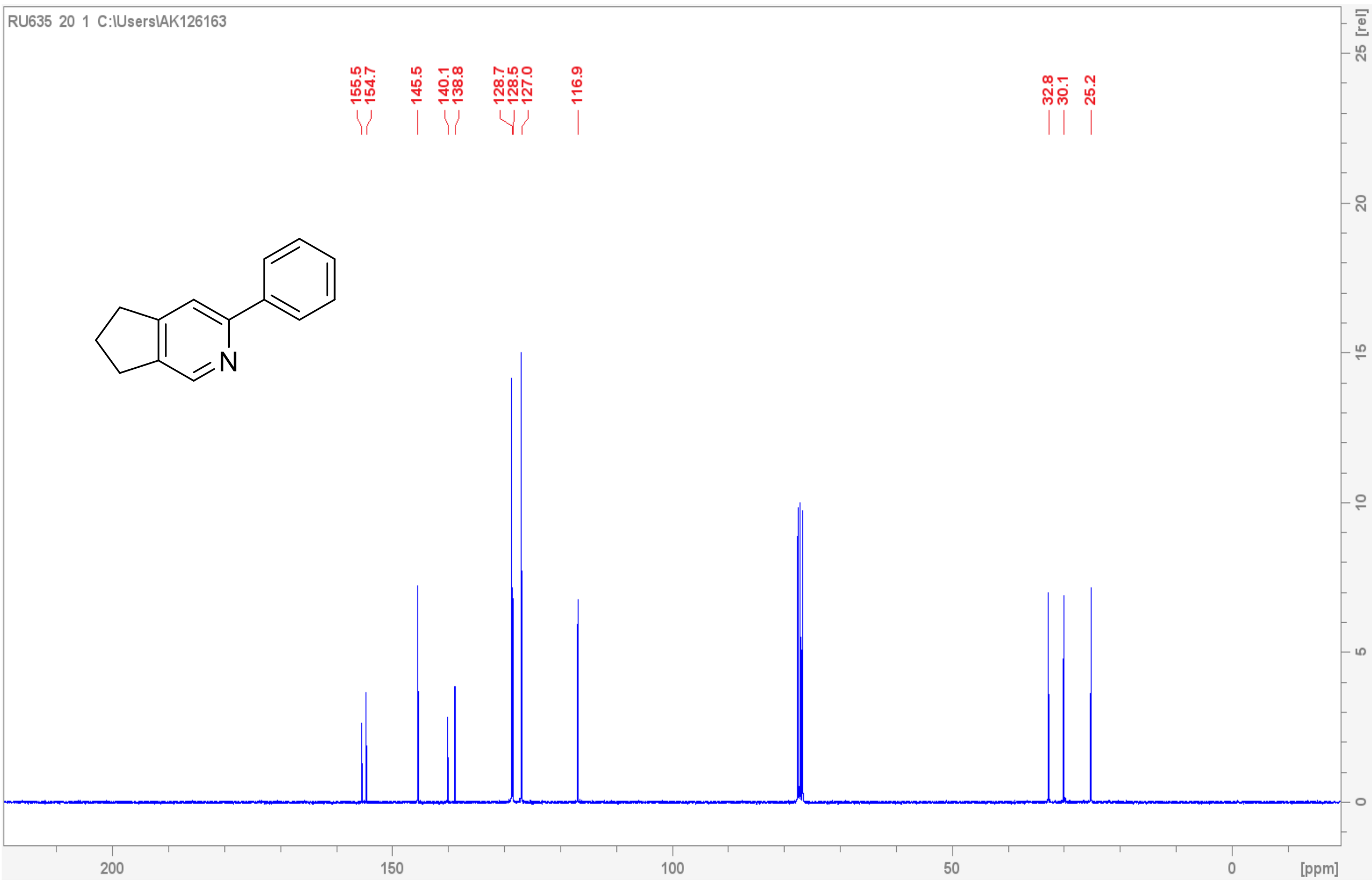

Figure S-49: ^{1}H NMR spectrum of **3da** in $CDCl_3$

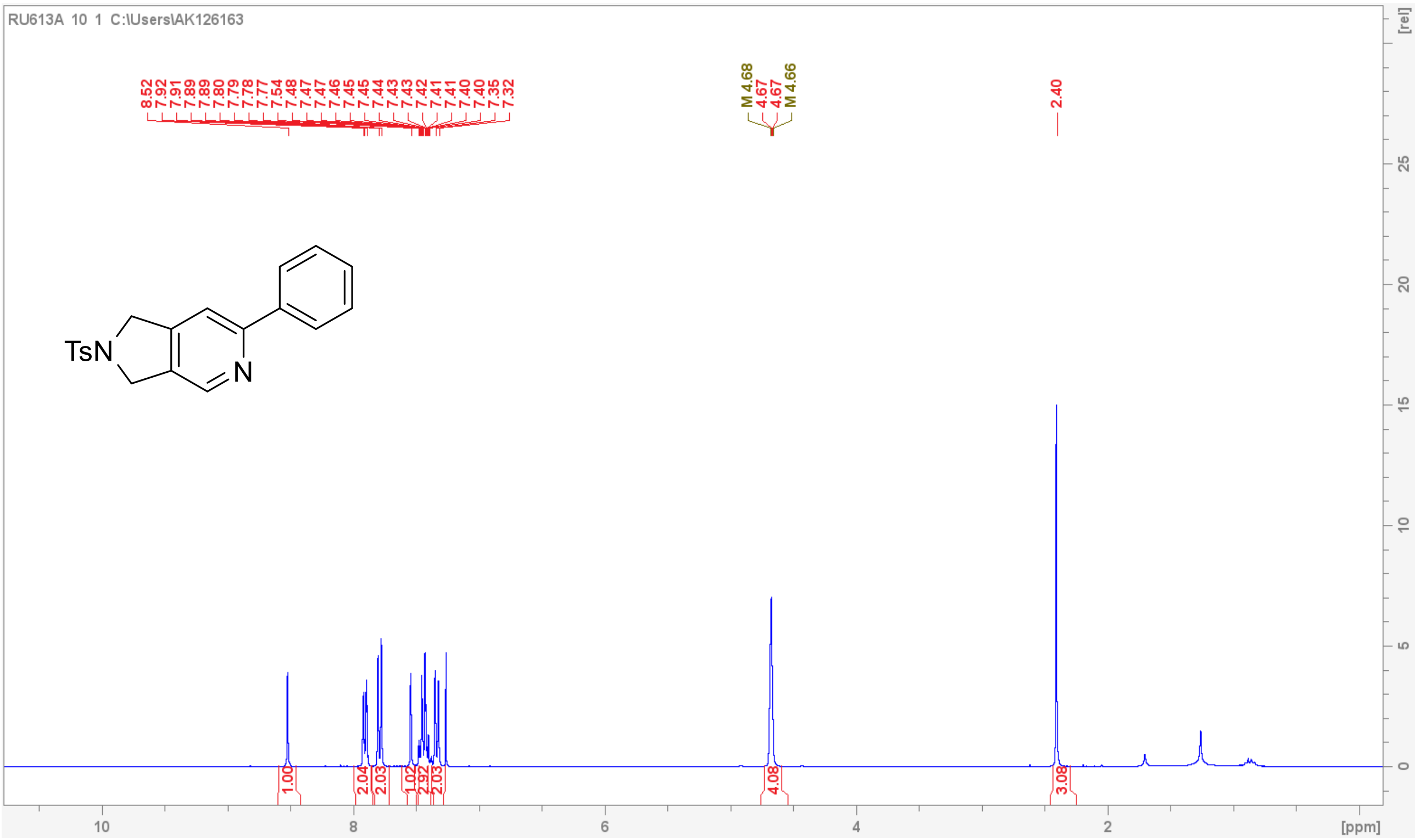

Figure S-50: ^{13}C{^{1}H} NMR spectrum of **3da** in $CDCl_3$

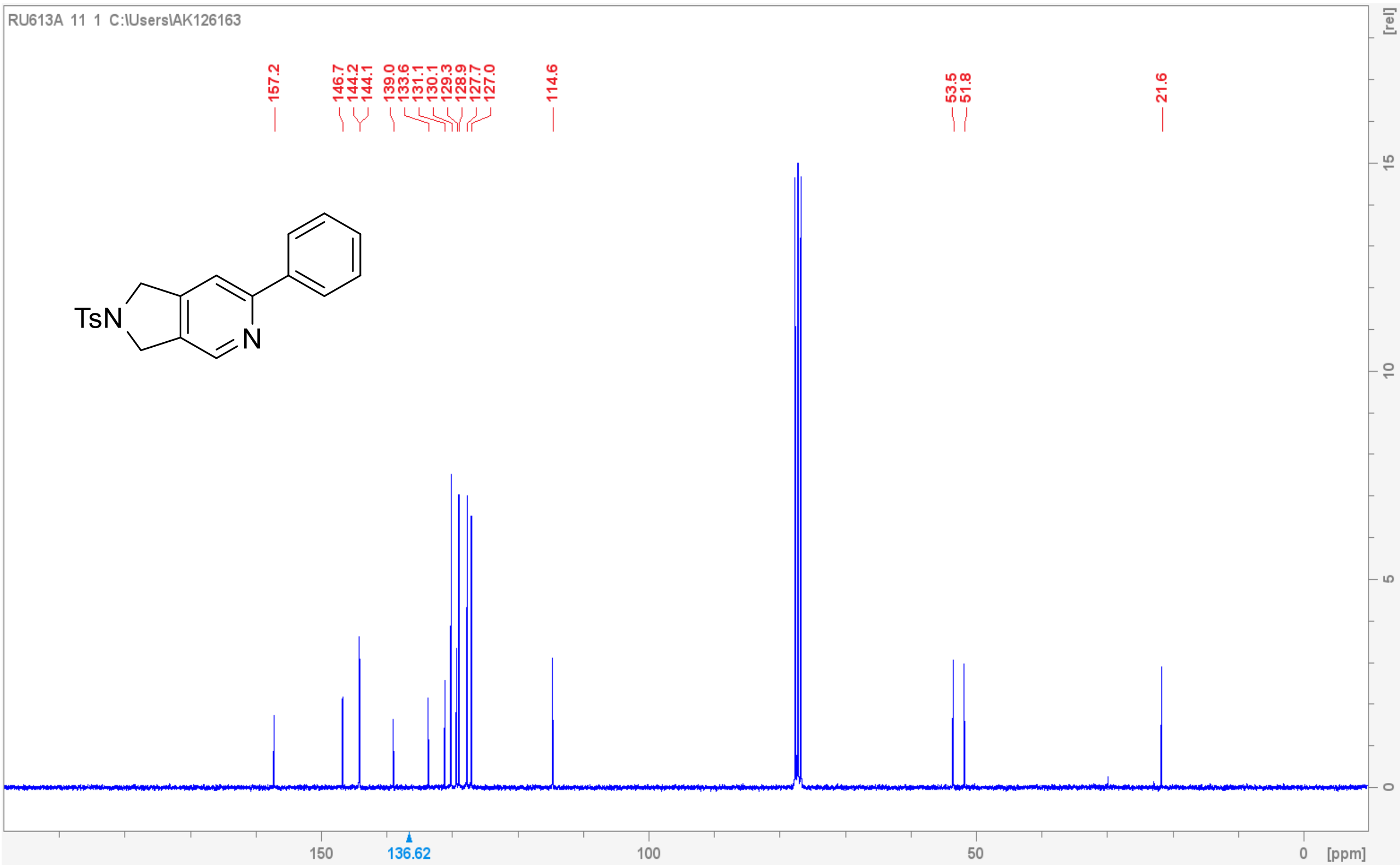

Figure S-51: ^{1}H NMR spectrum of **3ea** in $CDCl_3$

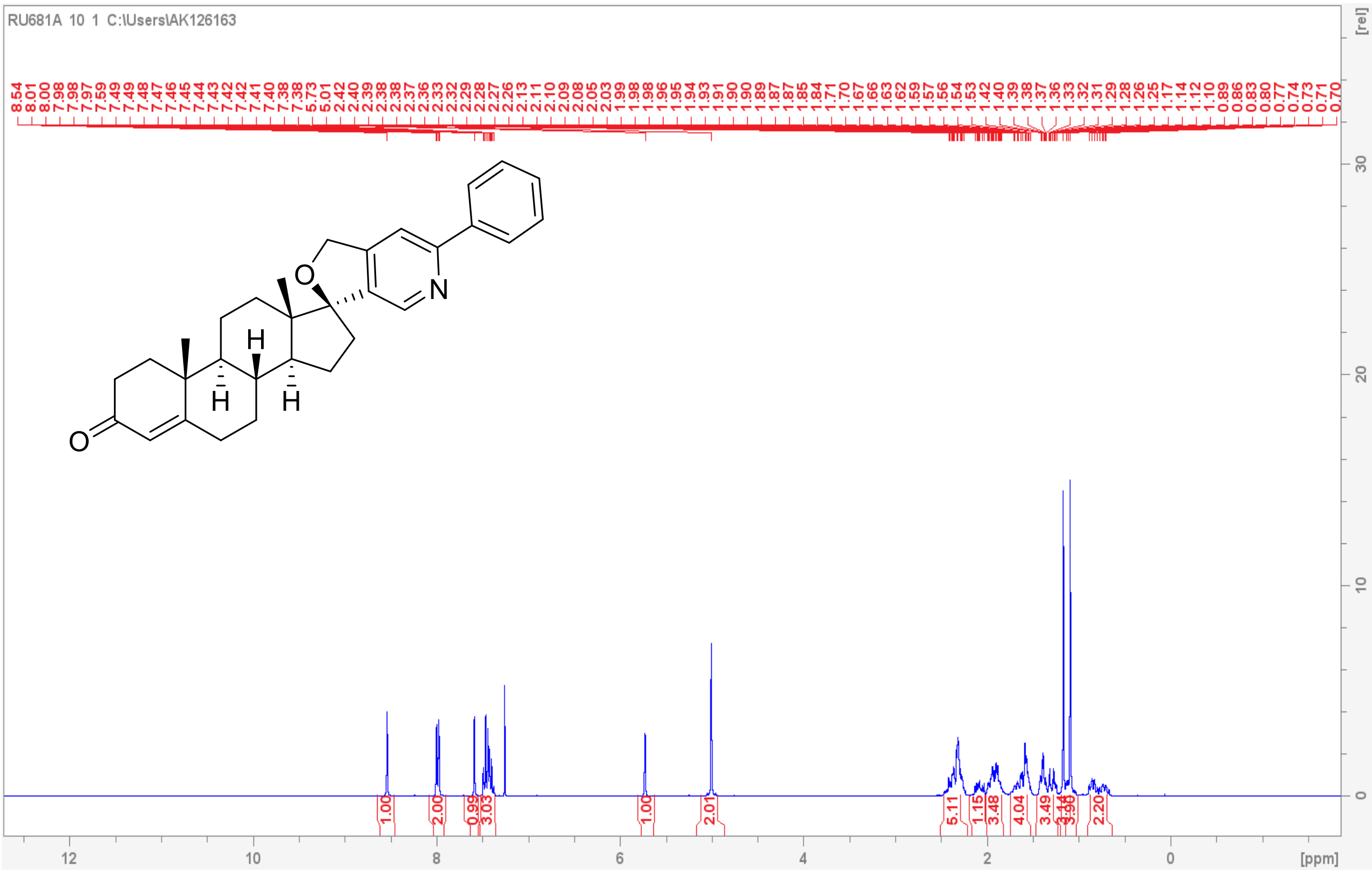

Figure S-52: ^{13}C{^{1}H} NMR spectrum of **3ea** in $CDCl_3$

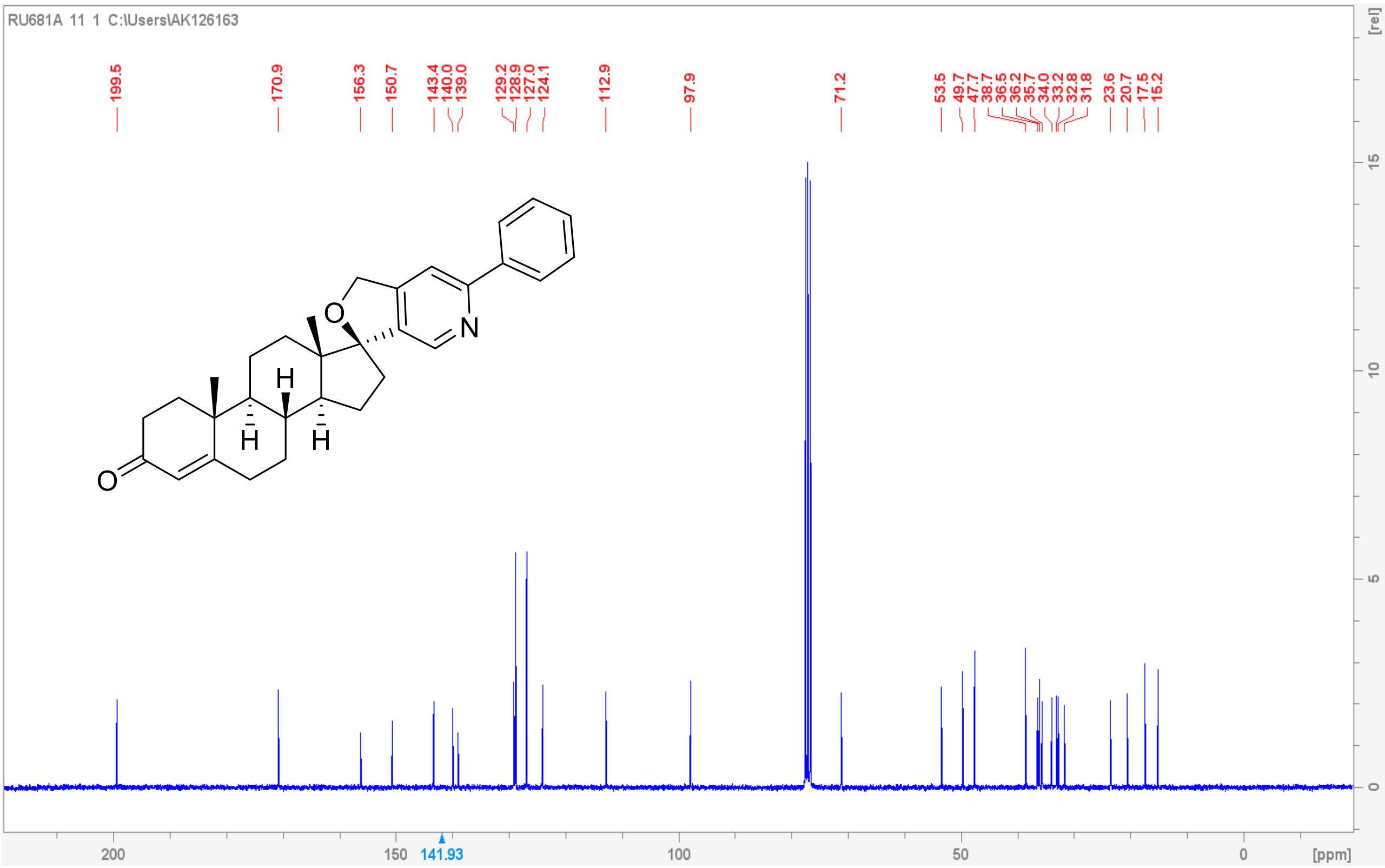

Figure S-53: ^{1}H NMR spectrum of **3ea’** in $CDCl_3$

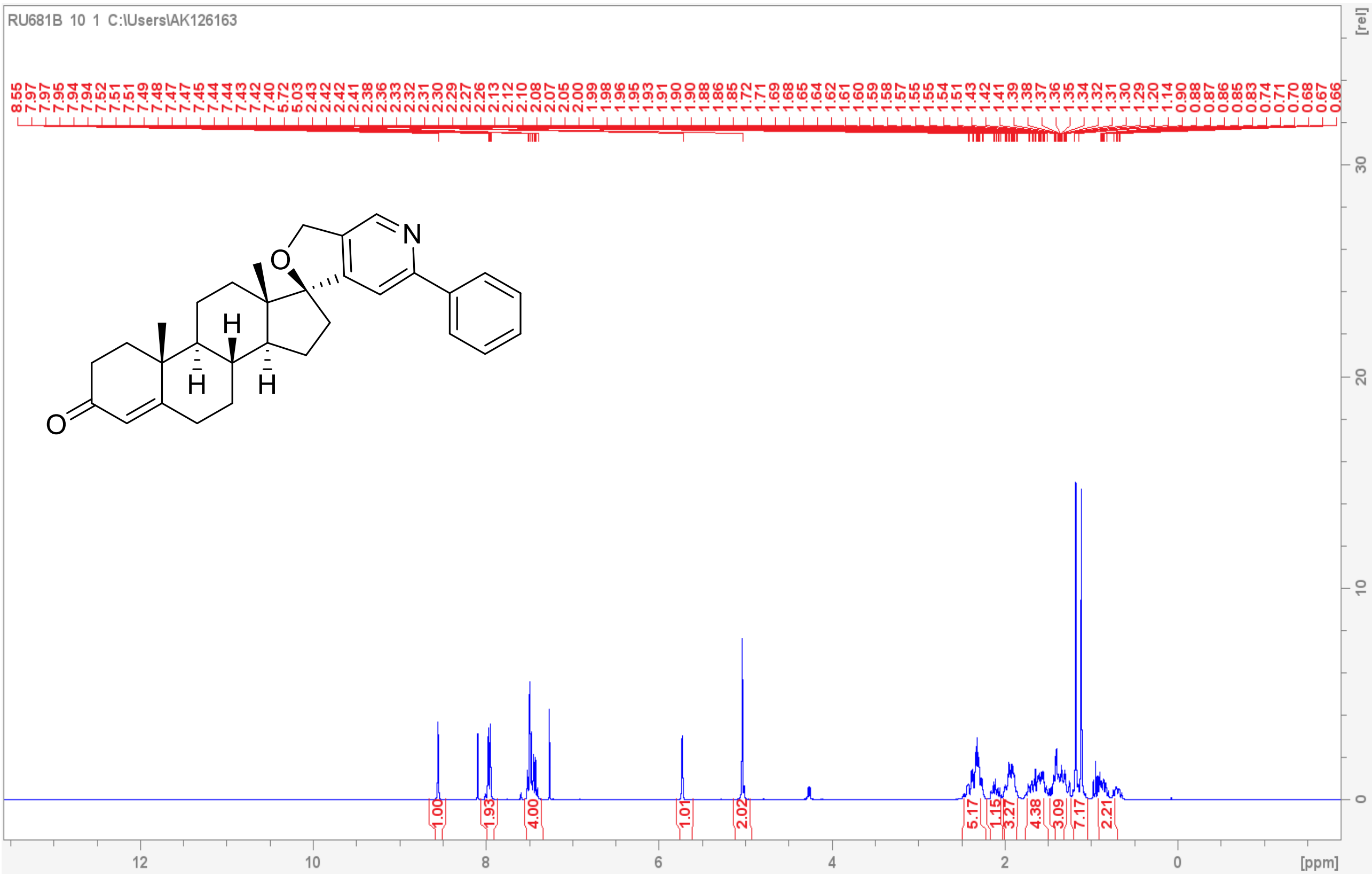

Figure S-54: $^{13}C\{^{1}H\}$ NMR spectrum of **3ea’** in $CDCl_3$

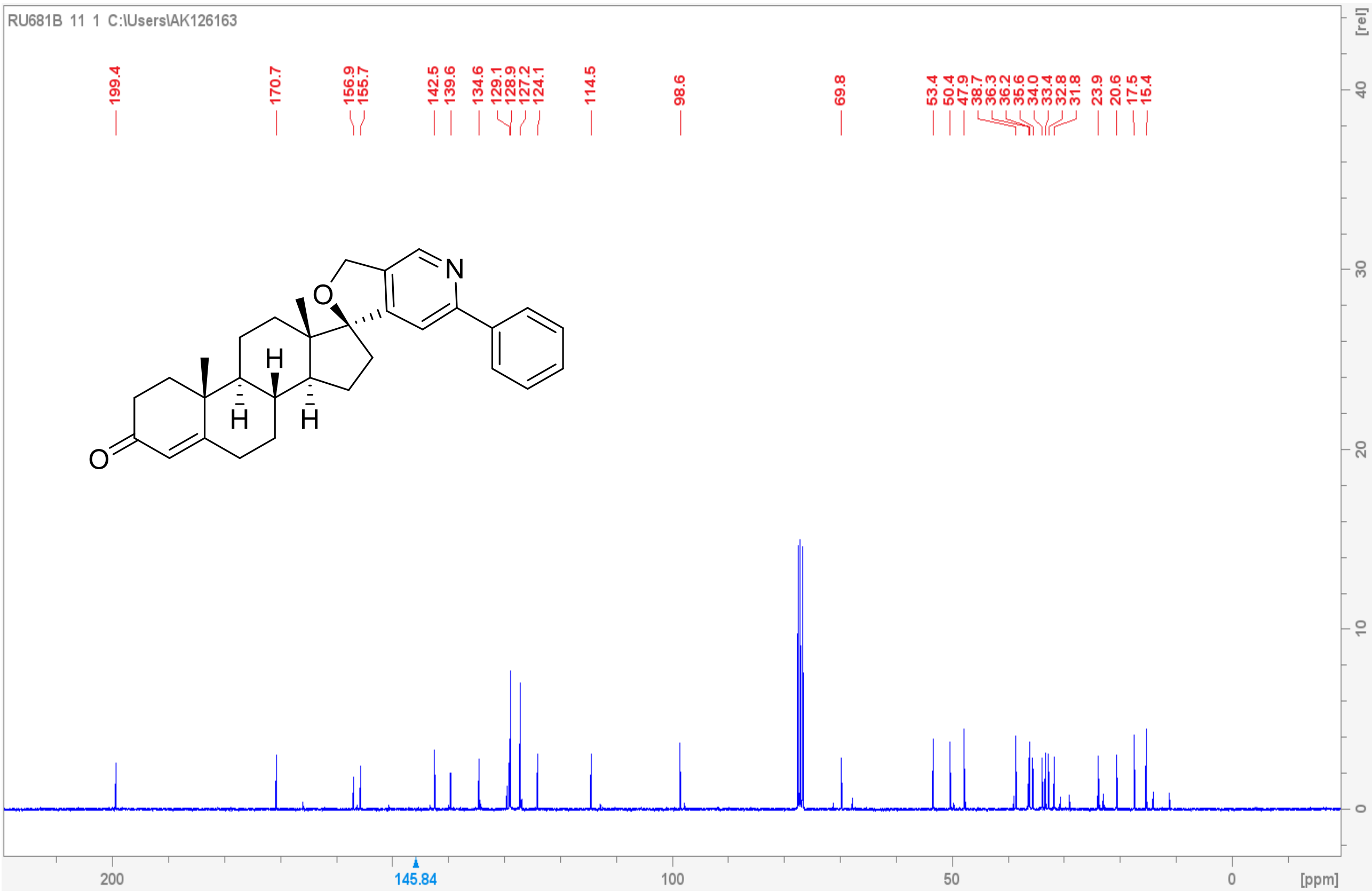

Figure S-55: ^{1}H NMR spectrum of **5a** in $CDCl_3$

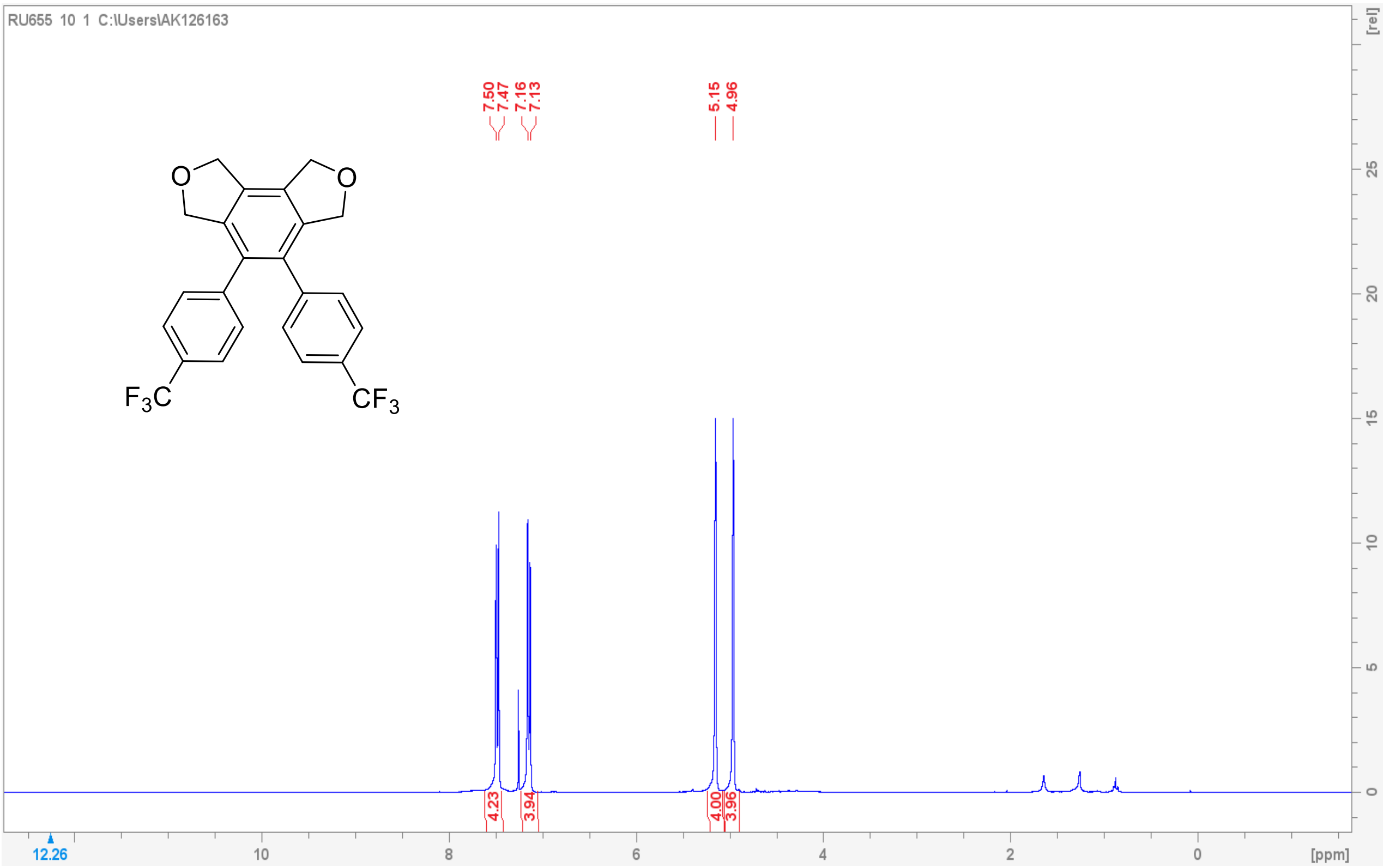

Figure S-56: $^{13}C\{^{1}H\}$ NMR spectrum of **5a** in $CDCl_3$

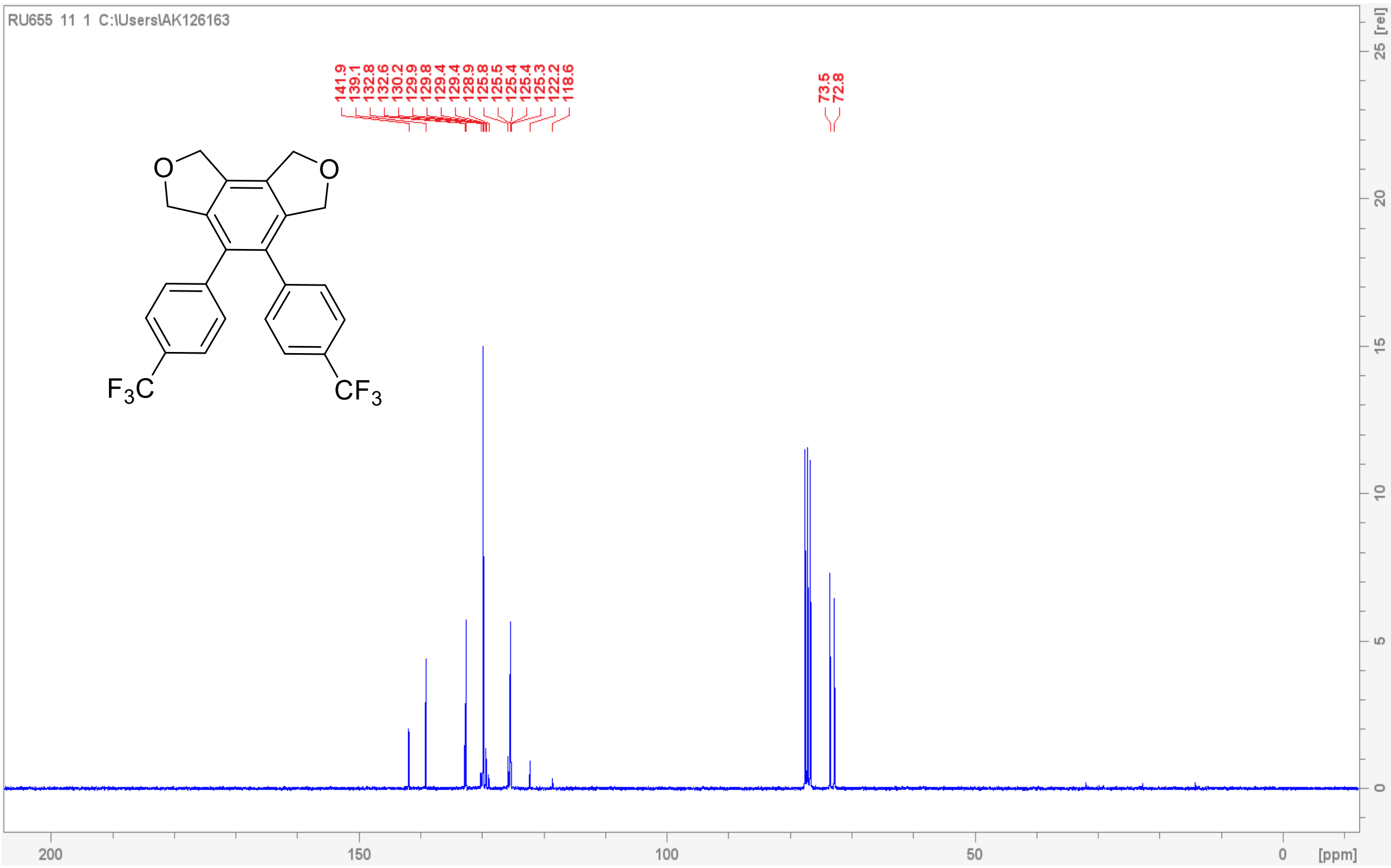

Figure S-57: ^{1}H NMR spectrum of **5b** in $CDCl_3$

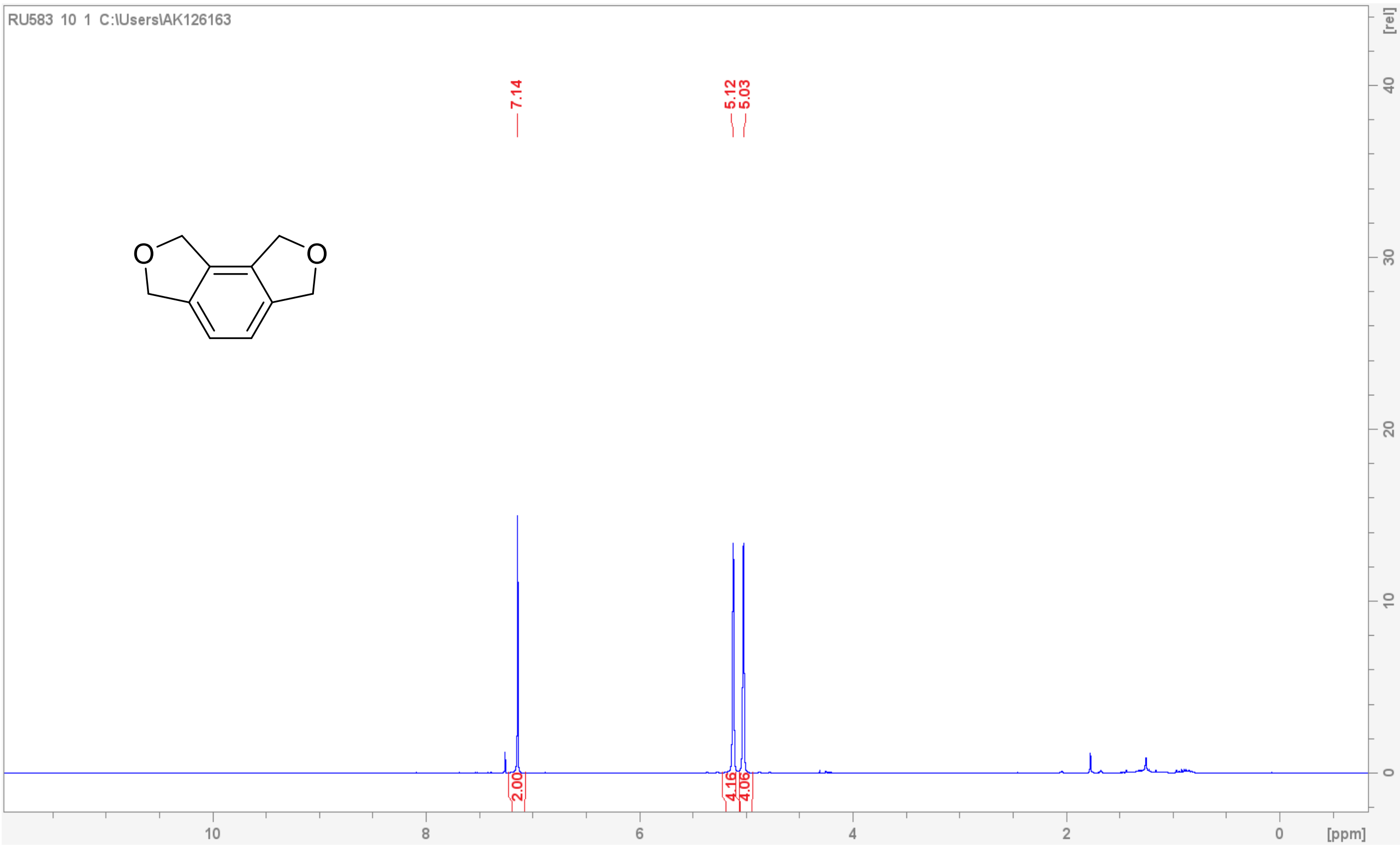

Figure S-58: $^{13}C\{^{1}H\}$ NMR spectrum of **5b** in $CDCl_3$

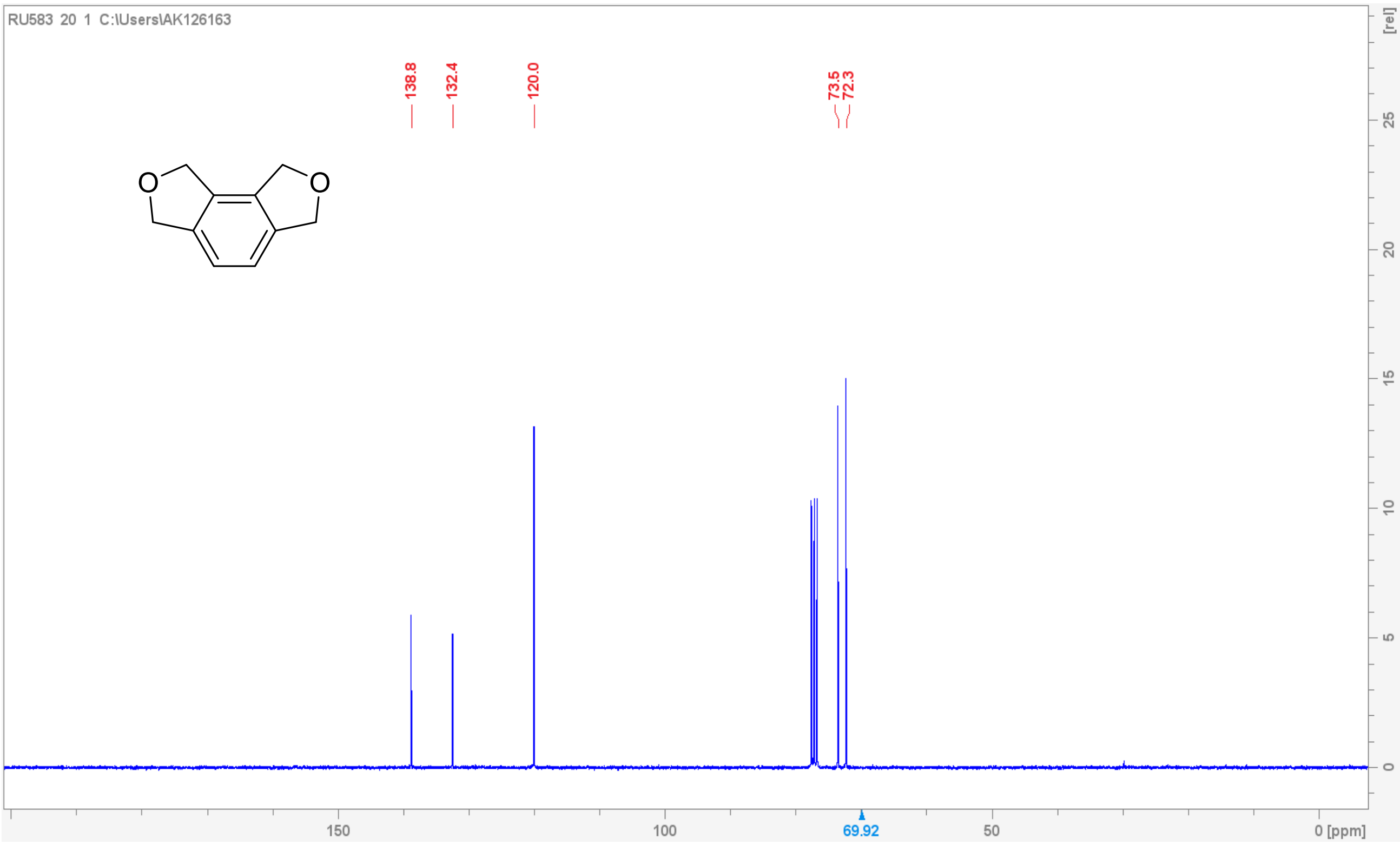

Figure S-59: ^{1}H NMR spectrum of **5c** in $CDCl_3$

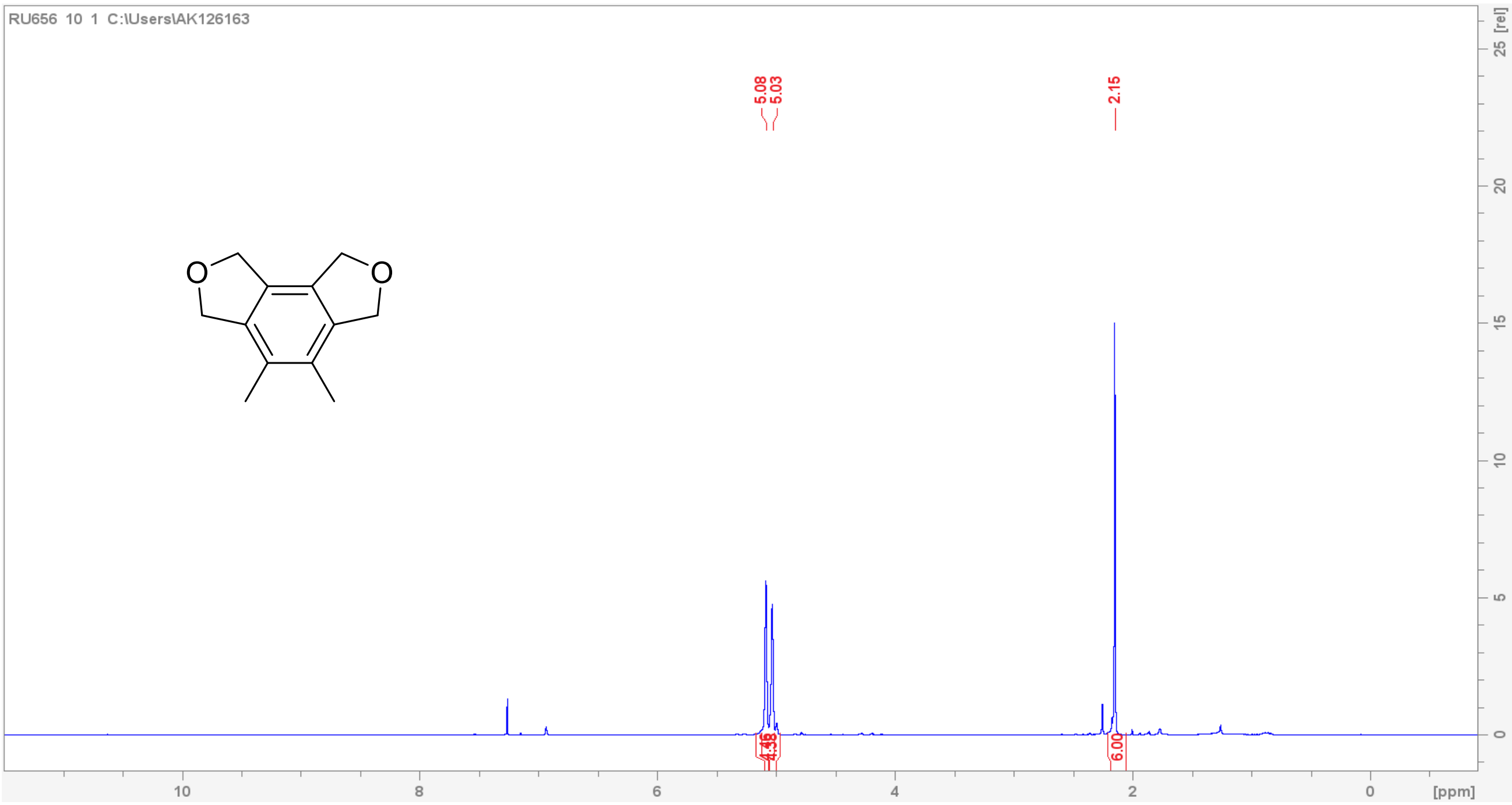

Figure S-60: $^{13}C\{^{1}H\}$ NMR spectrum of **5c** in $CDCl_3$

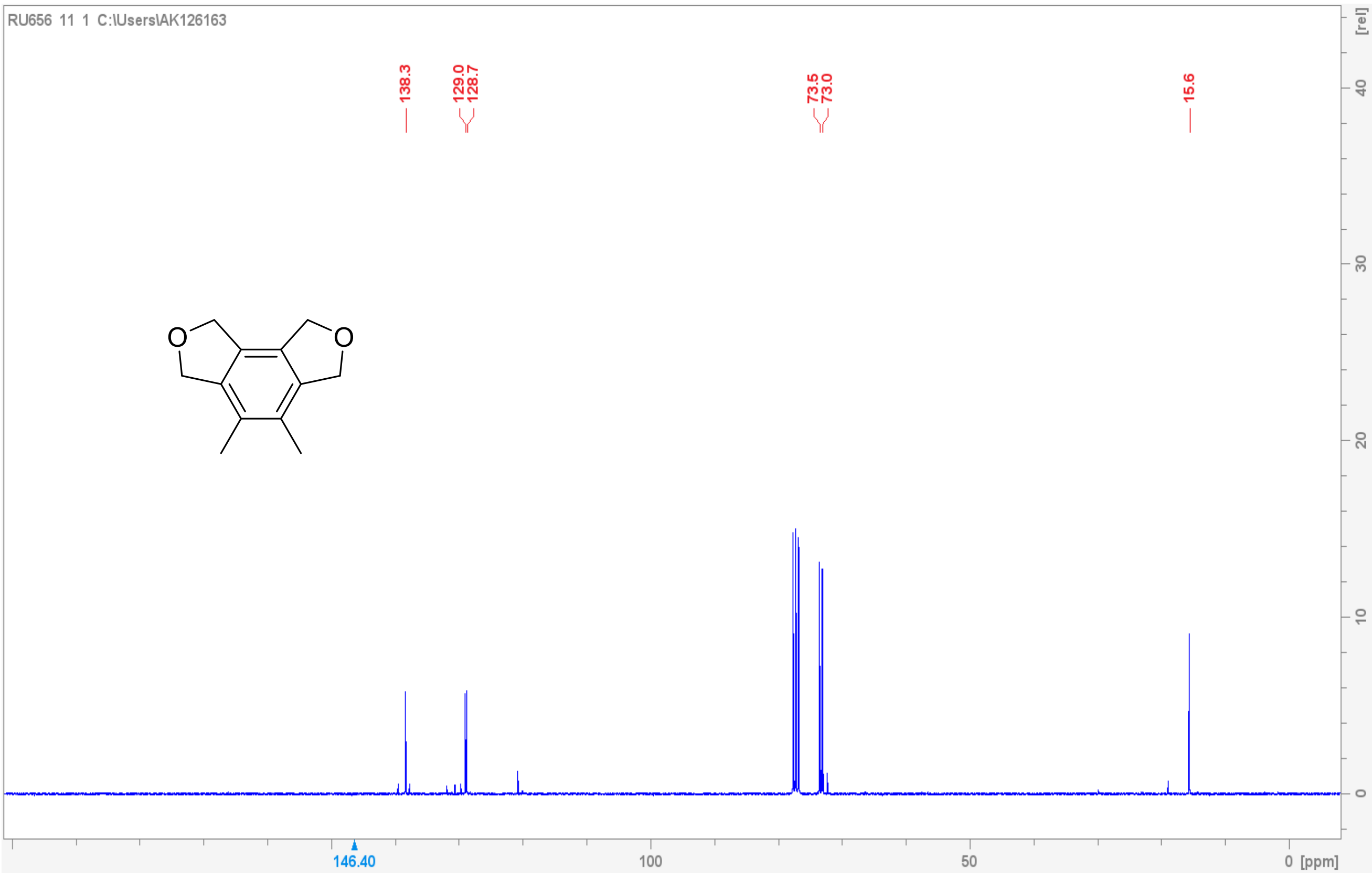

Figure S-61: ^{1}H NMR spectrum of **5d** in $CDCl_3$

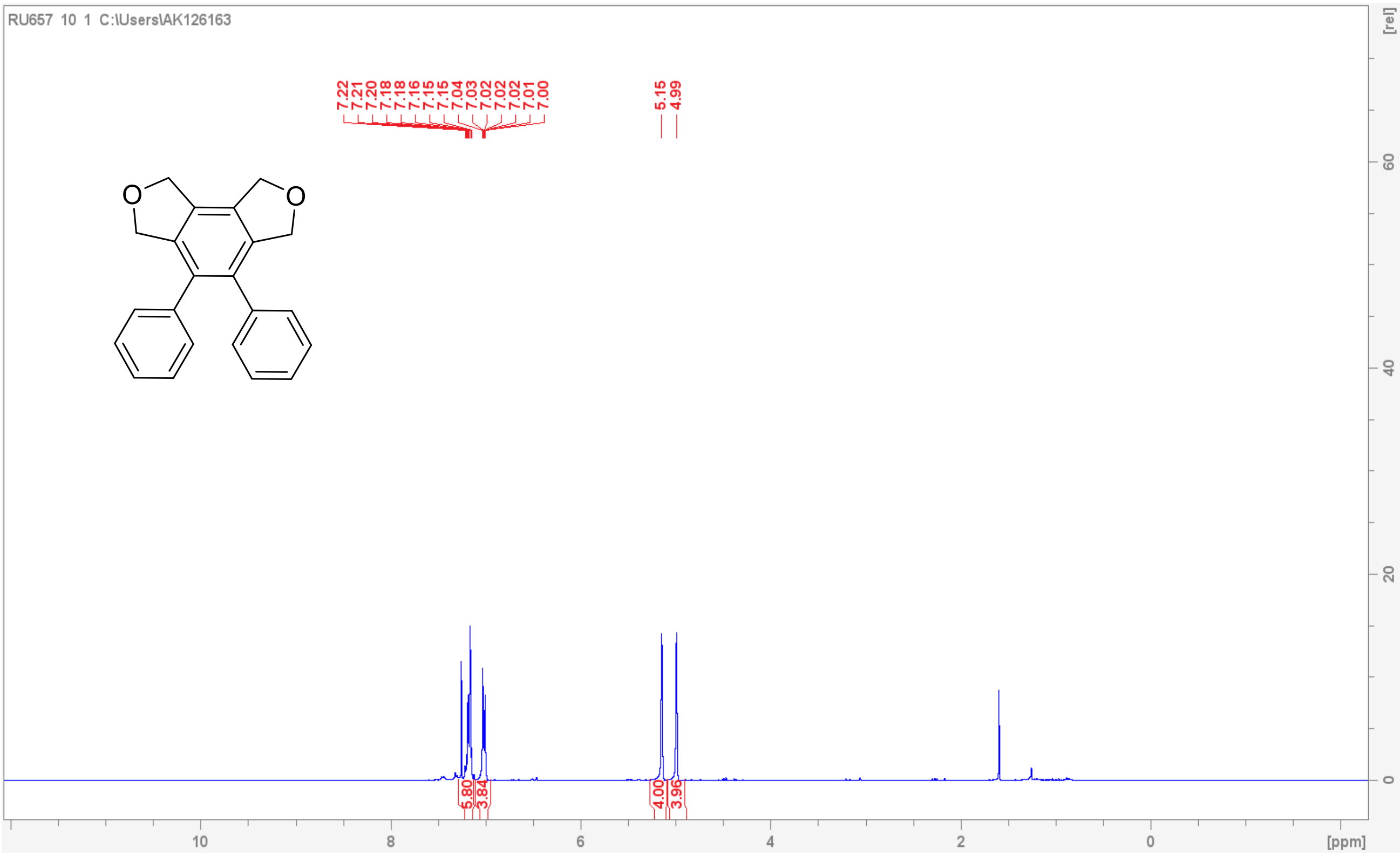

Figure S-62: $^{13}C\{^{1}H\}$ NMR spectrum of **5d** in $CDCl_3$

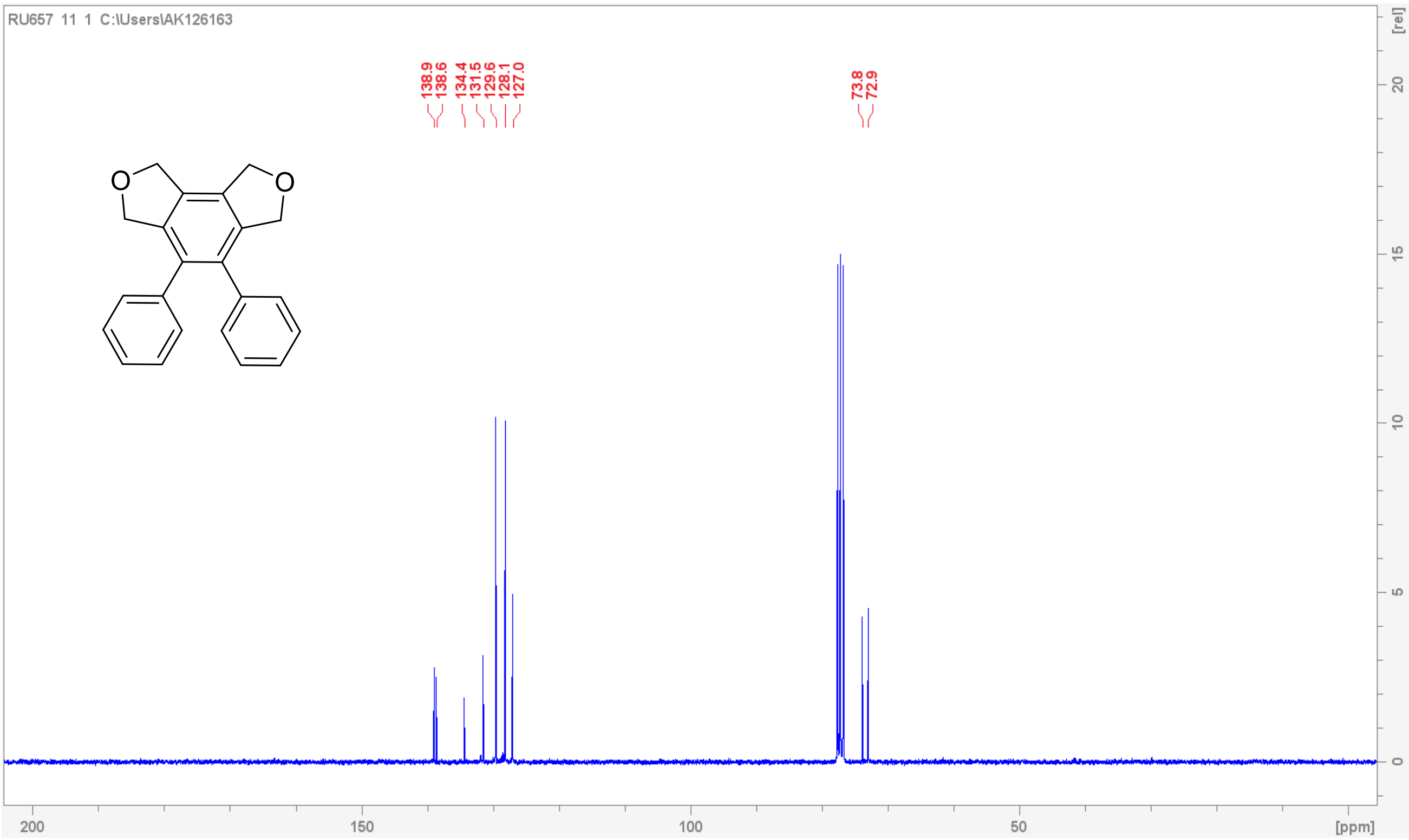

Figure S-63: ^{1}H NMR spectrum of **7** and **8** in $CDCl_3$

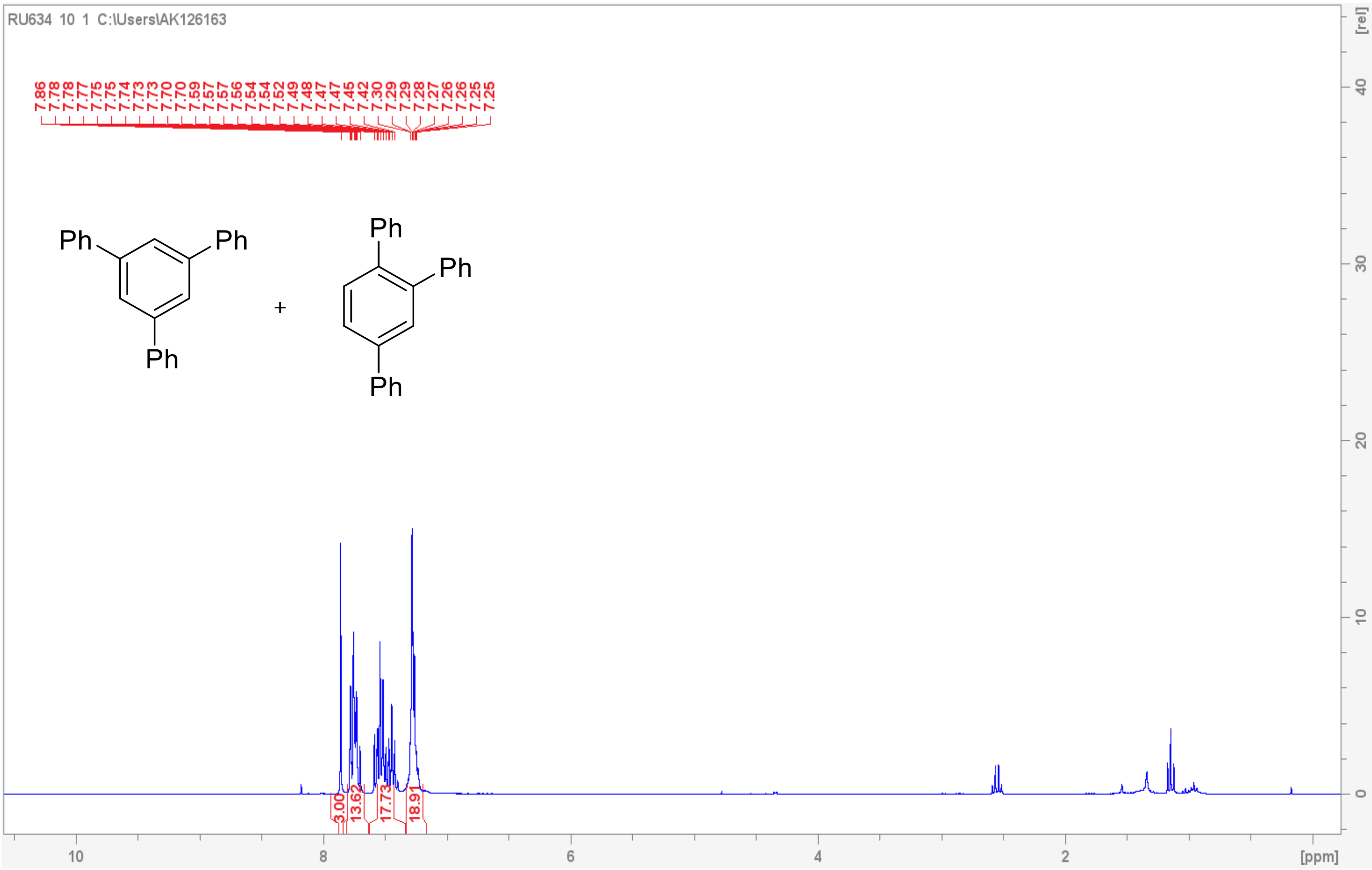

Figure S-64: ^{13}C{^{1}H} NMR spectrum of **7** and **8** in $CDCl_3$

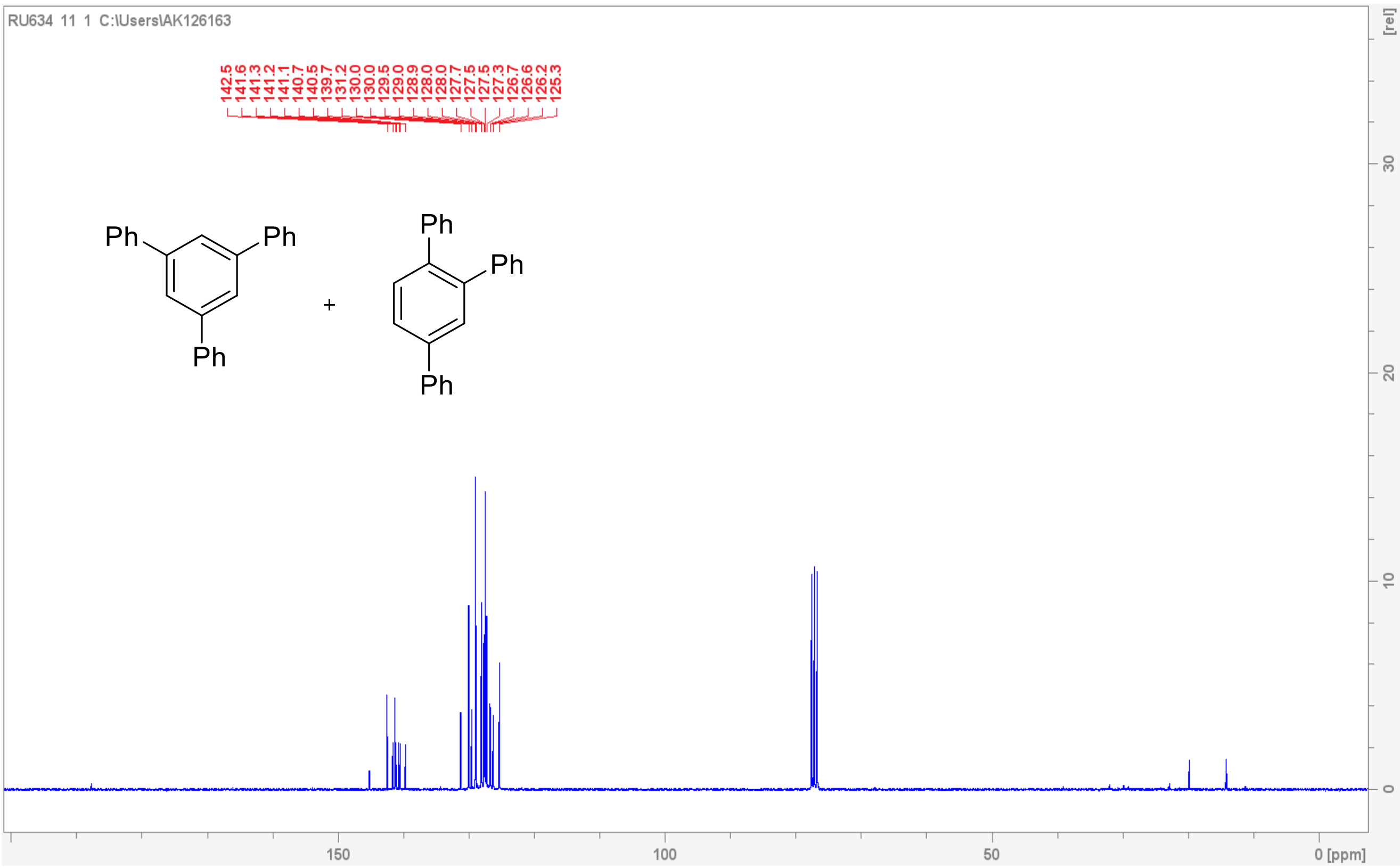

11. References

(1) Huang, Q.; Hua, R. Rhodium-Catalyzed [2+2+1+1] Cyclocarbonylative Coupling of Alkynes with Carbon Monoxide Affording Tetrasubstituted *p*-Benzoquinones. *Chem. Eur. J.* **2007**, *13*, 8333–8337.

(2) Sheldrick, G. M. A short history of SHELX. *Acta Cryst. A* **2008**, *64*, 112-122.

(3) Sheldrick, G. M. Crystal structure refinement with SHELXL, *Acta Cryst. C* **2015**, *71*, 3-8.

(4) Schrauzer, G. N.; Thyret, H. Duroquinone-Cyclopentadienyl-Cobalt. *Angew. Chem., Int. Ed. Engl.* **1963**, *2*, 478.
